# Development of the Psychosocial Rehabilitation Web Application (Psychosocial Rehab App)

**DOI:** 10.3390/nursrep15070228

**Published:** 2025-06-25

**Authors:** Fagner Alfredo Ardisson Cirino Campos, José Carlos Sánches García, Gabriel Lamarca Galdino da Silva, João Antônio Lemos Araújo, Ines Farfán Ulloa, Edilson Carlos Caritá, Fabio Biasotto Feitosa, Marciana Fernandes Moll, Tomás Daniel Menendez Rodriguez, Carla Aparecida Arena Ventura

**Affiliations:** 1 Faculty of Psychology, University of Salamanca (USAL), 37005 Salamanca, Spain; jsanchez@usal.es (J.C.S.G.); inesfarfan@usal.es (I.F.U.); 2 Departments of Psychiatric Nursing and Human Sciences, Ribeirão Preto Nursing School (EERP), São Paulo University (USP), Ribeirão Preto 14040-902, Brazil; 3 Center for Exact, Natural and Technological Sciences, University of Ribeirão Preto (UNAERP), Ribeirao Preto 14096-900, Brazil; gabriel.lasilva@sou.unaerp.edu.br (G.L.G.d.S.); joao.araujo@sou.unaerp.edu.br (J.A.L.A.); ecarita@unaerp.br (E.C.C.); 4 Faculty of Psychology, Federal University of Rondônia (UNIR), Porto Velho 76801-974, Brazil; fabio.depsi@unir.br; 5 Faculty of Nursing, State University of Campinas (UNICAMP), Campinas 13083-970, Brazil; marcfmol@unicamp.br; 6 Mathematics Department, Federal University of Rondônia (UNIR), Porto Velho 76801-974, Brazil; tomas@unir.br

**Keywords:** psychosocial rehabilitation project, psychosocial rehabilitation, web-based mental health app, digital health technology development and mental health

## Abstract

**Introduction:** Few applications worldwide focus on psychosocial rehabilitation, and none specifically address psychosocial rehabilitation projects. This justifies the need for an application to assist mental health professionals in constructing and managing such projects in the Brazilian mental health scenario. **Objective:** This study aimed to present a web application, the “Psychosocial Rehabilitation Application” (Psychosocial Rehab App), and describe its development in detail through a technological survey conducted between May 2024 and February 2025. **Method:** The development process of the web app was carried out in the following four stages, adapted from the Novak method: theoretical basis, requirements survey, prototyping, and development with alpha testing. The active and collaborative participation of the main researcher (a psychiatric nurse) and two undergraduate software engineers, supervised by a software engineer and a professor of nursing and psychology, was essential for producing a suitable operational product available to mental health professionals. Interactions were conducted via video calls, WhatsApp, and email. These interactions were transcribed using the Transkriptor software and inserted into the ATLAS.ti software for thematic analysis. **Results:** The web app “Psychosocial Rehabilitation Application” displays a home screen for registration and other screens structured into the stages of the psychosocial rehabilitation project (assessment, diagnosis, goals, intervention, agreements, and re-assessment). It also has a home screen, a resource screen, and a function screen with options to add a new project, search for a project, or search for mental health support services. These features facilitate the operation and streamline psychosocial rehabilitation projects by mental health professionals. Thematic analysis revealed three themes and seven codes describing the entire development process and interactions among participants in collaborative, interrelational work. A collaborative approach between researchers and developers was essential for translating the complexity of the psychosocial rehabilitation project into practical and usable functionalities for future users, who will be mental health professionals. **Discussion:** The Psychosocial Rehab App was developed collaboratively by mental health professionals and developers. It supports the creation of structured rehabilitation projects, improving decision-making and documentation. Designed for clinical use, the app promotes autonomy and recovery by aligning technology with psychosocial rehabilitation theory and the actual needs of mental health services. **Conclusions:** The Psychosocial Rehab App was developed through collaborative work between mental health and technology professionals. The lead researcher mediated this process to ensure that the app’s functionalities reflected both technical feasibility and therapeutic goals. Empathy and dialog were key to translating complex clinical needs into usable and context-appropriate technological solutions.

## 1. Introduction

The digital mental health landscape has experienced exponential growth, with more than 10,000 applications (apps) developed globally [1,2]. Most of these apps focus on cognitive behavioral therapy techniques aimed at treating anxiety and depression in psychiatric patients. However, tools designed to support Psychosocial Rehabilitation (PR) are scarce [3,4,5,6].

PR is defined as a process that provides psychiatric patients with opportunities to achieve functionality, independence, and autonomy in their communities. This is accomplished by developing individual and collective skills that enable patients to (re)integrate socially, develop autonomy and independence, perform activities, tasks, and social roles, exercise self-determination, and manage their lives [7,8].

Studies have shown that apps based on psychosocial rehabilitation can play a crucial role in fostering autonomy and social functionality, as well as improving the quality of life of psychiatric patients [5]. According to an integrative review that searched the databases of Latin American and Caribbean Literature in Health Sciences (LILACS), Nursing Literature (BDENF), PubMed, Scopus, and Web of Science for requirements for prototyping a psychosocial rehabilitation app, only one app aimed at the Argentinean mental health context was found. This app focuses on psychoeducation for patients, who are its main users, rather than on mental health professionals, who could use it as a tool for psychosocial rehabilitation.

The practical application of the PR theory poses a challenge to mental health professionals owing to its theoretical complexity. This challenge is exacerbated by the barriers inherent in the configuration of the Psychosocial Care Network (RAPS) of the Brazilian public Unified Health System (SUS), which is characterized by fragmented mental health care, disjointed mental health initiatives, and poor communication between professionals and services involved in the psychosocial rehabilitation process [7,9,10,11,12].

The Psychosocial Rehabilitation Project (PRP) organized the principles of the psychosocial rehabilitation theory into a care plan for mental health professionals. The PRP aims to unify and optimize communication between professionals and RAPS devices to address the needs of psychiatric patients in the psychosocial rehabilitation process [8,13].

In this context, an app designed for constructing psychosocial rehabilitation projects has emerged as a technological tool capable of computerizing and streamlining mental health care guided by psychosocial rehabilitation. This app can optimize interprofessional communication and integrate the RAPS into the collaborative environment of psychosocial rehabilitation project planning, assessment, construction, monitoring, and re-assessment phases [2,7,8,14,15].

The development of an app for constructing psychosocial rehabilitation projects in mental health, specifically in the context of the Brazilian public health system, and aimed at mental health professionals, represents a promising tool for supporting the management and health care of psychiatric patients [5,6,8,16,17,18,19,20].

Therefore, this study aimed to present a web application, the “Psychosocial Rehabilitation Application” (Psychosocial Rehab App), and describe its development in detail through a technological survey conducted between May 2024 and February 2025.

Web apps are a type of application defined as applications that run in internet browsers and are characterized by their versatility and ability to be installed on various hardware. Advantages for scientific research include operation via an Internet link, resulting in minimal development and maintenance costs [21,22].

## 2. Method

This was a technological development study to develop the web app Psychosocial Rehab App [23]. This research was approved by the Research Ethics Committee of the Ribeirão Preto School of Nursing (EERP-USP) through Opinion No. 6,605,152 of 3 January 2024 (CAAE: 75372623.5.0000.5393).

The web app Psychosocial Rehab App was developed between May 2024 and February 2025. Physical support was provided by the laboratory of the Study and Research Group in Nursing, Global Health, Law, and Development (GEPESADES), Ribeirão Preto School of Nursing, University of São Paulo (EERP-USP). Virtual support was provided via video call communication channels, such as the Google Meet platform. WhatsApp and email were also used for written communication.

The Psychosocial Rehab App web application was developed in the following four stages, adapted from the Novak method: theoretical basis, requirements survey, prototyping, and development with alpha testing [24]. In the present study, the stage titled “Theoretical foundation of the Psychosocial Rehab App” corresponds to the “Theoretical Basis”; “Prototyping Requirements Survey” corresponds to the “Requirements Survey”; “Prototyping” aligns with the “Prototyping” stage; and “Development with Alpha Testing” refers to the final stage, which maintained the same designation.

The development process essentially involved the active and collaborative participation of the lead researcher (a psychiatric nurse who conceived the project, led, and coordinated the app development process in alignment with the theoretical and practical knowledge of psychosocial rehabilitation), two software engineering undergraduates, a software engineering professor at the University of Ribeirão Preto (Unaerp), and a professor at EERP.

### 2.1. Theoretical Foundation of the Web App Psychosocial Rehab App

First, a narrative literature review was conducted to establish the theoretical foundation for the Psychosocial Rehab App. Based on these results, the theoretical framework was structured to support the development of the web app according to the structure of the psychosocial rehabilitation project and assumptions of psychosocial rehabilitation theory [8].

The web app Psychosocial Rehab App is intended to support mental health professionals in constructing and monitoring psychosocial rehabilitation projects for psychiatric patients, optimizing the processing of mental health information and interconnecting professionals with RAPS devices [14,25].

In this stage of the theoretical foundation, the attributes of the American Psychiatric Association (APA) [26] and the Martinez-Martin, Greely, and Cho (2021) [27] study were articulated as standards oriented toward developing mental health apps, which the Psychosocial Rehab App should meet. These can be observed in Table 1.

### 2.2. Prototyping Requirements Survey

A survey of the prototyping requirements for the Psychosocial Rehab App was conducted using an integrative literature review (ILR). Thirty-six eligible articles and one application were found in national and international databases, and a focus group interview (FGI), which included the participation of eight mental health professionals from a Psychosocial Care Center [13,38].

The ILR raised prototyping requirements, specifically regarding the structure, based on the PRP phases (evaluation, diagnosis and problem identification, therapeutic goals, interventions and actions, agreements and articulations with professional services, and re-assessment). Other requirements include the insertion of a video explaining PR theory and PRP to the user, the ability to locate user support services through GPS, a password-protected login for security purposes, and the ability to view security and privacy policies before creating an account. Additional requirements include the creation of buttons, menus, settings, changeable screens, and profiles, as well as operationalization in the operating system [39].

The FGI surveyed prototyping requirements about technological resources that solve mental health professionals’ difficulties in constructing, managing, and monitoring the psychosocial rehabilitation project. The design allows for the insertion of interventions/activities to be planned and carried out with psychiatric patients individually. It also provides a reminder resource for patients and professionals by sending access links to each patient’s psychosocial rehabilitation project. The PRP stages are articulated with technology, allowing the viewing of patients’ activities and agreements with social authors (mental health services, family members, and professionals, etc.). Reports and interfaces are available for various platforms, as well as physical and electronic components (hardware) [38].

### 2.3. Prototyping

The lead researcher prototyped the Psychosocial Rehab App on the Marvel platform according to the previously raised prototyping requirements. The prototype consisted of 27 screens that graphically represented the login features, mental health professional registration, presentation of the structure for building psychosocial rehabilitation projects, the evaluation process, user support, and credits for the Psychosocial Rehab App (see Supplementary Data S1—Prototype). Additionally, 15 mental health professionals evaluated this prototype, resulting in content validity indices higher than 0.90 for the objectives, appearance, structure, organization, and relevance [13].

### 2.4. Development with Alpha Testing

The development of the Psychosocial Rehab App occurred in two stages. In the first stage, technology professionals and the lead researcher actively and collaboratively interacted in virtual meetings, sharing emotions and knowledge to adapt the previous prototype (see Supplementary Data S1—Prototype). They used Figma software (https://www.figma.com/) for this process (see Supplementary Data S2—Virtual Meetings). This instructional design was highly refined and designed to achieve the initially proposed objectives (see Supplementary Data S3 for the screens of this instructional design). In the second stage, technology professionals developed the app using the Flutter programming language. Dart, a programming language known for its simplicity, efficiency, and support for asynchronous programming, was used to ensure fluidity and high performance. Data storage was performed using Firebase with Cloud Firestore, a nonrelational database offering flexibility and scalability [16,19].

Furthermore, during development a company was contracted to create an animated video explaining the psychosocial rehabilitation project in a playful manner. This video was added to the “Home” page of the app. A lawyer was contracted to develop the Terms of Use/Contract for the app’s end users (mental health professionals). Meanwhile, the lead researcher created the Psychosocial Rehab App user tutorial, which was inserted into the main login screen to guide users.

Table 2 summarizes the architecture of the Psychosocial Rehab App and presents its screens, functions, technological features, content, and database.

It is important to emphasize that the Psychosocial Rehab App was developed based on a prototype that was previously built and validated in two stages by mental health professionals [13]. The present study aimed to maintain alignment with the original prototype (see Supplementary Data S1—Prototype). However, recognizing the creative leaps inherent to technological design, we incorporated innovations that emerged throughout development, as recommended by design scholars [40].

The reflexivity process was continually considered as the lead researcher played a key role in the development as a mental health consultant, which could have introduced biases in data interpretation, design decisions, and the choice of technological resources. The influence of the lead researcher was minimized through collaborative work with developers and constant supervision from professors of technology, psychology, and nursing. This approach ensured greater coherence between the empirical data, technical decisions, and the ethical and political principles of the psychosocial rehabilitation project supported and transmitted by the technology [41].

Furthermore, during the final stages of development, the Psychosocial Rehab App was tested by the lead researcher and the development team to refine the application before its official release. In this phase, essential aspects such as tool functionality, user interface, navigation, system performance, and security were evaluated. Additionally, potential technical errors, communication failures between screens, inconsistencies in the user flow, and compatibility issues with different devices and browsers were identified and corrected. This stage also allowed verification that the app meets the practical needs of mental health professionals, ensuring that the functionalities are intuitive, accessible, and effective to support clinical work. With these improvements, the web app was prepared to provide a stable and reliable experience for its end users [24].

### 2.5. Data Analysis

The audio files produced from the recordings of the meetings, or text messages via WhatsApp and emails, were transcribed using the Transkriptor software (https://transkriptor.com/) and later inserted into the ATLAS.ti software [42,43,44]. The ATLAS.ti software (https://atlasti.com/) organized this written material (the transcripts and other written content can be found in Supplementary Data S4—Field Diary) to be analyzed by the researchers in light of the theoretical framework of Thematic Analysis, according to the following six steps: 1. familiarization with the data; 2. generation of initial codes; 3. search for themes and subthemes; 4. review of themes and subthemes; 5. definition and naming of themes and subthemes; 6. production of the report with results of themes, subthemes, and codes [45,46,47].

Thematic analysis was used to process qualitative data in this study, owing to its methodological flexibility. This approach allowed us to systematically and rigorously identify, analyze, and report recurring patterns in participants’ speeches. This method is particularly well suited for exploring meanings, perceptions, and subjective experiences captured during socio-affective and collaborative interactions between the lead researcher and the technology professionals who developed the Psychosocial Rehab App [45,46,47,48,49,50,51].

To ensure the rigor of the research, a second researcher audited this analysis and found similarities between the themes, codes, and extracts obtained from the research [52].

## 3. Results

**Figure 1:**
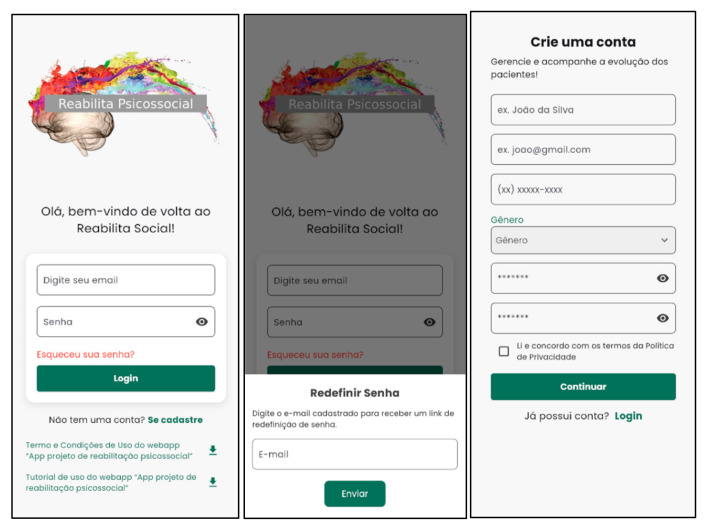
Login screen for mental health professionals. Source: Psychosocial Rehab App (2025).

**Figure 2:**
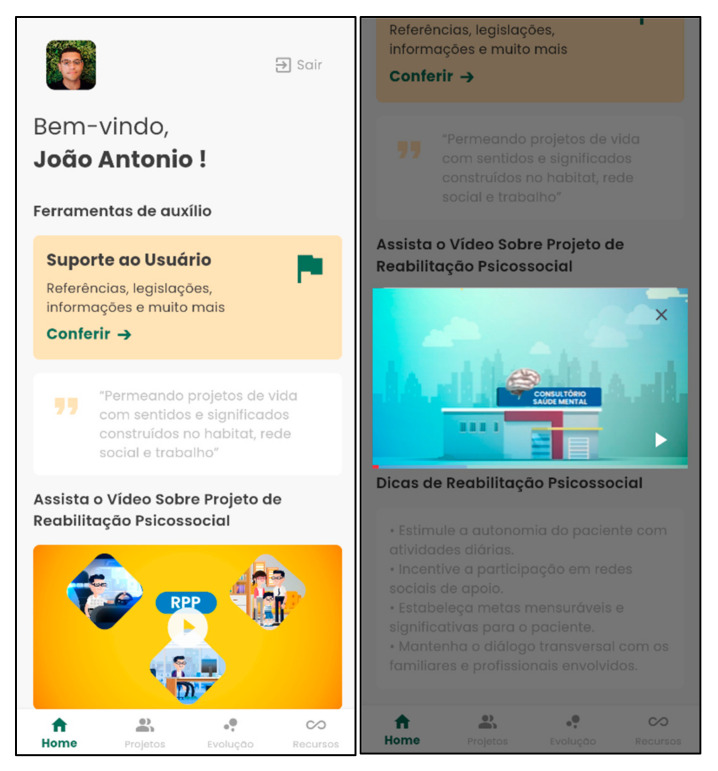
Psychosocial Rehab App home page. Source: Psychosocial Rehab App (2025).

Figure 1 and Figure 2 show examples of the login screen and the home page of the Psychosocial Rehab App, respectively.

**Figure 3:**
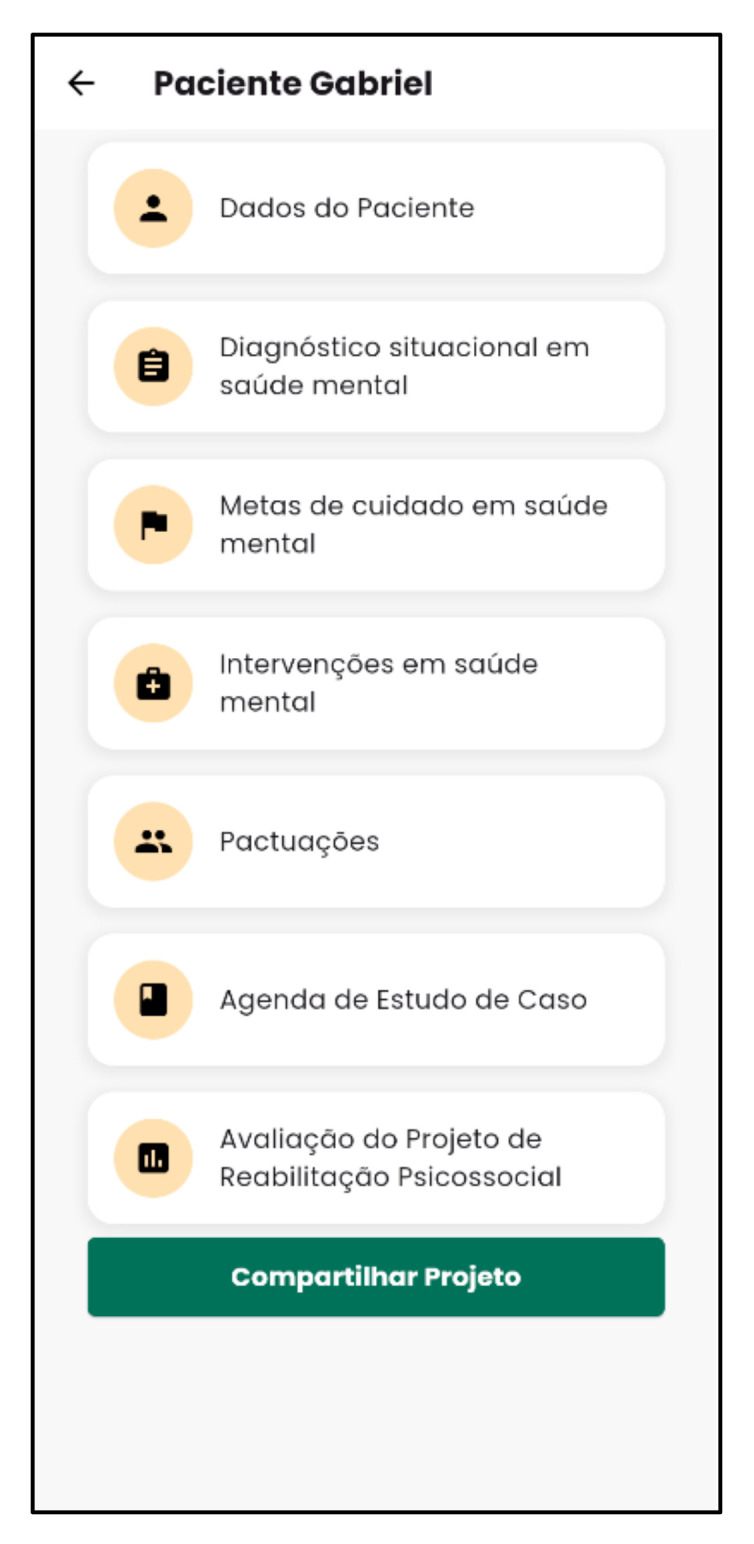
Example of the projects screen of the Psychosocial Rehab App. Source: Psychosocial Rehab App (2025).

**Figure 4:**
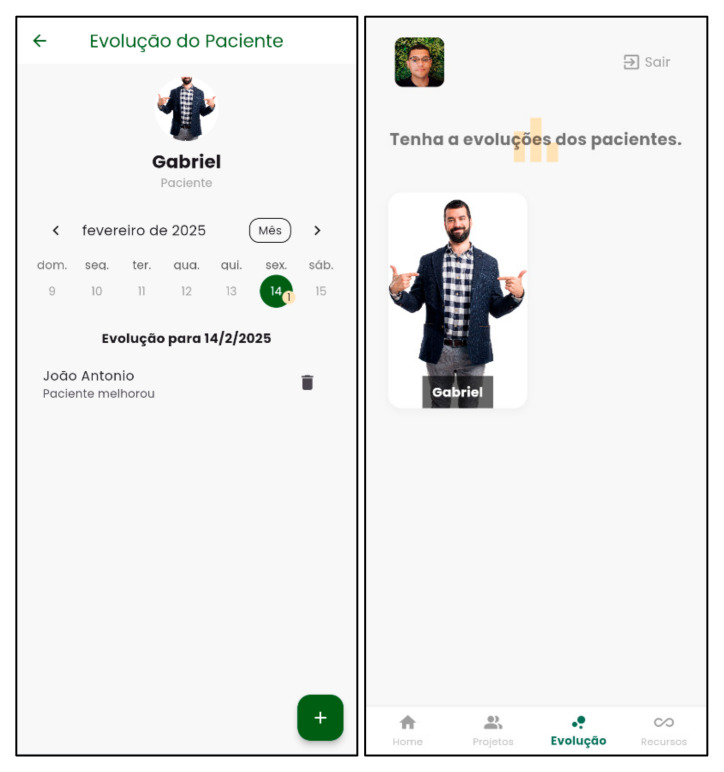
Example of patient progression in the Psychosocial Rehab App. Source: Psychosocial Rehab App (2025).

Figure 3 and Figure 4 demonstrate the structure of the Psychosocial Rehab App for developing the psychosocial rehabilitation project.

**Figure 5:**
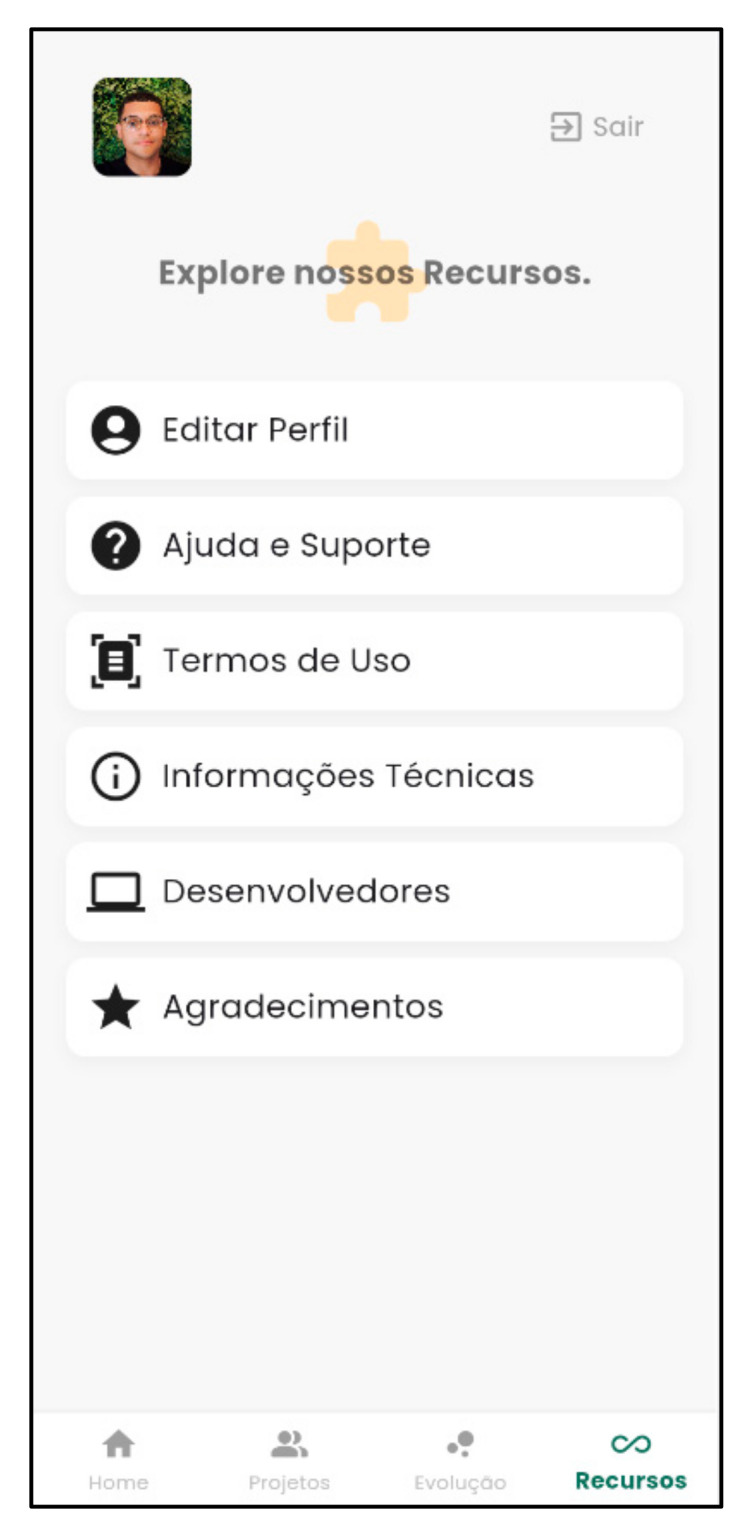
Features of the Psychosocial Rehab App. Source: Psychosocial Rehab App (2025).

**Figure 6:**
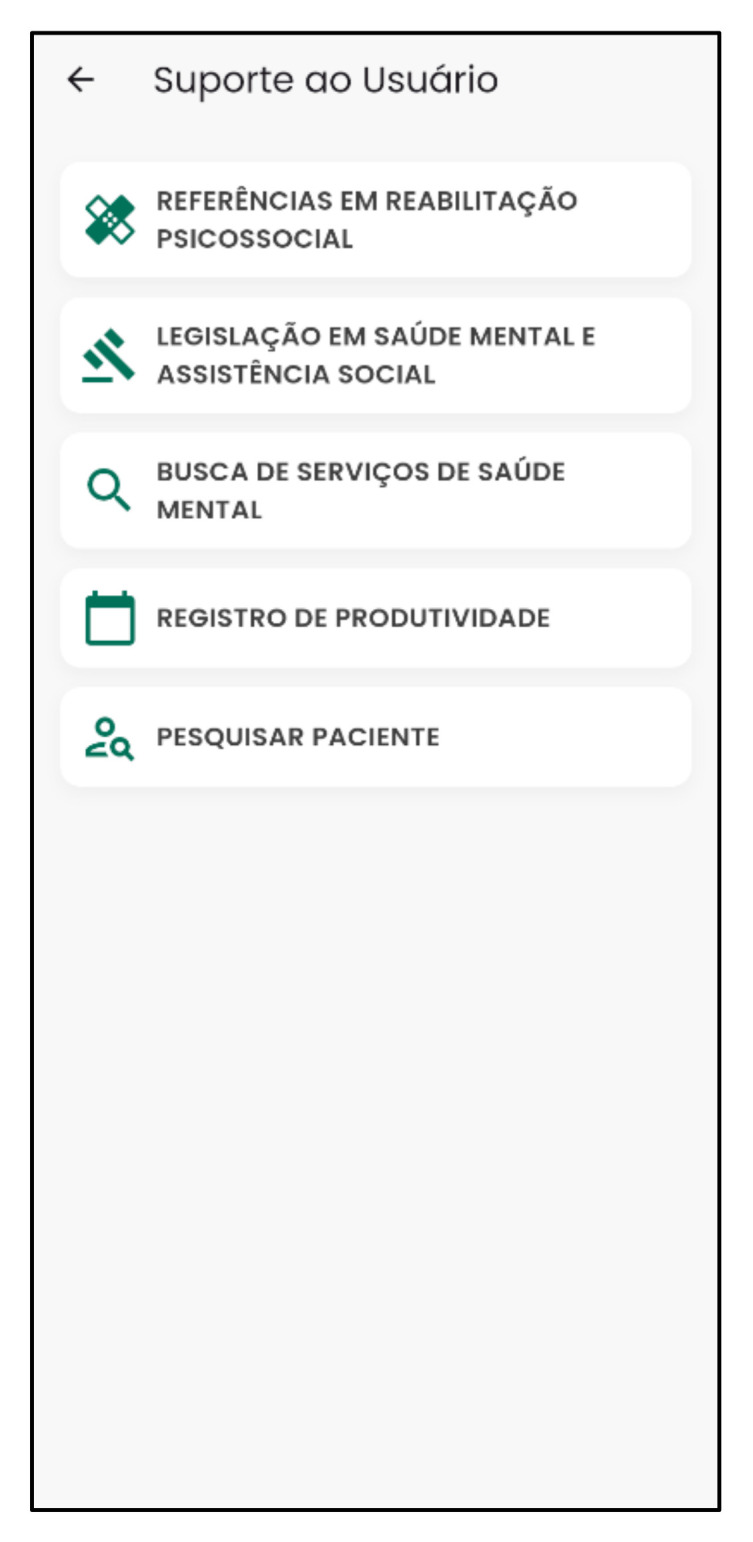
Help and support of the Psychosocial Rehab App. Source: Psychosocial Rehab App (2025).

Figure 5 and Figure 6 present the support features of the Psychosocial Rehab App.

The following themes were developed from the codes derived from the interactions between the lead researcher and technology professionals during the development of the Psychosocial Rehab App. These themes and their related codes are presented in Table 3. They are as follows: 1. constructing and improving the instructional design for the development of the Psychosocial Rehabilitation App; 2. exchanging experience and undertaking collaborative work in the construction of the instructional design and development of the Psychosocial Rehabilitation App; 3. development of the Psychosocial Rehab App with testing and feedback from the lead researcher. A detailed narrative summary of the findings is provided below to clarify the context and insights gathered from the development process.

### Narrative Summary of the Findings in Table 3

During the development of the Psychosocial Rehab App, the lead researcher initially held virtual meetings with technology professionals. He explained and demonstrated the prototype of the Psychosocial Rehab App, which he had designed. During these meetings, he explained the structure of the psychosocial rehabilitation project and its implications for patients receiving psychiatric care from mental health professionals. As the meetings progressed, the developers improved the prototype based on the lead researcher’s feedback, resulting in an instructional design for the development of the Psychosocial Rehab App. The lead researcher was responsible for monitoring, testing, and verifying that the design met the instructional plans and that the product was minimally viable. However, this process was challenging. For example, linking users to their CPF (Brazilian identity document) proved difficult, even after the app was developed. It was difficult to determine when this information would be necessary. Ultimately, the CPF was replaced by the CNS, a mandatory identification number required for anyone using the Brazilian public health system. The CNS is a better choice because it is easier to obtain and involves less bureaucracy than the CPF does. It is only required for those who use the Brazilian public health system and does not restrict foreigners or people in street situations. Human interaction and affection mediated the entire development process, resulting in an exchange of knowledge between mental health professionals and technologists. This was a rich and transformative learning experience. This occurred from the creation of the instructional design to the adjustment of technological functions during the stages of the psychosocial rehabilitation project. One example of this is the interaction regarding the explanation of “design” and “progression.”

## 4. Discussion

### 4.1. Constructing and Improving the Instructional Design for the Development of the Psychosocial Rehab App

Developing any application, including web apps, which are apps accessed via the Internet and are compatible with different operating systems [20,22], requires the interactive participation of the lead researcher, who acts as a co-designer and mental health specialist [53]. This researcher serves as a bridge between the needs of users (other mental health professionals) and developers [17,19,54,55], demanding flexibility, interpersonal skills, prototyping, engagement, and collaboration [56].

Other studies corroborate the development of mental health apps with the active and collaborative participation between a lead researcher, who is a mental health professional, and technology professionals [16,17,19].

The researcher ensures that the app under development aligns with the desired therapeutic purpose [53], and promotes an environment of continuous collaboration and interdisciplinary work [40]. The researcher mediates the “translation” of the needs of mental health professionals to technology professionals who develop mental health apps. In this study, the “translation” for developing the Psychosocial Rehab App was challenging and complex when determining which technological resources would solve the difficulties of constructing psychosocial rehabilitation projects [16,56].

Making people understand the purpose of health apps, the research phenomenon, and the social objects involved, and their impact on users’ lives in resolving work difficulties [56,57], interaction and collaboration are required in a dialogical process to improve the software and its functionalities from the initial design stage [57]. This approach helps us avoid “translation failures” between the developers’ understanding and the researchers’ perception of what is important for solving difficulties faced by mental health professionals [56].

Instructional design involves developers creating the app’s layout, elements, and tools, and programming it using a programming language [23]. This instructional design materialized the prototype’s improvement changes and reflected them in the Psychosocial Rehab App. These changes improved appearance, layout, and organization. The goal was to create an application with minimal content that was coherent with technological support and linked to the theoretical structure of the psychosocial rehabilitation project and the principles of psychosocial rehabilitation theory [7,8,18,28].

Improving the prototype, construction, and modification of projects in development (i.e., instructional design) are all common in research on the development of mental health apps [6,16,23]. These processes seem to be related to development studies that prioritize “participatory designs,” which involve intense collaboration between researchers, mental health professionals, and developers [6,18]. These can occur whenever necessary, even after the app is finished, to ensure the feasibility of mental health apps [16].

In contrast to the present study, the Artemis-A app (early identification of mental health difficulties in students) was developed by a specialized company [54]. Nevertheless, the present study also used support services, such as hiring legal services and creating an animated video, to make the Psychosocial Rehab App attractive and viable for marketing to end users [54].

### 4.2. Exchange of Knowledge and Collaborative Work in the Construction of the Instructional Design and Development of the Psychosocial Rehab App

Technologies reflect the relationships between people and the objects they work with [58]. Because it is a relational process, professionals and researchers involved in developing mental health apps must consider human interaction as part of collaborative work which involves significant learning and the exchange of experience and knowledge [40]. The study that developed the Appa Health app to treat anxiety and depression in adolescents demonstrated collaborative interaction among those involved in developing the app based on cognitive behavioral therapy theory [55].

Another study demonstrated interactive and collaborative participation among participants in developing the Mentallys app, which facilitates access to mental health care. Throughout the development process, the aforementioned study prioritized creating an environment that fosters creativity as well as a creative leap between researchers and developers. The study also emphasized the construction of a continuous collaborative work environment [40].

The collaborative creation between the lead researcher and technology professionals participating in this study on the instructional design for the development of the Psychosocial Rehab App led to the participation of the lead researcher in its development. His contributions included the contextualization and applicability of this application to his professional practice [16], and aligning it with the construction and management of psychosocial rehabilitation projects by mental health professionals [53].

In general, researchers in the mental health field do not master the “professional” tools necessary for app development [16], but bring their natural creativity and experiential knowledge about mental health, which involves explaining causes, effects, and treatments [59,60]. In contrast, technology professionals contribute to “taking ideas out of the project and making them functional with a high probability of usability,” resulting in structured mental health apps that are minimally viable technological products ready for use and commercialization [40]. Moreover, technology professionals must be committed to listening to and empathetically validating suggestions from participants, so the instructional design reflects therapeutic purposes, achieving a balance and intersection between mental health and technology [56].

### 4.3. Development of the Psychosocial Rehab App with Testing and Feedback from the Lead Researcher

The Psychosocial Rehab App aligns with the needs of mental health professionals by providing resources that facilitate clinical decision-making and streamline diagnosis, patient registration, information sharing, and data storage [20].

Before logging in and registering, the Psychosocial Rehab App presents the terms of use to make professionals aware of the guidelines and responsibilities regarding use and data sharing to protect psychiatric patients [27,60,61]. Furthermore, an explanatory tutorial on the main login screen guides these professionals through the app’s functions and features to help them build psychosocial rehabilitation projects and avoid feeling lost during initial access and subsequent use [62,63].

The command bar contains the main features of the Psychosocial Rehab App, which is composed of “Home Page,” “Projects, “Progression,” and “Features.” The “Home Page” is the main screen of the app and contains a video about the psychosocial rehabilitation project [8,39].

The “Projects” feature is central to the structure of the Psychosocial Rehab App because it supports the creation of psychosocial rehabilitation projects for psychiatric patients under the care of a mental health professional responsible for their case management [8].

The “Features” command is a crucial tool that assists mental health professionals who use it [58]. It has a strategic “Help and Support” function with tools that help professionals launch their productivity records more dynamically. This is important for adding social value to mental health services [64,65], knowing the main mental health legislation and literature on psychosocial rehabilitation, seeking mental health services, and sharing patient data. These resources are in line with a study showing that resources/tools supporting mental health professionals influence the adoption of mental health apps [63].

“Progression” is documented by mental health professionals in a linear text about the care and interventions provided to psychiatric patients. This periodic report documents changes aligned with therapeutic goals and interventions, enabling the monitoring of the progression or regression of psychiatric patients under the care of a mental health professional [66].

Functions such as “search for mental health services,” “search for the patient/project,” or “add new projects” are crucial to the Psychosocial Rehab App’s purpose of managing the psychosocial rehabilitation project and its dynamism. These functions facilitate the entry and retrieval of clinical histories and access to information about the course of psychosocial rehabilitation projects. They also facilitate communication between mental health professionals and the construction of social networks committed to and responsible for the actions of psychosocial rehabilitation projects [7,21].

Studies on the development of mental health apps have prioritized support functions for mental health professionals [17], patient search resources [21], and aspects related to aesthetics and layout [17,18,19].

Testing mental health apps and receiving feedback from mental health professionals on their use are important for making the app desirable to end users [13,17,58,67]. Mental health professionals seek “easy-to-use” apps that are personalized and adapted to mental health work contexts to solve their problems [55,63].

From the perspective of researchers and technology professionals, creating a common mental health app and mobilizing technological resources to facilitate mental health professionals’ tasks is challenging [20,40].

In fact, conflicts and frustrations arise from the differences in perspectives between developers and health professionals because of their different fields of knowledge, especially when they realize that an idealized project differs greatly from what is actually feasible [40,57]. Technology professionals know how to make the app work technologically, but mental health professionals seek meaning and purpose in technological functioning [13,16,68]. Collaborative work permeated by interdisciplinarity and guided by a balance between creativity, desirability, feasibility, and costs facilitates conflict resolution [40].

#### 4.3.1. Use of the Psychosocial Rehab App

The Psychosocial Rehab App was designed to be used by health professionals, specifically psychiatric nurses, psychiatrists, occupational therapists, psychologists, and social workers, in the context of mental health with a focus on psychosocial rehabilitation. The app structures the phases of the psychosocial rehabilitation project into a systematized care plan that allows for the collection of patient data, identification of problems that influence their recovery and rehabilitation (structured anamnesis, patient potentialities, and difficulties/limitations). The app aims to permeate the psychosocial rehabilitation process by focusing on building possibilities, developing social skills and autonomy, promoting citizenship, strengthening potentialities, and changing structures (environmental, family, political, and service-related) that hinder the patient’s full development, recovery, and construction of life meanings.

#### 4.3.2. Potentialities, Future Directions, and Limitations of the Psychosocial Rehab App

The Psychosocial Rehab App is a web application with strong potential to become an innovative technology within the Brazilian mental health context as it is specifically designed to support the construction of psychosocial rehabilitation projects aligned with the theoretical foundations of this approach. To date, no other app with similar features has been identified, highlighting its originality and innovation. This distinction broadens its potential for use in other contexts, including future translation and adaptation into Spanish and English.

The authors present preliminary data on the content, appearance, and usability of the Psychosocial Rehab App, which demonstrates its practical value. These evaluations were conducted in parallel with the manuscript development and involved mental health professionals as expert judges and information technology specialists who applied the Nielsen heuristics. In the first round of validation, the content validity index (CVI) scores ranged from 0.57 to 1.00, averaging 0.85. In the second round, the scores increased to between 0.80 and 1.00, averaging 0.98, reflecting substantial improvement. Ten usability issues were identified, six of which were of moderate severity (level 3), and one was critical (level 4). The usability-related problems were corrected, whereas aesthetic issues were left for future refinement. The overall usability score was 0.674 (SD: 1.181; CI: [−0.507, 1.855]), indicating a satisfactory usability.

Finally, this research has some limitations. Although the development process was collaborative and considered the needs of mental health professionals, the Psychosocial Rehab App was not implemented in their real-world environment where its effectiveness could be evaluated based on attributes such as acceptability, feasibility, satisfaction, tolerability, adherence, and engagement [69].

## 5. Conclusions

The development of the Psychosocial Rehab App was characterized by a collaborative approach. The lead researcher served as a liaison between mental health professionals, who had practical needs regarding the management and construction of psychosocial rehabilitation projects, and technology professionals, who had the technical capabilities to address these needs.

Horizontal dialog and mutual empathy are essential for translating the complexity of psychosocial rehabilitation projects into coherent and usable functionalities within professional environments. The adjustments made to the instructional design of the development, the functionalities, and the technological resources of the Psychosocial Rehab App highlight the importance of adaptability. These adjustments ensure that the app reflects the technical demands and the theoretical and therapeutic purposes of the psychosocial rehabilitation project.

## Figures and Tables

**Table 1:** Adapted APA guidelines [26] and recommendations from the study by Martinez-Martin, Greely, and Cho (2021) [27] as guidelines for the development of the web app Psychosocial Rehab App.

APA Guidelines	Psychosocial Rehab App	Recommendations from the Study by Martinez-Martin, Greely, and Cho (2021) [27]
Evaluation of Evidence	Psychosocial rehabilitation project theory and principles of psychosocial rehabilitation theory [7,8,28].	Evidence and Validity
Psychosocial Rehabilitation Theory [8]	A process that provides opportunities and/or facilitates (as well as supports and/or develops) psychiatric patients to achieve autonomy, independence, social functionality, a sense of purpose, social (re)insertion, and exercise of citizenship. This process utilizes individual and collective resources to benefit patients without violating their human rights.	
Assumptions of the Psychosocial Rehabilitation Theory [8]	- Psychosocial rehabilitation as a process that provides psychiatric patients with opportunities to achieve autonomy, social functionality, self-determination, and direction in their lives. It also enables them to develop contractuality, empowerment, the exercise of citizenship, and social protagonism. - Psychosocial rehabilitation as an instrument for the (re)construction and (re)discovery of subjectivity in psychiatric patients, the occupation of neglected social spaces, and the construction of socio-affective relationships with emotional significance. - Psychosocial rehabilitation must be contextualized according to the demands and needs of psychiatric patients. - Psychosocial rehabilitation is operationalized through the articulation of the following three life production scenarios: habitat, social networks, and work. - Psychosocial rehabilitation guides mental health professionals in planning and building psychosocial rehabilitation projects that address the psychosocial requirements of users of mental health services.	
Psychosocial Rehabilitation Project [8]	It is a systematized method of managing care and providing assistance for users of mental health services. Based on the psychosocial rehabilitation process, mental health professionals can diagnose problems, psychosocial needs, and demands of psychiatric patients. It enables professionals to plan and manage care, intervene, and mobilize resources within the psychosocial care network and/or community. Professionals can also make agreements and take responsibility for the care provided to the patients. They can monitor, (re)evaluate, and provide personalized, comprehensive, and humanistic assistance that focuses on full citizenship.	
Assumptions of the Psychosocial Rehabilitation Project [8]	- The psychosocial rehabilitation project facilitates the psychosocial rehabilitation of psychiatric patients. - The psychosocial rehabilitation project enables the construction of contractuality in psychiatric patients through the development of care strategies that respond to their biological, psychosocial, emotional, and socioeconomic demands and needs. - The internal structure of the psychosocial rehabilitation project is determined by mental health assessments, therapeutic goals, interventions, division of responsibilities, and re-assessment. - Individual case management facilitates the implementation of the psychosocial rehabilitation project through dialog, teamwork, case management, mobilization of Psychosocial Care Network resources, community, and re-assessment of complex mental health cases.	
Risk Assessment, Privacy, and Security	- Contract for the use of the Psychosocial Rehab App, which is made available to users before purchase. This contract protects the security and privacy of data [29]. - Encryption [30].	Security and Privacy
		Transparency
		Responsibility
Evaluation of ease of use	Validation of content, appearance, and usability with mental health professionals [31,32].	
Interoperability/Interface	Usability inspection (validation) using Nielsen heuristics with technology and information professionals [33].	
	Clear, objective, and stigma-free language and functionalities adaptable to the user’s device [27,34,35,36,37].	Social Justice
Capacity to gather basic information/data	The Psychosocial Rehab App adapts the stages of the psychosocial rehabilitation project, allowing mental health professionals to collect information and data from psychiatric patients [7,28].	

Source: Prepared by the authors (2024).

**Table 2:** Presentation of the Psychosocial Rehab App architecture.

Screens	Start with user registration by accepting the Terms and Conditions of Use of the Psychosocial Rehab App			Mental health professional			
	Tutorial on how to use the Psychosocial Rehab App						
	Home	Short video about the psychosocial rehabilitation project (PRP)					
	Features	Edit Profile	Help and Support	Terms of Use	Technical Information	Developers	Acknowledgements
	Psychosocial Rehabilitation Project (See “add or search” function)		Patient Data (Registration)		Main Description		
					Name, age, address, profession, income, support network, and reference technician.		
			Situational Diagnosis in Mental Health		Case history and multidisciplinary mental health diagnoses; individual resources and skills; potential, desires, and dreams; personal, collective, and structural difficulties; medications in use; clinical diseases; and other relevant information and identified problems.		
			Mental Health Care Goals		Short-term goals (less than 2 months), medium-term goals (6 to 12 months), and long-term goals (more than 12 months).		
			Mental Health Interventions		Problems, interventions, responsible parties, goals, and deadlines.		
			Agreements		Patient, family, reference technician, RAPS, others, interventions, and deadlines.		
			Case Study Agenda		Meeting date and time, agenda, and who is required to attend.		
			PRP Scheduled Assessment		Intervention/agreement, responsible party, compliance status, and observations.		
			Progression		Date and progression (spaces for inserting the progression text).		
Functions	Share	Through the links, generate a PDF (print or save on hardware/cloud) or share via WhatsApp, email, etc.					
	Mental Health Support Services	Search for RAPS services using the patient’s address.					
	Add New Psychosocial Rehabilitation Project	Add a “New Psychosocial Rehabilitation Project” using patient data.					
	Search Patient/Project	Search for a patient’s project by name to easily find it among several other projects.					
Database	Firebase (offers a set of tools that simplify app development. These tools allow developers to focus on the logic and design of their apps, instead of worrying about their infrastructure).						

Source: Prepared by the authors (2024).

**Table 3:** Themes, codes, and extracts of the interactions between the lead researcher and technology professionals during the development of the Psychosocial Rehab App.

Themes	Codes	Extracts
***Constructing and improving the instructional design for the development of the Psychosocial Rehabilitation App***	***Code:*** *“Translation” and clarification of the prototype* *carried out by the lead researcher to technology professionals*	*(…)-That’s my question. In “inserting the new psychosocial rehab project”, are the psychosocial rehabilitation projects patients? (ICT Professional 1).-Yes, they are patients. But in the sense (…) that for example, when a psychosocial rehab project is being created, when “inserting the new psychosocial rehab project” (…), the mental health professional inserts this project that is about the patient, and the idea is that the patient, their name and the project that is under construction or completed will appear (Lead Researcher).-Okay! Let’s examine if I understand this. So, the patient represents a psychosocial rehab project? (ICT Professional 1). (…)-This is a psychosocial rehab project for each patient, (…), so each patient will have a psychosocial rehab project, (…), which (…) (has) this structure (…): patient data, situational diagnosis, goals, interventions, agreements, case study, and evaluation agenda (Lead Researcher).*
	***Code:*** *Improvements made by developers to the prototype in a collaborative context*	*(…)-If we change the design, but keep all the features, is that okay? (ICT Professional 1).* *(…)-No problem! (Lead Researcher).-Perfect, then. So, we can leave the home page as a presentation of the app (…). This Start button here; would it be a kind of Start for the videos?!-(ICT Professional 1).-This part; it was just to show that this part of starting the Start… Now I realize that it doesn’t make sense… (…). Go back to the home page. (which) (…) was really cool. Let’s just keep it short. Let’s leave just one video. That way we won’t leave much information… (Lead Researcher).*
	***Code:*** *Instructional Design for the Development of the Psychosocial Rehab App*	*(…)-We create the screen; we create its function. And then, for example, this one here, which is a screen that is under construction, we now start by taking so many details of it, we will first make it work, (…) then, you can use it as if it were really the app. What you can’t do, for example, is a field; you can’t write in it, precisely because it is a prototype, you know, it doesn’t have that type of function. For example, profile, exit, you can see the flow, what it is like, (…) here we already have a shadow of things; we have the cards (ICT Professional 1).*
***Exchange of experience and collaborative work in the construction of the instructional design and development of the Psychosocial Rehabilitation App***	***Code:*** *Exchange of knowledge about mental health and technology*	*- (…) That’s why we’re talking about it, you know?! Because we’re really going to, we know that it’s not your responsibility, but the technology part, and that you’re more in that area. We’re going to take your idea and we’re going to bring it to technology. (…) (For example) This little screen here (calls the name of the lead researcher), it’s called Drawer. What’s Drawer? Drawer is when the person or the health professional clicks on their photo. And then this screen appears, it scrolls vertically, and the user’s profile information appears (…). (ICT Professional 1). (…) (Then the psychosocial rehab project is explained) it’s not limited to just what’s happening; it also considers the macro level, in establishing goals and interventions for the psychosocial rehab and quality of life of the patient (Lead Researcher).*
	***Code:*** *Collaborative work*	*(…)-This is also something about the image (design) that we didn’t think about at the beginning. We think about it as we go along. For example, you didn’t see a need for this at first. I saw a different need, so now we’re combining the two into something that makes sense. This will also happen during development (…) (ICT Professional 1).-And that will make things easier, right?! (Lead Researcher). (…)-Going back to the other screens, I think we’ve finished addressing the design issue, right?!… (…). (ICT Professional 1).-Yes! (Lead Researcher).* *(…)* *--It’s a part of the patient’s progression, which I think is a more differentiated part. Here it will be like a timeline showing the days of the publications… (…) (ICT Professional 1).-Great, and that was the real meaning of the progression. But remember that here it can’t be a mix of progression from all patients, just from this specific patient (…) (Lead Researcher).*
***Development of the Psychosocial Rehab App with testing and feedback from the lead researcher***	***Code:*** *Development, testing, and feedback from the lead researcher*	*(…)-That’s it, that’s good, that’s cool, okay? That’s what waiting means. Do you have any questions? (Lead Researcher).-Not yet, but in a little while. It’s coming up, it’s coming up (ICT Professional 1). (…) (It’s necessary to) Test it too, I’ve already uploaded it (the Psychosocial Rehab App) to the cloud. So, you can come in here and test it. It’ll be better for us (ICT Professional 2). (…)-Cool, cool. I’ll go in, but there’s not much. We just need to align what’s (here)… (Lead Researcher). (…)-(You) can look around (ICT Professional 2).-I’ll look around… just send me the link and my login information on WhatsApp… (…) We’re fixing it. It looks okay… The idea was cool, it is perfect (Lead Researcher).*
	***Code:*** *Operational difficulty of making the Psychosocial Rehab App usable*	*(…)-I might not link the project because there might not be any registrations yet. However, I would link the patients (…) together with the healthcare professional (ICT Professional 1).-Okay, but here, for example, if no one has the patient’s initial contact information, who will enter the CPF first? (…), And then we might have a problem with not being able to register…. (Lead* *Researcher).-You can leave that there. To (…) do it later, right, maybe… (ICT Professional 2).*

Source: Prepared by the authors (2025).

## Data Availability

The data are in the manuscript.

## Supplementary Materials

The following supporting information can be downloaded at: https://www.mdpi.com/article/10.3390/nursrep15070228/s1, Supplementary Data S1: Prototype; Supplementary Data S2: Virtual Meetings; Supplementary Data S3: Instructional Design; Supplementary Data S4: Field Diary.

## Public Involvement Statement

No public involvement in any aspect of this research.

## Guidelines and Standards Statement

This manuscript was drafted in accordance with the Standards for Reporting Qualitative Research (SRQR) for qualitative research (O’Brien BC, Harris IB, Beckman TJ, Reed DA, Cook DA. Standards for reporting qualitative research: A synthesis of recommendations. Acad Med. 2014;89(9):1245–1251. doi:10.1097/ACM.0000000000000388) [70].
