# Supplementary material for: Development of the Psychosocial Rehabilitation Web Application (Psychosocial Rehab App)
Source: Nurs Rep. 2025 Jun 25;15(7):228. doi: 10.3390/nursrep15070228 (PMC12300239; doi:10.3390/nursrep15070228)
Supplement: Supplementary file 1 [file nursrep-15-00228-s001.zip › Supplementary Data 1 - Prototype.docx]

**
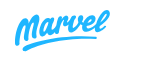
Supplementary Data 1 - Prototype**

**Screenshots of the second version of the interactive prototype for the web app “Psychosocial Rehabilitation Project App"**

**
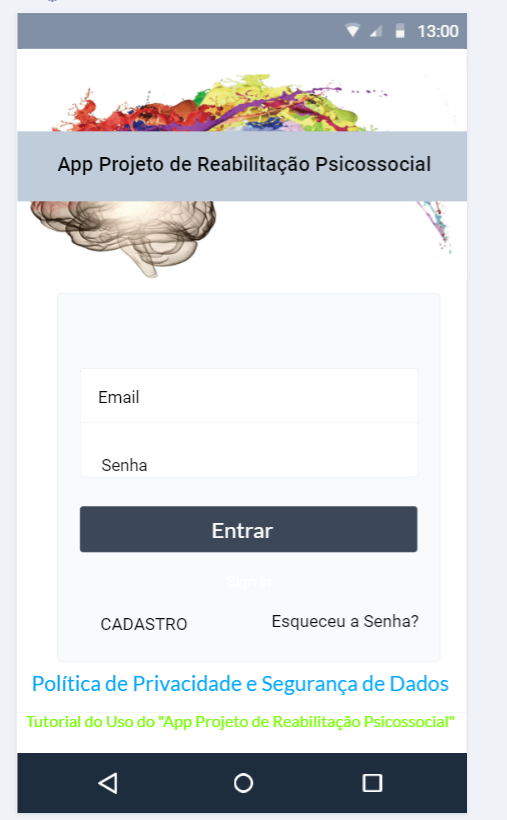
**

**Screens 1**

**
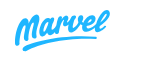

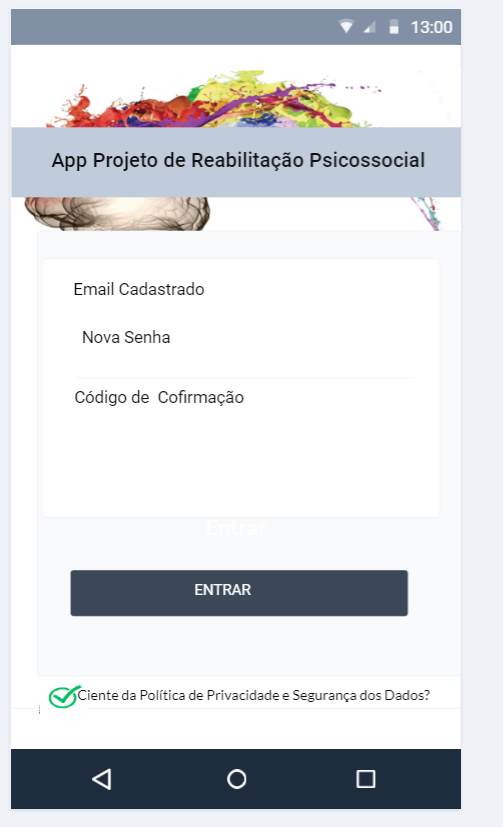
**

**Screens 2**

**
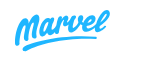

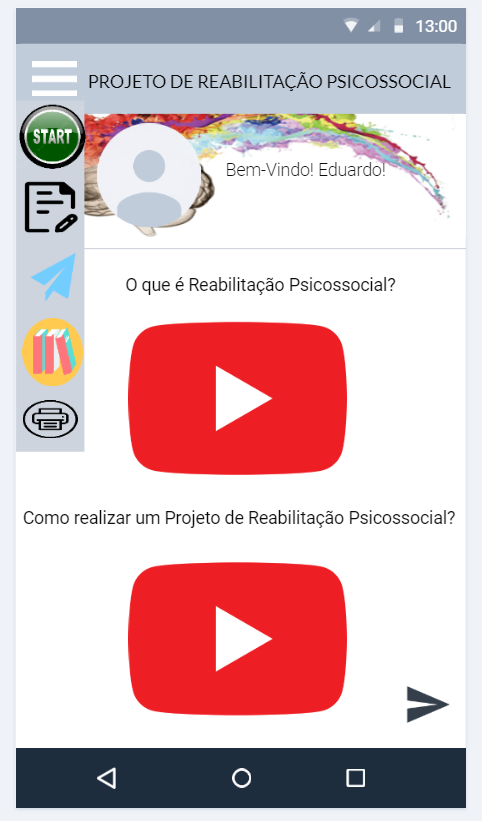
**

**Screens 3**

**
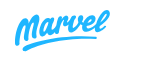
**
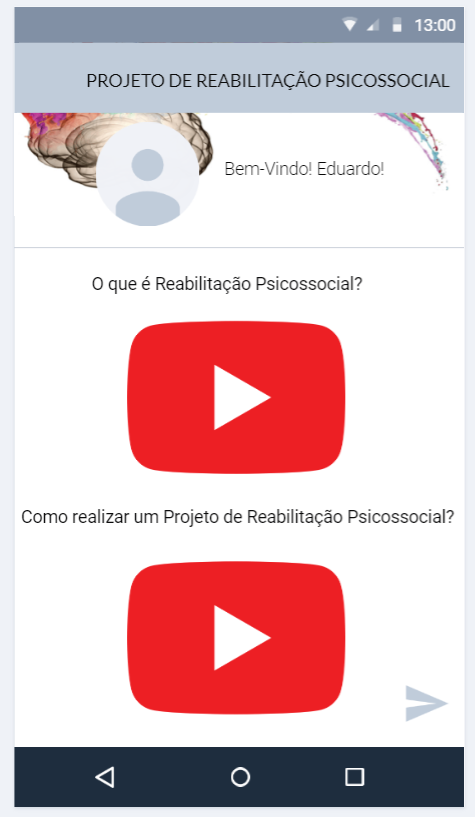


**Screens 4**

**
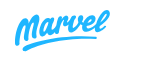

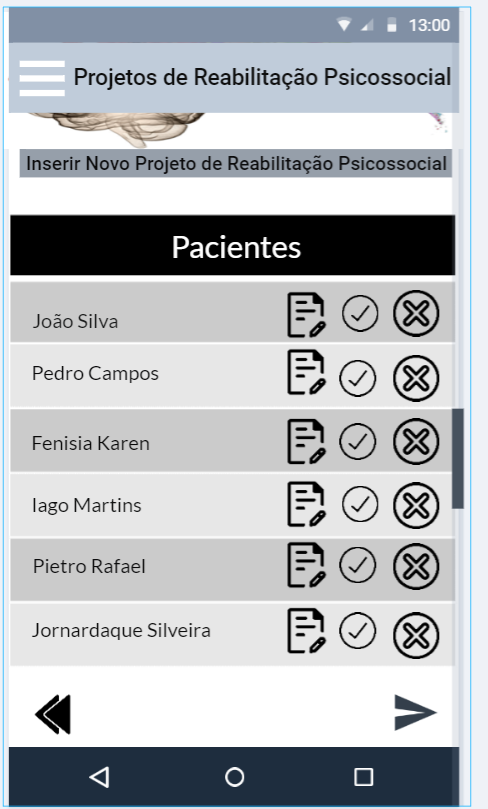
**

**Screens 5**

**
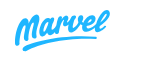

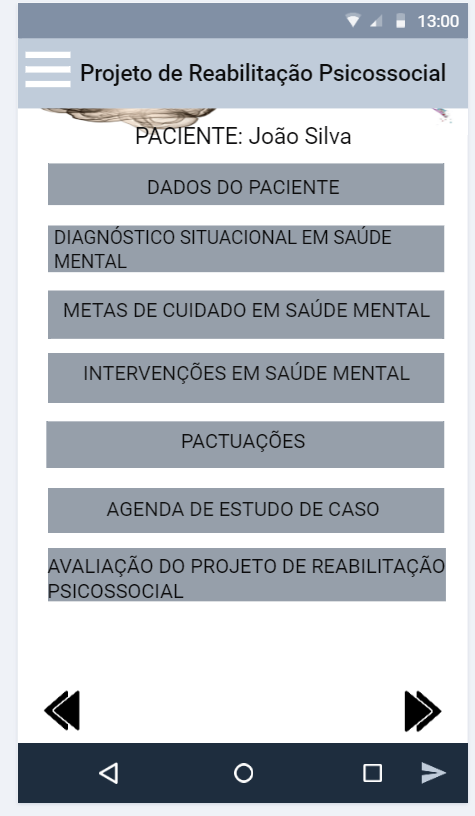
**

**Screens 6**

**
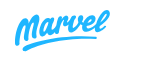

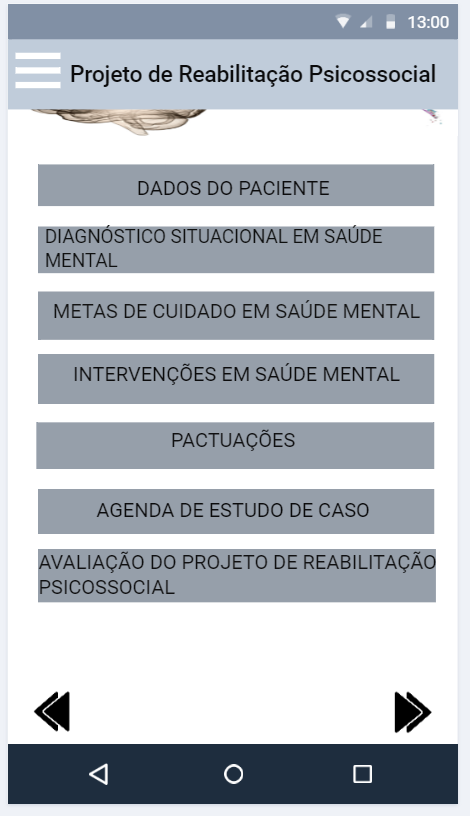
**

**Screens 7**

**
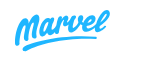

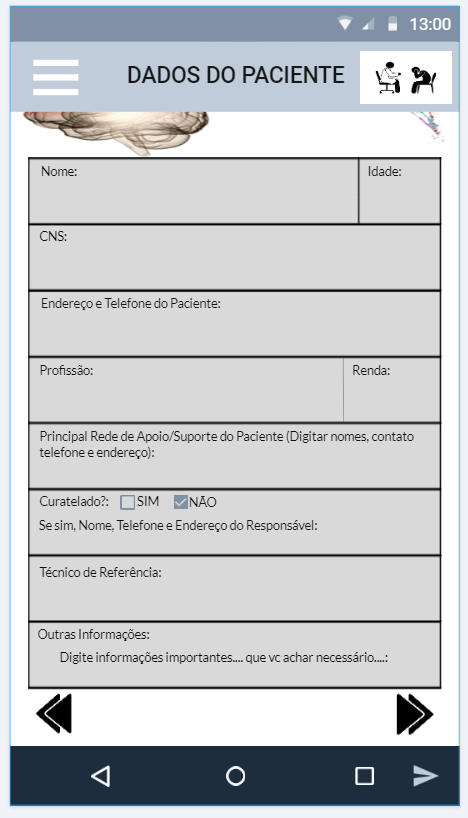
**

**Screens 8**

**
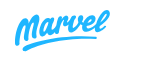

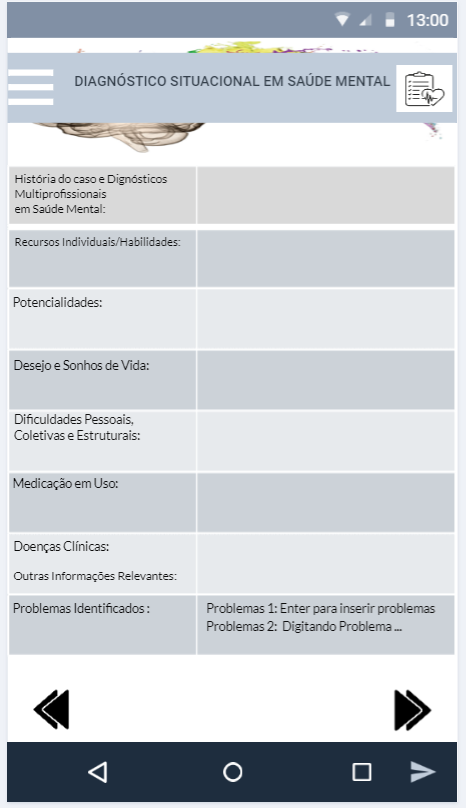
**

**Screens 9**

**
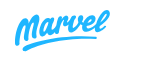

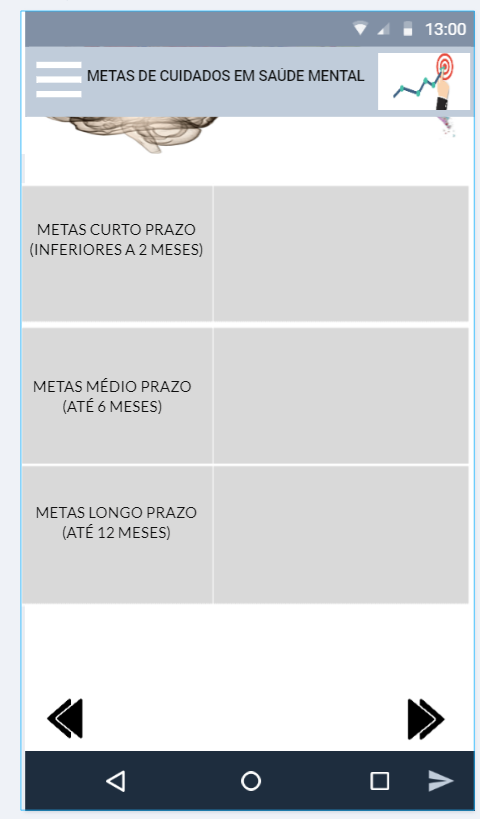
**

**Screens 10**

**
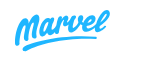

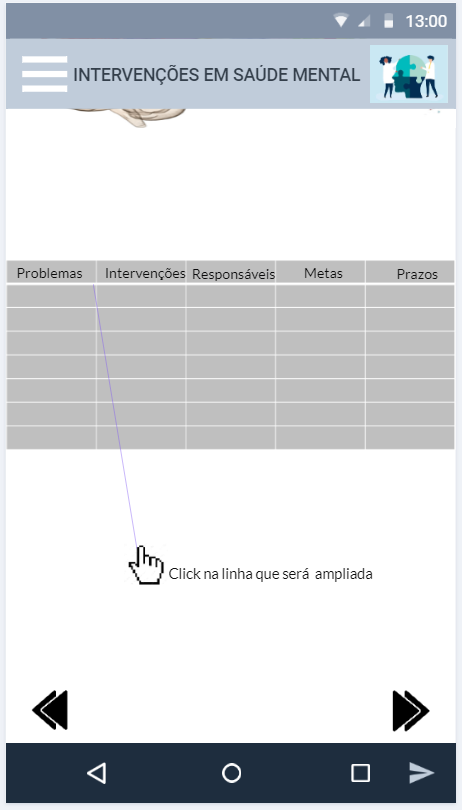
**

**Screens 11**

**
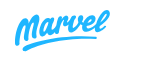

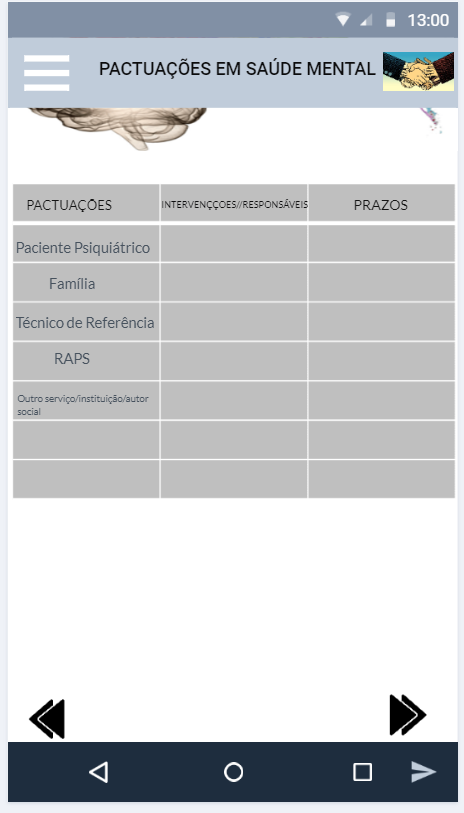
**

**Screens 12**

**
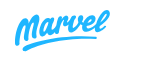

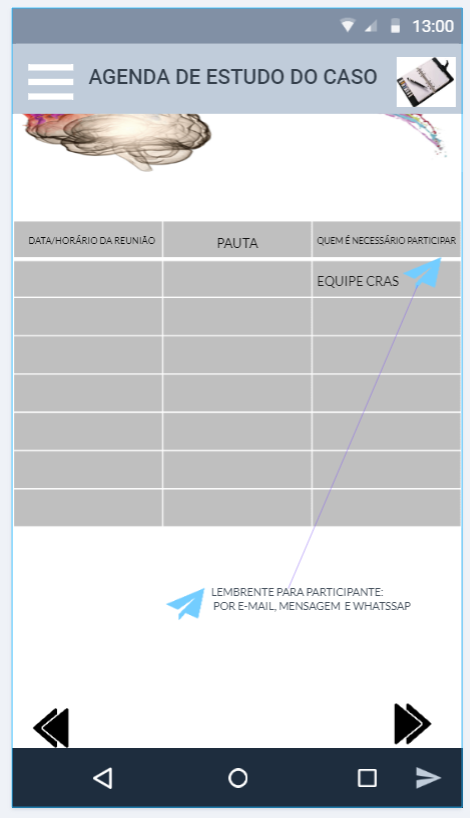
**

**Screens 13**

**
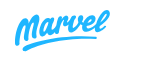

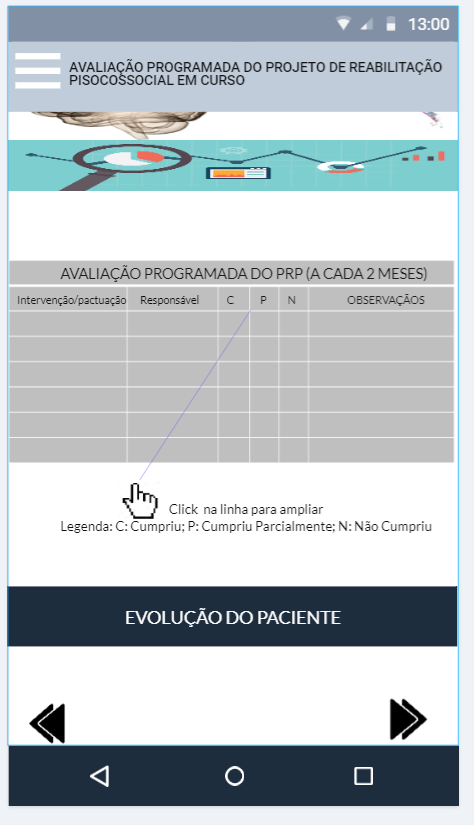
**

**Screens 14**

**
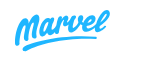

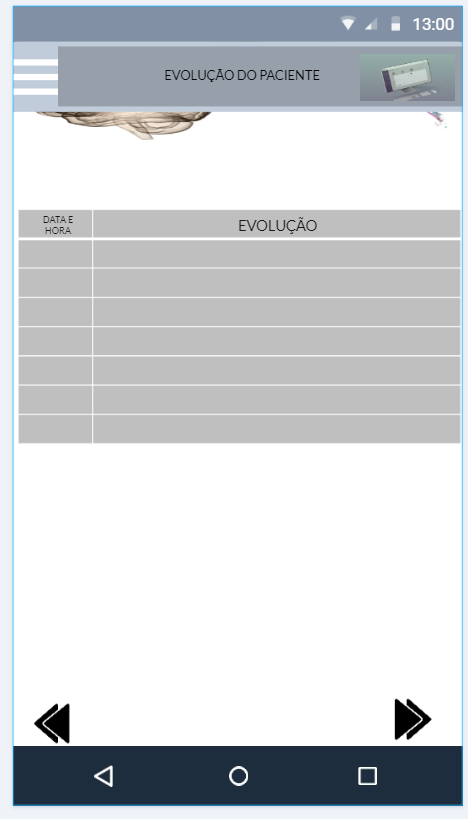
**

**Screens 15**

**
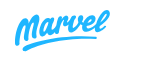

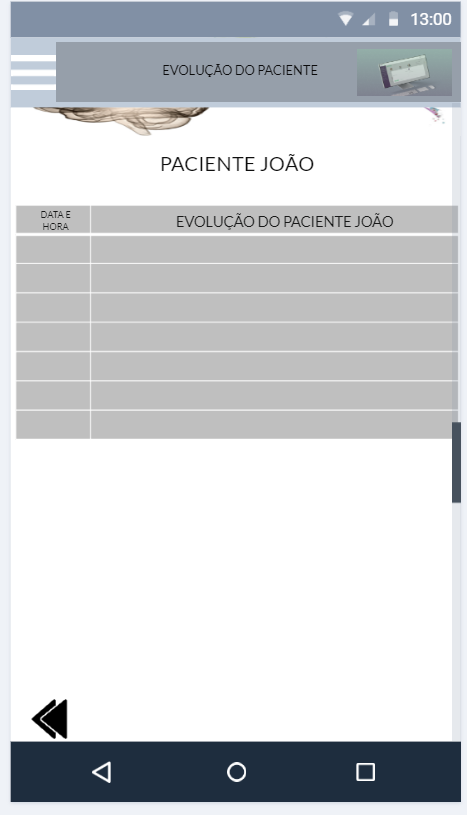
**

**Screens 16**

**
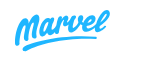

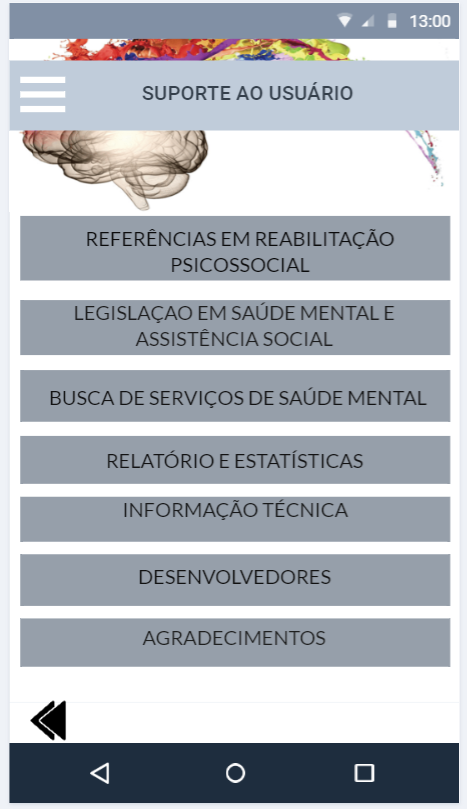
**

**Screens 17**

**
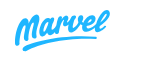

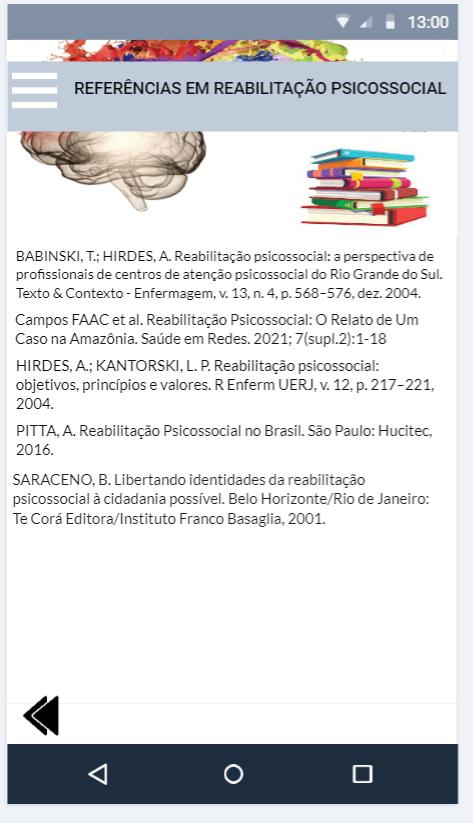
**

**Screens 18**

**
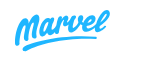

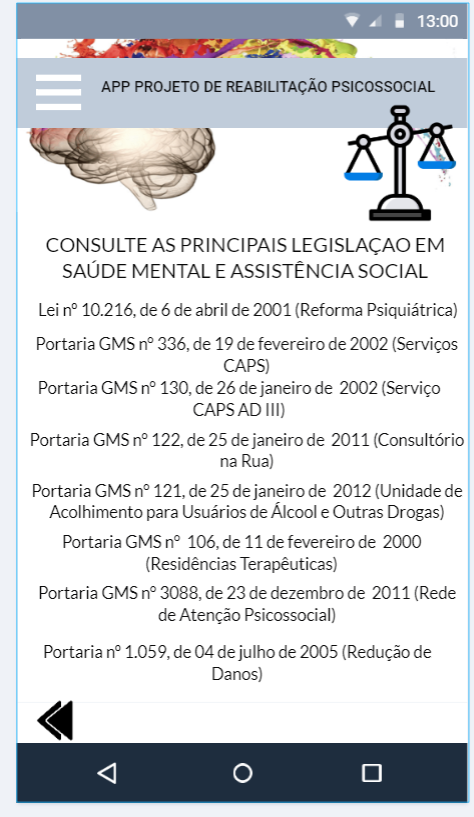
**

**Screens 19**

**
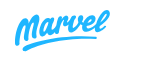

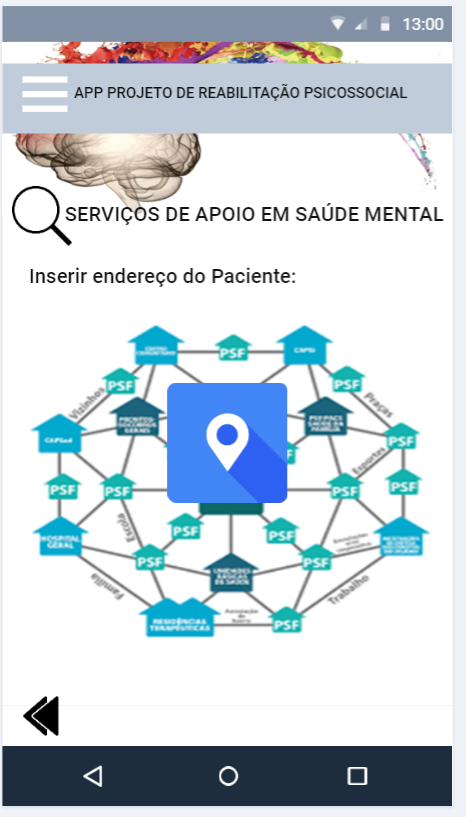
**

**Screens 20**

**
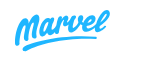

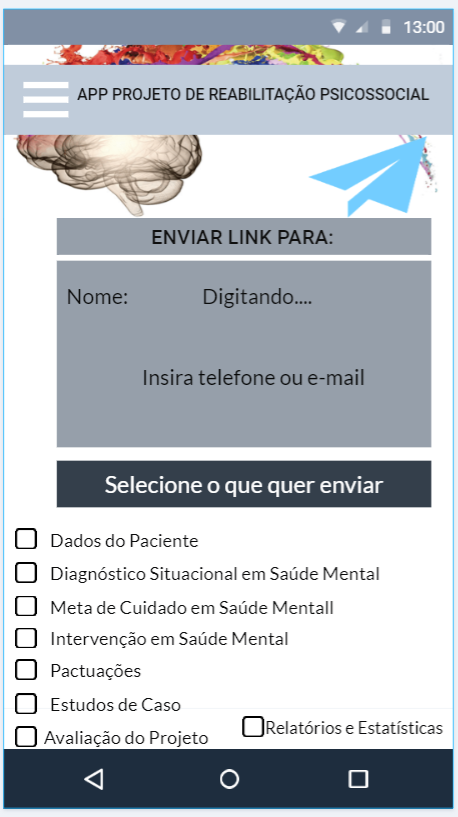
**

**Screens 21**

**
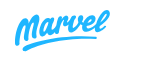

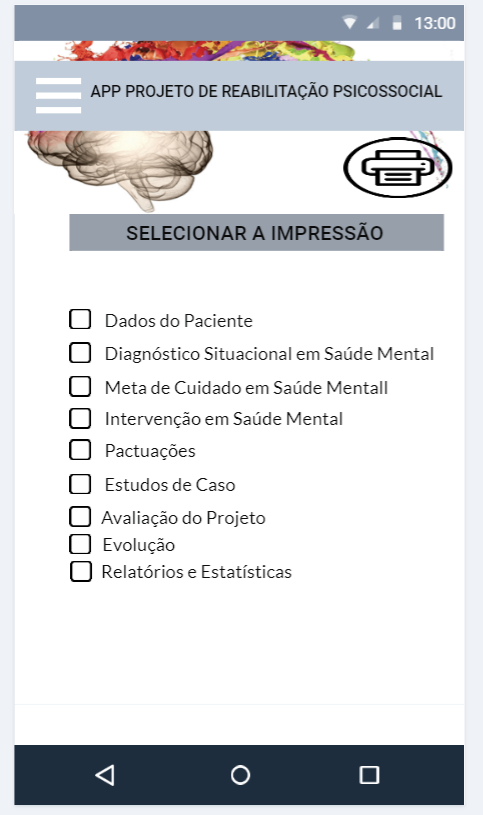
**

**Screens 22**

**
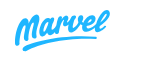

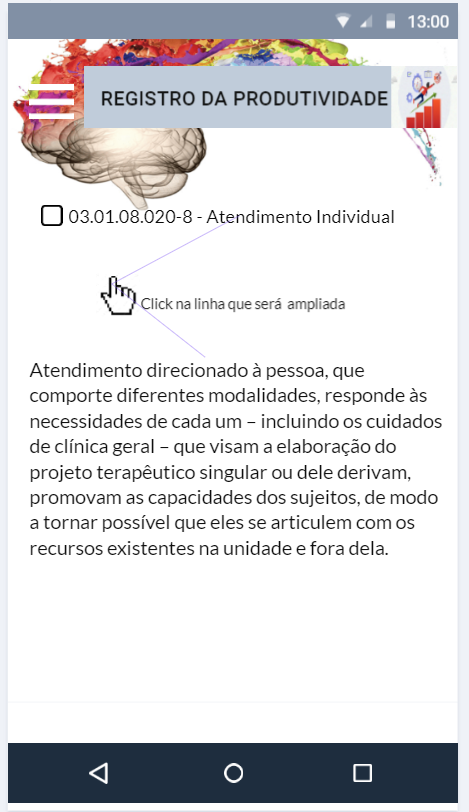
**

**Screens 23**

**
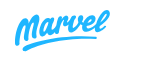

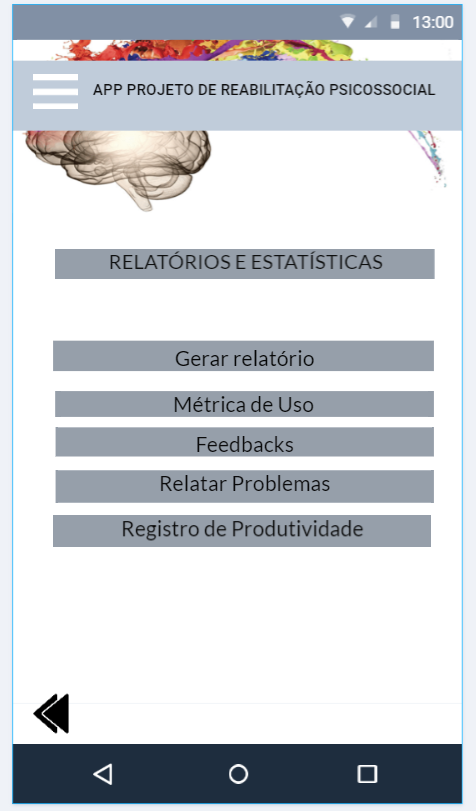
**

**Screens 24**

**
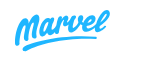

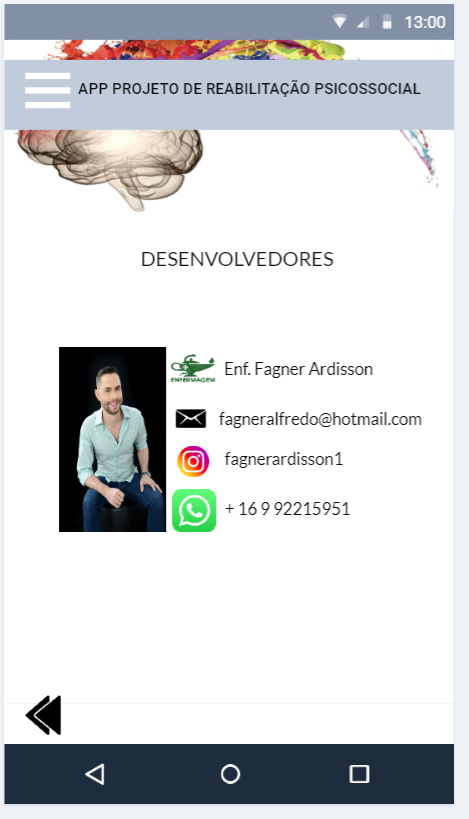
**

**Screens 25**

**
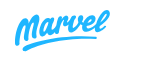

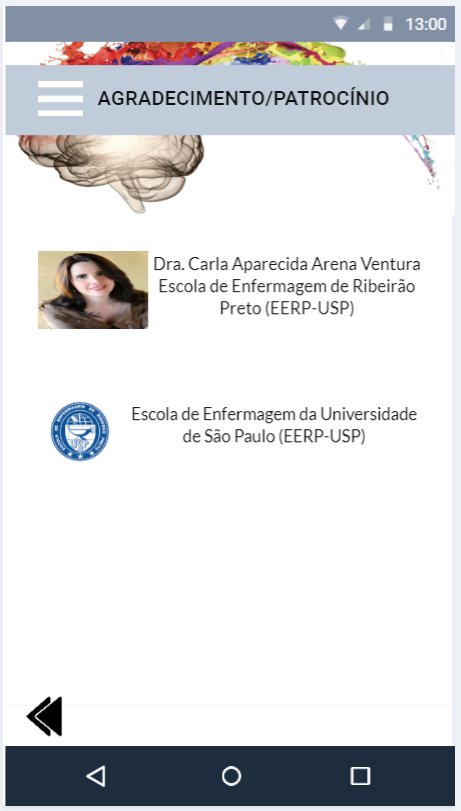
**

**Screens 26**

**
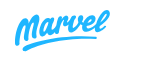

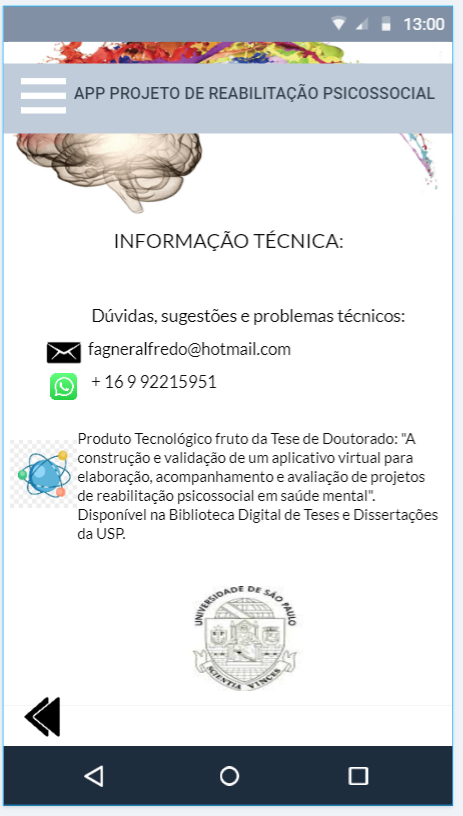
**

**Screens 27**
