# Supplementary material for: Development of the Psychosocial Rehabilitation Web Application (Psychosocial Rehab App)
Source: Nurs Rep. 2025 Jun 25;15(7):228. doi: 10.3390/nursrep15070228 (PMC12300239; doi:10.3390/nursrep15070228)
Supplement: Supplementary file 1 [file nursrep-15-00228-s001.zip › Supplementary Data 2 - Virtual Meetings.docx]

**Supplementary Data 2 - Virtual Meetings and Appointments**

**Presents virtual meetings and appointments for the development of the web app “Psychosocial Rehabilitation Project App” (English)**

| **Type/Date/Agenda** | **Participant(s)** | **Platform** | **Meeting/Appointment Summary** |
| --- | --- | --- | --- |
| First Meeting (05/14/2024): Connecting researchers with technology professionals. | Team of ICT professionals: made up of Developer 1, Developer 2, Technological Development Supervisor and the main researcher. | Google Meet | Research procedures are presented, the objective of which is to develop an app for psychosocial rehabilitation. The technological team proposes a web app for its accessibility and low cost. The importance of data security was emphasized, following the APA guides and recommendations by Martínez-Martín et al. (2021). |
| Second Meeting (22/05/2024) – Adjust and personalize the main page and the characteristics of the user profile. | Developer 1 and Principal Researcher | Google Meet | Aline the presentation of the home page and videos about the PRP (panel 5 of the PWAPPR), structure of the psychosocial rehabilitation project (panels 3, 4, 7, 8 to 14 of the PWAPPR). The characteristics of the user profile record (mental health professional) were discussed and constructed. |
| Unofficial conversation (26/05/2024): Need for additional clarifications on screen 7 (of the PWAPPR). | Developer 1 and Principal Researcher | WhatsApp | The main researcher explained to develop 1 that the rubrics on screen 7 describe the structure of a psychosocial rehabilitation project and which must be editable, easy to manage and cyclical. For its part, the Developer 1 suggested improvements to allow the user to insert screen captures, photos and edit them in a way that meets their needs during the construction of the psychosocial rehabilitation project. |
| Third Meeting (06/05/2024) – Presentation of the first graphic design to support the development of the web app application “Psychosocial Rehabilitation Project App”. | Developer 1, Developer 2 and Principal Researcher | Google Meet | Presentation of the initial instructional graphic design for the development of the web app “Psychosocial Rehabilitation Project App”. Discussing the patient's CPF will be a requirement for the first professional registration, without reaching a consensus. I propose as the main message on the home page: "Building life projects with meaning and meaning in home, social networks and work. Furthermore, it is suggested to include a solo explanatory video about the psychosocial rehabilitation project on the home page. Regarding the structure of the web app during the construction of the project, we proposed a resource to insert images and documents in the following sections: Patient Data, Situational Diagnosis in Mental Health, Agreement and Evaluation. As, the possibility of sharing and printing project items was created, allowing professionals to control what information is relevant and which is shared. It is agreed that the evolution of the project will be recorded in one line of time at a close and hourly rate, allowing all professionals who have access to the patient to record their actions. Screen 17 (User Support) will be included in the "User Support" submenu near the user profile. |
| Informal conversation (06/05/2024): Adjustment and adaptation of the first graphic design to support the development of the web app “Psychosocial Rehabilitation Project App”. | Developer 1 and Principal Researcher | WhatsApp | While registering the patient's CPF, the Principal Researcher suggested that this was used as an access control for the patient. I proposed to the developers that the CPF had not been registered in the user registry, but that they had been requested at the time of patient registration by the mental health professional, thus conditioning access to the rehabilitation project. It was planned to create an administrator profile to control access permissions to the web app “Psychosocial Rehabilitation Project App”. |
| Official conversations (07/02/2024, 07/03/2024 and 07/22/2024): Hiring of the animated video service for the main page of the web app “Psychosocial Rehabilitation Project App”. | Principal Researcher, Project Manager and Screenwriter | WhatsApp | Contact with a company developing animated videos to create a short video on the main topics and concepts of the psychosocial rehabilitation project, synthesized from a narrative review carried out by the researcher (20). |
| Fourth Meeting (07/12/2024): Need to adjust and adapt the first graphic design to support the development of the web app application “Psychosocial Rehabilitation Project App”. | Developer 1, Developer 2 and Principal Researcher | Google Meet | It was suggested to create an administrator user profile to validate the records of users of the web app “Psychosocial Rehabilitation Project App”. Please ask for the "User Support" section to include bibliographies, legislation and productivity records. Developers advised against the use of artificial intelligence due to its incipient state and doubts about its reliability in the management of mental health data. |
| Fifth Meeting (07/16/2024): Presentation of the final graphic design of the web app “Psychosocial Rehabilitation Project App” to start its development. | Developer 1, Developer 2 and Principal Researcher | Google Meet | Presentation of the advanced instructional graphic design for the development of the web app “Psychosocial Rehabilitation Project App” developed by technology professionals (see link: <https://www.figma.com/design/fav5XGcsRa0c0BrD4FQt9T/ReabilitaPSocial---Projeto-de-Reabilita%C3%A7%C3%A3o-Psicossocial?node-id=132-398&t=bqx25wi60EM9K6nz-0>). The function of adding a photo of the patient to your data/avatar has been included, as well as the option to share the content of the patient's project. Previously prototyped tables have been replaced by threads to improve readability. The medication field has been modified to include text fields for medication, quantity and frequency. In the intervention topic, the following group was added: habitat, social network and work. |
| Official conversations (19/08/2024 and 20/08/2024): Contracting of legal services for the elaboration of Terms and Conditions of Use of the web app “Psychosocial Rehabilitation Project App”. | Investigator and lawyer | WhatsApp | The lawyer agreed to elaborate the Terms and Conditions of Use of the web app “Psychosocial Rehabilitation Project App”, based on the information provided by the main researcher. It is agreed that adjustments and adaptations will be made to suit the project research context. |
| Official conversation (09/23/2024): Approval of Terms and Conditions of Use of the web app “Psychosocial Rehabilitation Project App”. | Investigator and lawyer | WhatsApp | The main researcher exhaustively reviews the terms and conditions of use of the web app “Psychosocial Rehabilitation Project App”, drawn up by the lawyer, and gives its approval. These terms will form part of the contract that will be signed between those responsible for the app and its future users. |
| Official meeting (10/10/2024): Approval of the final graphic design of the web app “Psychosocial Rehabilitation Project App”. | Developer 1, Developer 2 and Principal Researcher | Google Meet | The finalized instructional graphic design is presented, which will serve as a model for the development of the application. The developers explained that the final application will have improved functionalities and features. |
| Official conversation (27/11/2024): Approval of the animated video script for the main page of the web app “Psychosocial Rehabilitation Project App”. | Principal Researcher and Screenwriter | WhatsApp | The company sends the animated video script for your approval. The researcher suggested including an image of a consultation, the theme of rehabilitation theory and finalizing the video highlighting the web app. |
| Informal conversation (27 and 28/11/2024): The Developer 2 requested clarifications on the “agreements” heading and its deadlines. | Principal Researcher and Developer 2 | WhatsApp | The researcher clarified that the number of “Agreement” could be numerous and vary according to the needs of the professional, and could be carried out with the patient, other professionals and services. The evaluation of these “Agreements” will be carried out each month, as established in the psychosocial rehabilitation project. |
| Sixth Meeting (11/29/2024): Preliminary presentation of the web app “Psychosocial Rehabilitation Project App” to the main researcher. | Developer 1, Developer 2 and Principal Researcher | Google Meet | The developers presented the development of the web app on the Visual Studio Code platform, performing a demonstration of the user/administrator registration, the main screen (home) and the functionality of creating a “New Psychosocial Rehabilitation Project”, including the compilation of patient and project data. Furthermore, please note that the database is hosted on Firebase. |
| Official meeting (12/12/2024): Reception and approval of the animated video rolled out to the main page of the application “Psychosocial Rehabilitation Project App”. | Principal Researcher and Administrator | E-mail | The main researcher receives the animated video developed for the web app “App project for psychosocial rehabilitation” (available at: <https://drive.google.com/file/d/1D1hCciMdHDO41AXumbduZeXlwa0kvjS2/view?usp=sharing>  ), which has been approved for its coherence with the content and theoretical framework of the project. The video will be delivered to the developers for integration on the main page of the web app. |
| Meeting (12/19/2024) – Presentation of the web app “Psychosocial Rehabilitation Project App” with 90 percent development. | Developer 1, Developer 2 and Principal Researcher | Google Meet | The Developer 2 presents the access link to the web app application “Psychosocial Rehabilitation Project App”: [www.reabilitapsicossocial.com](http://www.reabilitapsicossocial.com) (hosted on the HostGator website), providing access credentials for professionals and administrators. The need to update the "User Support" section with updated information on psychosocial rehabilitation, legislation and procedures to record productivity was identified. |
| Meeting (01/13/2025; 01/14/2025; 01/15/2025; 01/24/2025): Final adjustments on the web app “Psychosocial Rehabilitation Project App”. | Developer 1, Developer 2 and Principal Researcher | Google Meet | The TIC 2 presented the improvements requested by the researcher in the previous meeting, including the correction of technical instabilities such as the duplication of projects and errors when changing screens, as well as small spelling and design corrections to guarantee the coherence and adequacy of the web app “Psychosocial Rehabilitation Project App” research project. Furthermore, we request that the improvements be implemented in a way that does not compromise the functionality of the system and that it adapts to version 2 of the prototype (PWAPPR), validated by mental health professionals. |
| Official Meeting (27/01/2025; 10/02/2025): Test and alpha evaluation with the researcher and ICT professionals developing the “Psychosocial Rehabilitation Project App”. | Developer 1, Developer 2 and Principal Researcher | Google Meet | Alpha test (pilot) of the web app “App project for psychosocial rehabilitation”, corrections of spelling errors, interface failures (screen duplication or absence of screens), before making it available for beta validation by mental health and technology professionals. |

**Source** : Do by the authors (2024).

**Apresenta as reuniões e interações virtuais para o desenvolvimento do webapp “App projeto de reabilitação psicossocial” (Português)**

| **Tipo/Data/Pauta** | **Participante(s)** | **Plataforma** | **Resumo da reunião/encontro** |
| --- | --- | --- | --- |
| Primeira Reunião (14/05/2024) – Conectando pesquisador e profissionais da tecnologia. | Profissionais de TIC (TIC 1, TIC 2 e TIC 3 e pesquisador) | Google Meet | Apresentados os procedimentos da presente pesquisa, incluindo o objetivo de desenvolver um app para construção de projetos de reabilitação psicossocial por profissionais de saúde mental. A equipe de tecnologia propôs a criação de um webapp, destacando sua acessibilidade e baixo custo. Como também foram ressaltadas as medidas de segurança necessárias para garantir a proteção das informações dos usuários (APA e recomendações de Martinez-Martin; Greely; Cho, 2021). |
| Segunda Reunião (22/05/2024) – Alinhamento da Home e características do perfil do usuário. | TIC 1 e pesquisador | Google Meet | Alinhamento quanto a apresentação da “Home” e vídeos sobre o PRP, estrutura do projeto de reabilitação psicossocial. E discutido e construído sobre/as característica do cadastro do perfil do usuário (profissional de saúde mental). |
| Conversa extraoficial (26/05/2024) – Necessidade de mais esclarecimento sobre a tela 7 (protótipo – versão 2). | TIC 1 e pesquisador | WhatsApp | O pesquisador esclareceu ao TIC 1 que os tópicos da tela 7 (versão 2 prototipada pelo pesquisador), descrevem a estrutura de um projeto de reabilitação psicossocial, e que eles devem ser editáveis, de fácil manuseio e cíclicos. Por sua vez o TIC 1, sugeriu o aprimoramento dela, de modo ao usuário poder colocar prints, fotos e editar de forma que atendesse as suas necessidades durante a construção do projeto de reabilitação psicossocial. |
| Terceira Reunião (05/06/2024) – Apresentação do primeiro design gráfico para subsidiar o desenvolvimento do webapp “App projeto de reabilitação psicossocial”. | TIC 1, TIC 2 e pesquisador | Google Meet | Apresentação do design gráfico instrucional (inicial) para o desenvolvimento do webapp “App projeto de reabilitação psicossocial”. Discutido sobre se o CPF do paciente será requisito para realizar o primeiro cadastro do profissional, porém não foi chegado em consenso. Em relação ao conteúdo da mensagem na Home foi proposta a frase: “Construindo projetos de vida com sentidos e significados no habitat, rede social e trabalho”. Além de ser deixar na Home apenas 1 vídeo explicativo sobre projeto de reabilitação psicossocial. Em relação a estrutura do webapp durante a construção do projeto de reabilitação psicossocial foi proposto recurso para inserir imagens/documentos/atas/laudos/declarações etc, nos tópicos, se pertinente (Dados do Paciente, Diagnóstico Situacional em Saúde Mental, Pactuações, Agenda e Avaliação). Além do mais os desenvolvedores trouxeram o recurso de compartilhar e imprimir os tópicos do projeto conforme a necessidade do profissional e controle do que é importante (as partes do projeto de reabilitação psicossocial) e a quem receberá. A evolução será construída em timeline com data e hora e registro cronológico, onde todos os profissionais que tiveram acesso para o paciente poderão registrar. A tela 17 (Suporte ao usuário - versão 2 prototipada pelo pesquisador), será inserida no submenu “Suporte ao Usuário” perto do perfil usuário. |
| Conversa extraoficial (05/06/2024) – Alinhamento e adequação do primeiro design gráfico para subsidiar o desenvolvimento do webapp “App projeto de reabilitação psicossocial”. | TIC 1 e pesquisador | WhatsApp | Em relação ao cadastro do CPF do paciente, o pesquisador principal sugeriu dele ser o controle de quem vai ter acesso àquele paciente. E sugerido aos desenvolvedores que o CPF não fosse cadastrado no cadastro do usuário, e sim, quando o profissional de saúde mental for cadastrar o paciente, sendo um condicionante de acesso ao projeto de reabilitação desse paciente. Foi pensado o modo administrador para ser ter controle sobre a permissão de quem vai usar o webapp “App projeto de reabilitação psicossocial”. |
| Conversas oficiais (02/07/2024, 03/07/2024 e 22/07/2024) - Contratação de serviço de Vídeo Animado para Home do webapp “App projeto de reabilitação psicossocial” | Pesquisador e Administrador e Roteirista | WhatsApp | Contato com empresa que desenvolve vídeo animado para fazer vídeo curto sobre principais tópicos e conceitos do projeto de reabilitação psicossocial, que foram sintetizados da revisão de literatura (capítulo 2). |
| Quarta Reunião (12/07/2024) – Necessidade de alinhamento e adequação do primeiro design gráfico para subsidiar o desenvolvimento do webapp “App projeto de reabilitação psicossocial”. | TIC 1, TIC 2 e pesquisador | Google Meet | Sugerido a criação de perfil de usuário administrador para validar os cadastros de usuário do webapp “App projeto de reabilitação psicossocial”. Solicitado que o recurso “Suporte ao usuário”, contenha as referências, legislação e registro de produtividade. Desenvolvedores desaconselham uso da inteligência artificial por esta ainda ser incipiente e não termos segurança no seu manuseio e fidedignidade ao ser usado em dados de saúde/mental. |
| Quinta Reunião (16/07/2024) - Apresentação do design gráfico do webapp “App projeto de reabilitação psicossocial” finalizado para começar o seu desenvolvimento. | TIC 1, TIC 2 e pesquisador | Google Meet | Apresentação do design gráfico instrucional de desenvolvimento do web “App projeto de reabilitação psicossocial” construídos pelos desenvolvedores, em processo bem avançado (ver link: <https://www.figma.com/design/fav5XGcsRa0c0BrD4FQt9T/ReabilitaPSocial---Projeto-de-Reabilita%C3%A7%C3%A3o-Psicossocial?node-id=132-398&t=bqx25wi60EM9K6nz-0>). Inserido recurso de foto do paciente em seus dados/avatar, recurso de compartilhar conteúdo do projeto do paciente, foi trocado as tabelas anteriormente prototipado por tópicos por serem mais legíveis, mudança no item medicação inserido via, medicação, quantidade, frequência em caixas de textos. No tópico intervenção inserido grupo: habitat, rede social e trabalho. |
| Conversa Oficial (19/8/2024 e 20/08/2024) – Contratação de serviços advocatícios para construção do Termo e Condições de Uso do webapp “App projeto de reabilitação psicossocial”. | Pesquisador e advogada | WhatsApp | Advogada aceita construir o termo e condições de uso do webapp “App projeto de reabilitação psicossocial (para seus usuários), mediante informações sobre este app fornecido pelo pesquisador. Sendo feitas devolutivas de melhoramento e adaptação para o contexto de pesquisa em que o app está envolto. |
| Conversa Oficial (23/09/2024) – Aprovação do Termo e condição de Uso do webapp “App Projeto de reabilitação psicossocial”. | Pesquisador e advogada | WhatsApp | Pesquisador confere minuciosamente o termo e condições de uso do webapp “App projeto de reabilitação psicossocial construído pela advogada e o aprova, de forma que este termo faça parte do contrato firmado entre os responsáveis do app e os seus usuários (futuros). |
| Reunião Oficial (10/10/2024) – Aprovação do design gráfico do webapp “App projeto de reabilitação psicossocial” | TIC 1, TIC 2 e pesquisador | Google Meet | Desenvolvedores apresentam design gráfico instrucional de desenvolvimento finalizado, explicou que esse design será o “espelho” do app, porém de forma aprimorada com funcionalidades e recursos que será programado por eles. |
| Conversa oficial (27/11/2024) – Aprovação do Roteiro do Vídeo Animado sobre projeto de reabilitação psicossocial para compor a Home do webapp “App projeto de reabilitação psicossocial”. | Pesquisador e roteirista | WhatsApp | A empresa contratada envia o roteiro para aprovação, pelo pesquisador, para prosseguir para a construção do vídeo animado para home do webapp “App projeto de reabilitação psicossocial”. Este roteiro foi modificado pelo pesquisador, com recomendações de inserir imagem de consultório em saúde mental, incorporação do tópico “Teoria da Reabilitação psicossocial” e finalização do vídeo com chamado para a atenção do profissional de saúde mental para o webapp “App Projeto de reabilitação psicossocial”. |
| Conversa extraoficial (27 e 28/11/2024) – TIC 2 solicita esclarecimento sobre o tópico pactuação e seus prazos. | Pesquisador e TIC 2 | WhatsApp | O pesquisador esclareceu que o tópico de pactuação podem ser inúmeras e dependendo da necessidade do profissional, sendo feita com paciente, outros profissionais e serviços, e sua avaliação foi definida no projeto de reabilitação psicossocial a cada 2 meses. |
| Sexta Reunião (29/11/2024) - Apresentação prévia do webapp “App projeto de reabilitação psicossocial” ao pesquisador. | TIC 1, TIC 2 e pesquisador | Google Meet | Os desenvolvedores apresentaram o webapp em desenvolvimento na plataforma Visual Studio Code, simulando o cadastro de usuário/administrador, demostrando a tela principal (home) e funcionalidade de inserir um “Novo Projeto de Reabilitação Psicossocial” com coleta de dados do paciente e dados do projeto de reabilitação psicossocial. Além disso foi dito que o banco de dado de dados é arquivado no Firebase. |
| Conversa oficial (12/12/2024) – Recebimento e aprovação do vídeo animado construído sobre PRP a ser integrado a Home do webapp “App projeto de reabilitação psicossocial”. | Pesquisador e Administrador | E-mail | Pesquisador recebe o vídeo animado construído sobre PRP a ser integrado a Home do webapp “App projeto de reabilitação psicossocial”, (que pode ser consultado no link: <https://drive.google.com/file/d/1D1hCciMdHDO41AXumbduZeXlwa0kvjS2/view?usp=sharing>), sendo aprovado pelo pesquisador por ser condizente ao conteúdo e referencial teórico do PRP, e encaminhado ao desenvolvedores para se inseri-lo na Home deste app em desenvolvimento. |
| Reunião Oficial (19/12/2024) – Apresentação do webapp “App projeto de reabilitação” com 90 porcento desenvolvimento. | TIC 1, TIC 2 e pesquisador | Google Meet | O TIC 2 apresentou o link que se pode acessar o webapp “App projeto de reabilitação psicossocial”: [www.reabilitapsicossocial.com](http://www.reabilitapsicossocial.com) (hospedado no sítio do HostGator), sendo lhe passado as senhas de Acesso de professional e administrador. Foi percebido a necessidade de atualizar (Suporte ao Usuário) as referências sobre reabilitação psicossocial, legislações e os procedimentos de registro da produtividade no âmbito da saúde mental. Como também, se liberou o acesso ao webapp “App projeto de reabilitação psicossocial” mediante o login/senha de profissional de saúde mental para o pesquisador visualizar e testar este app em fase de finalização. |
| Reunião Oficial (13/01/2025; 14/01/2025; 15/01/2025; 24/01/2025): Ajustes finais no webapp “App projeto de reabilitação psicossocial”. | TIC 1, TIC 2 e pesquisador | Google Meet | O TIC 2 apresentou as mudanças de aperfeiçoamento solicitadas pelo pesquisador em reunião anterior, juntamente, o pesquisador solicitou ajuste em relação as instabilidades tecnológicas como duplicação de projetos, “quebras” em mudanças de telas, pequenas correções ortográficas em relação ao design e aparência de forma deixar o webapp “App projeto de reabilitação psicossocial” coerente e adequado ao projeto de reabilitação psicossocial, e na medida que possível, desde que não comprometa sua funcionalidade tecnológica, personalizado conforme a versão 2, prototipada e valida por profissionais de saúde mental, como apresentada na etapa 1 da presente pesquisa apresentada nessa tese. |
| Encontro Oficial (27/01/2025; 10/02/2025): Testagem e validação alfa com o pesquisador e profissionais de TIC desenvolvedores do “App projeto de reabilitação psicossocial”. | TIC 1, TIC 2 e pesquisador | Google Meet | Testagem alfa (piloto) do webapp “App projeto de reabilitação psicossocial”, correções de erros de ortografia, falhas de interface (duplicação de tela ou ausência de telas), antes de disponibilizá-lo a validação beta por profissionais de saude mental e tecnologia. |

**Fonte**: Elaborado pelo autor (2024).
