# Supplementary material for: Development of the Psychosocial Rehabilitation Web Application (Psychosocial Rehab App)
Source: Nurs Rep. 2025 Jun 25;15(7):228. doi: 10.3390/nursrep15070228 (PMC12300239; doi:10.3390/nursrep15070228)
Supplement: Supplementary file 1 [file nursrep-15-00228-s001.zip › Supplementary Data 3 - Instructional Design.docx]

Supplementary Data 3 - Instructional Design for Development of the webapp App for psychosocial rehabilitation project


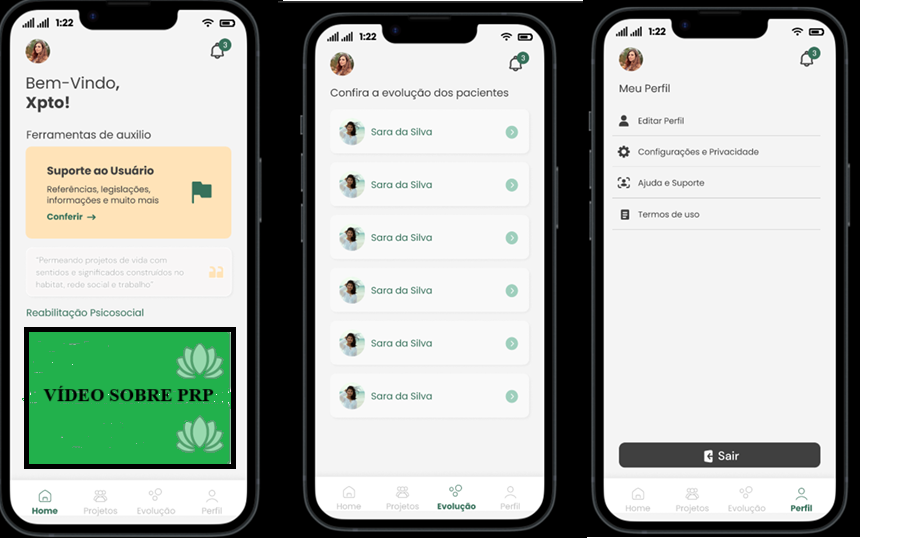


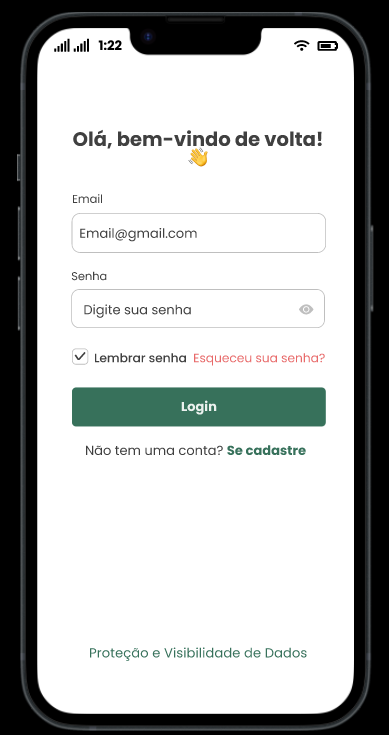

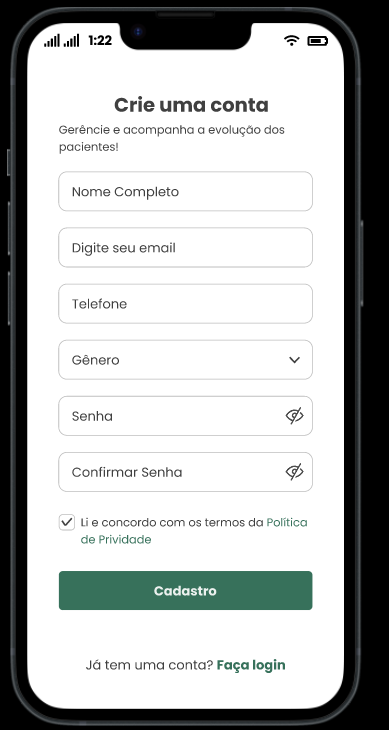

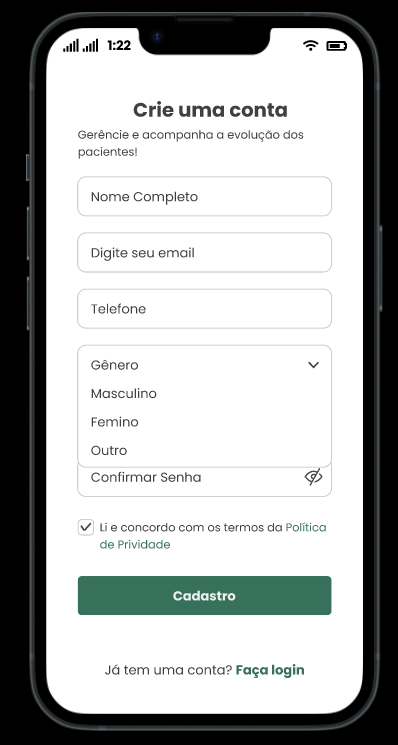

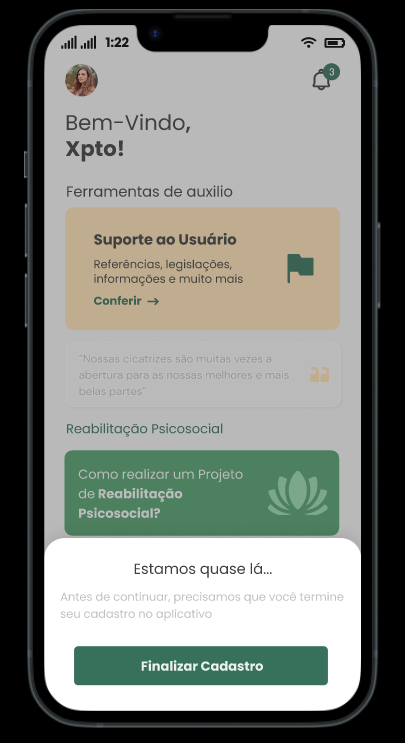

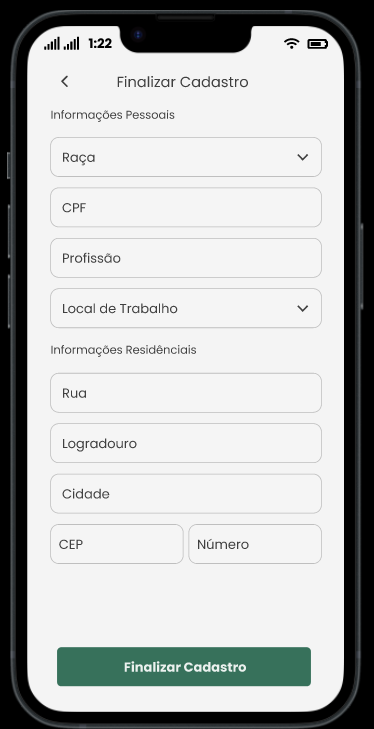

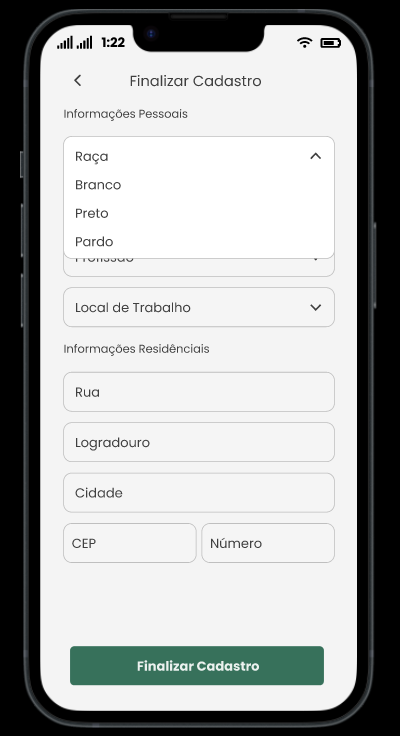

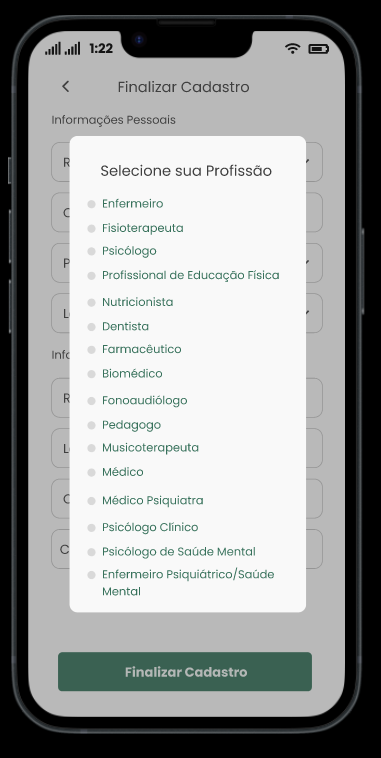

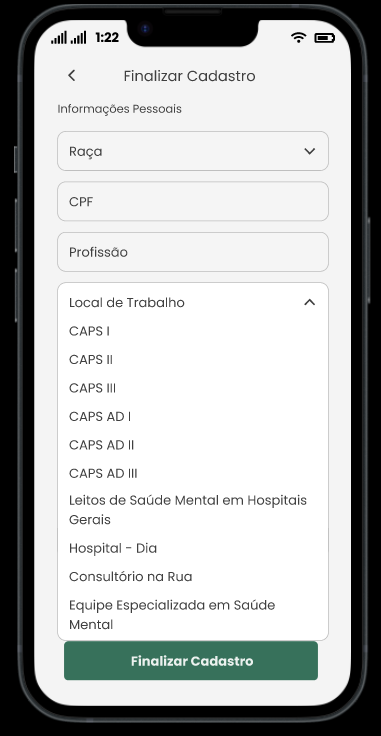

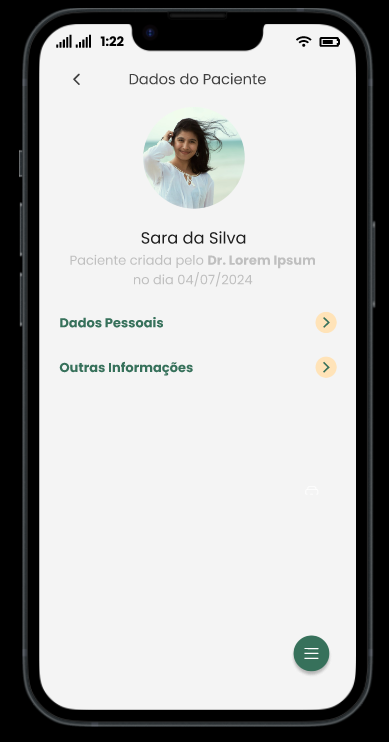


**Continua na próxima página...**


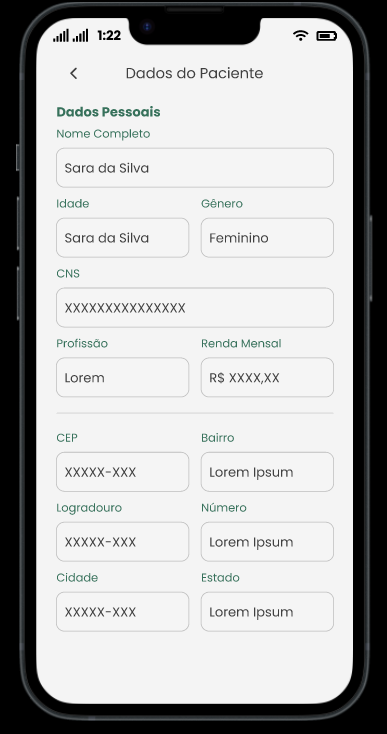

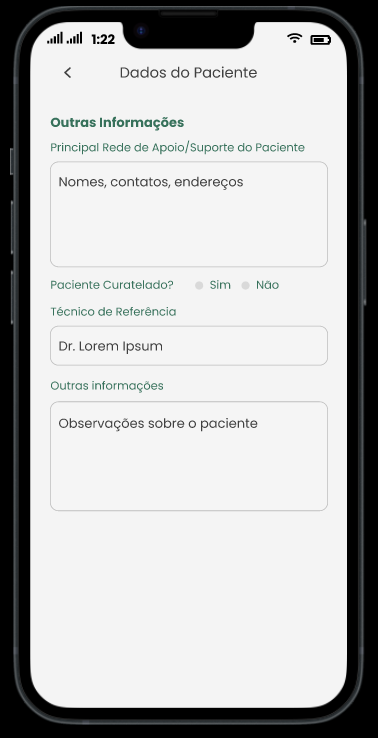

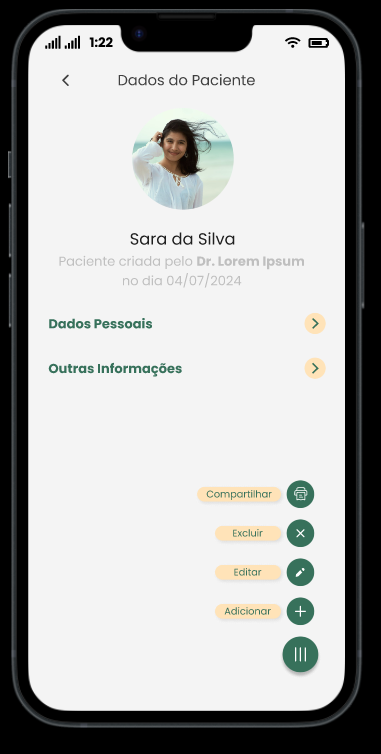

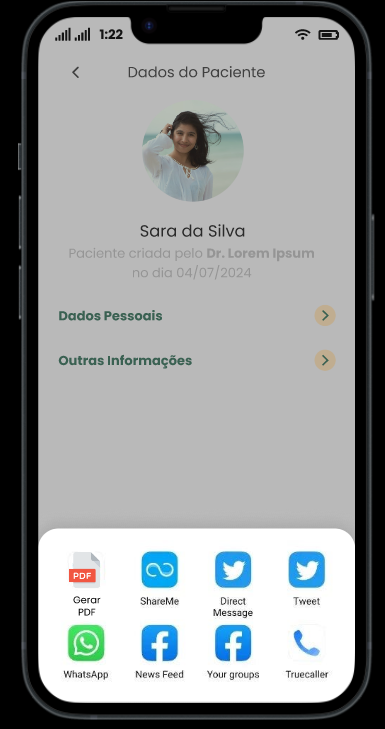

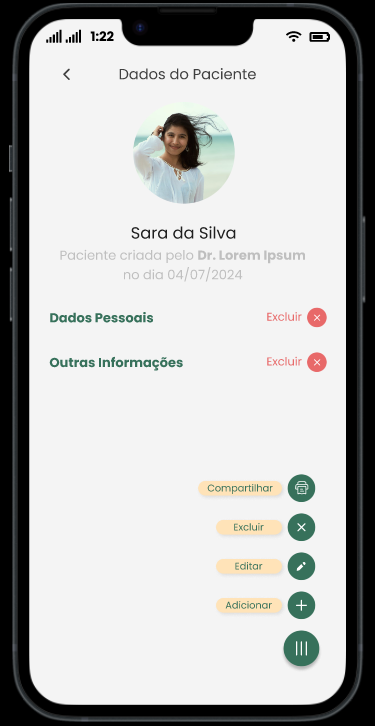

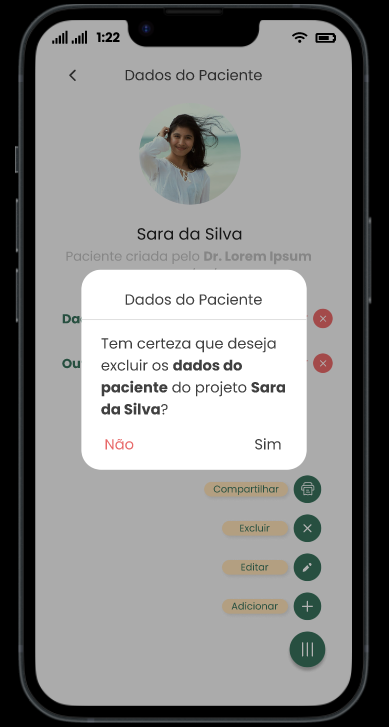

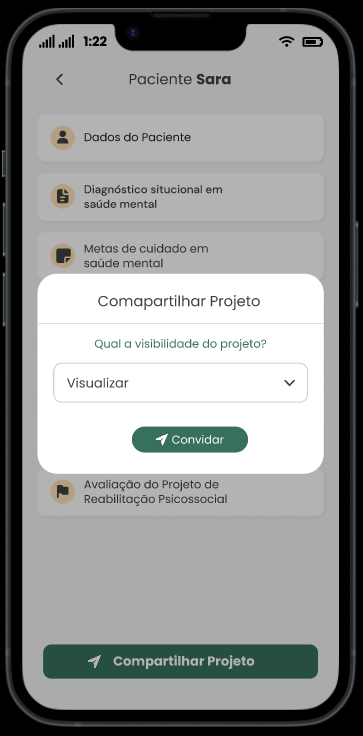

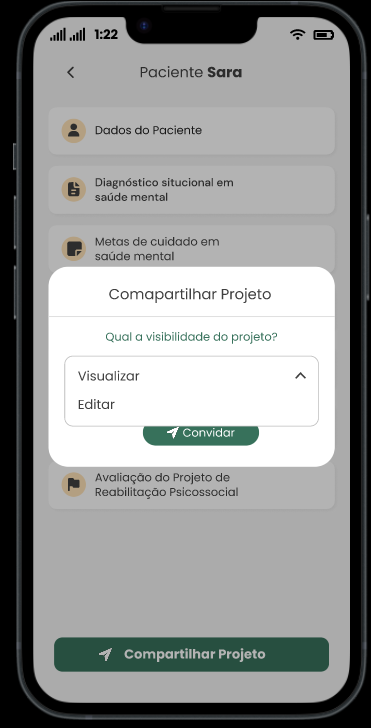

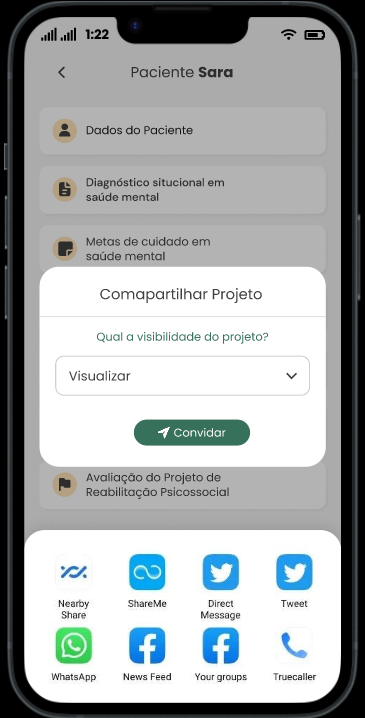


**Continua na próxima página...**


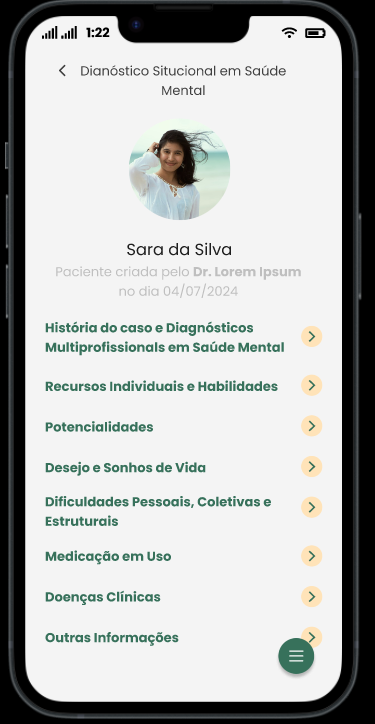

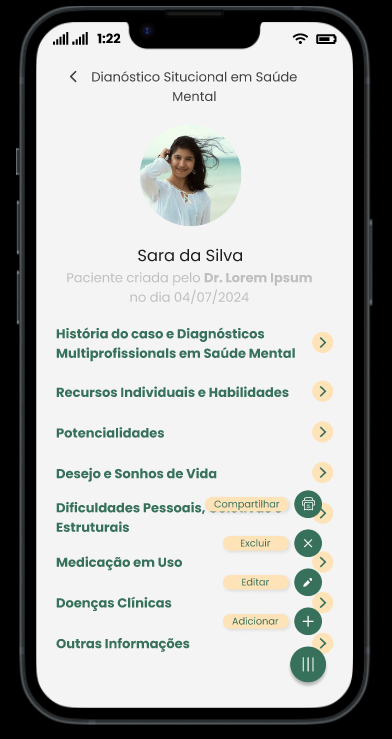

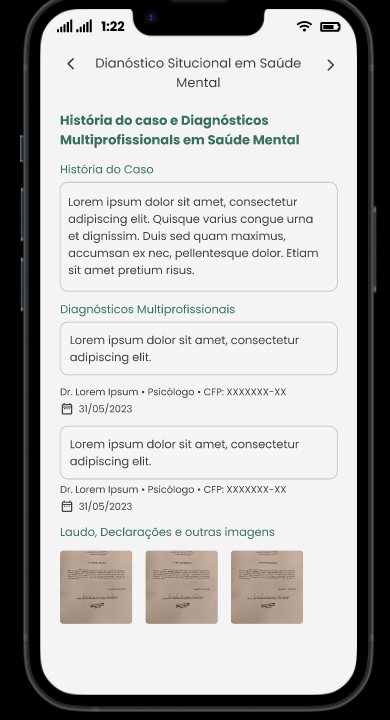


**Continua na próxima página...**


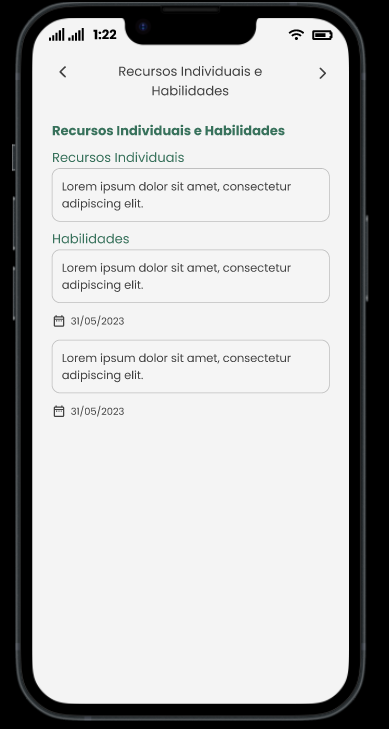

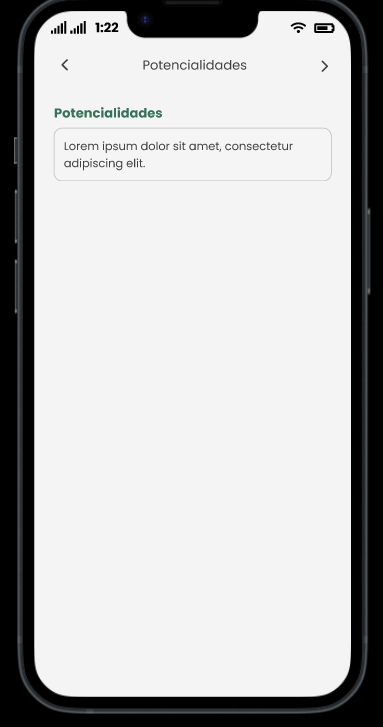

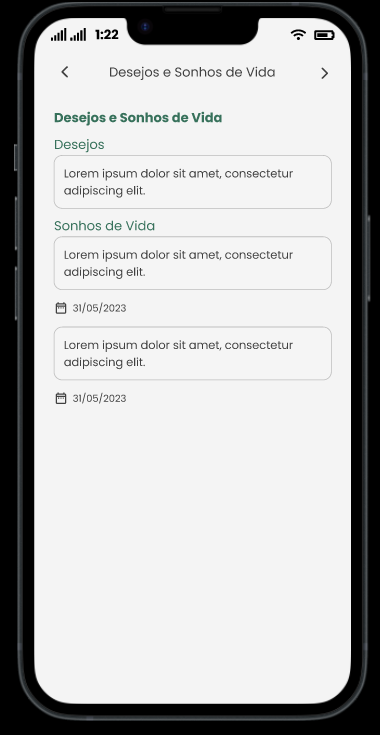


**Continua na próxima página...**


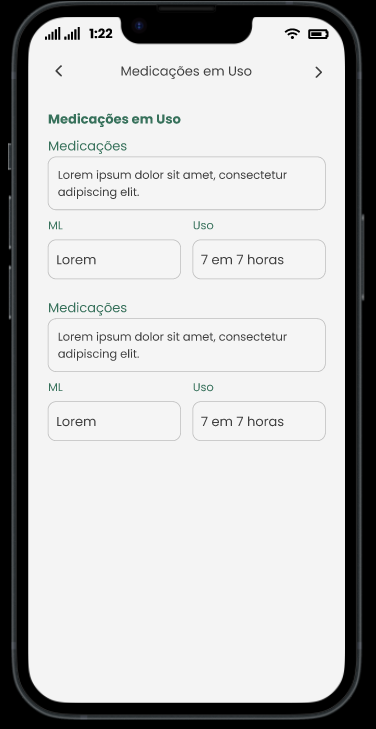

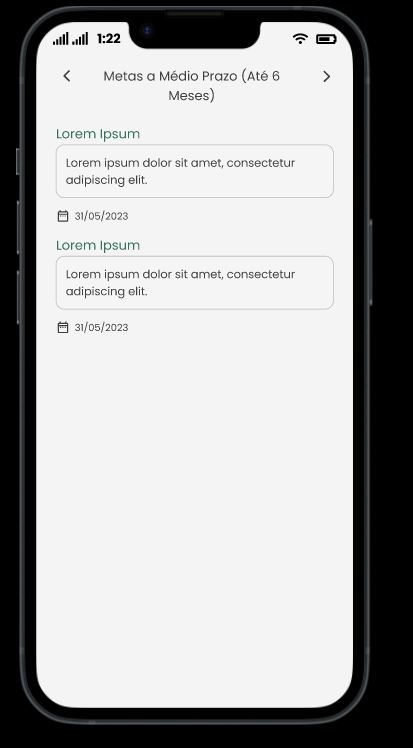

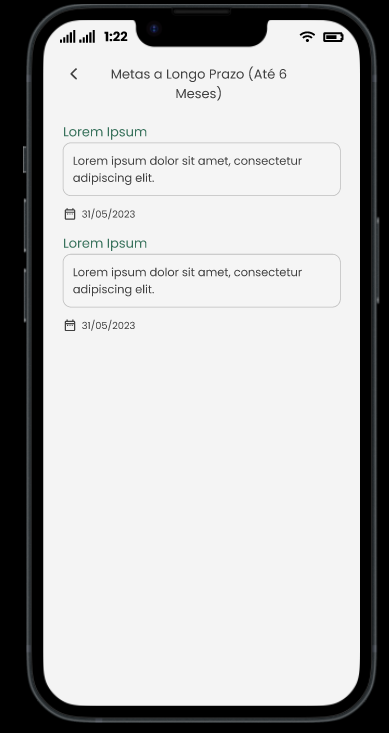

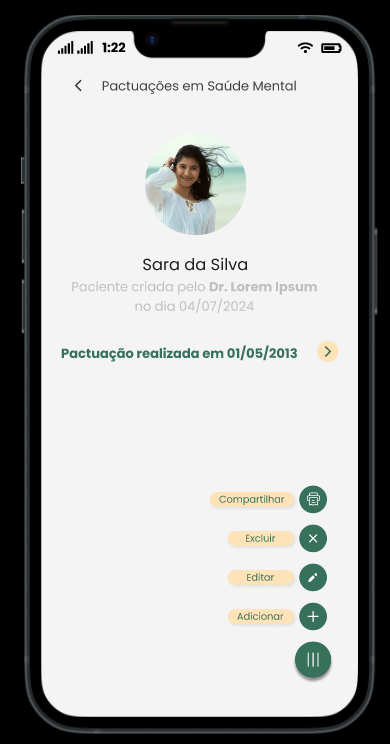

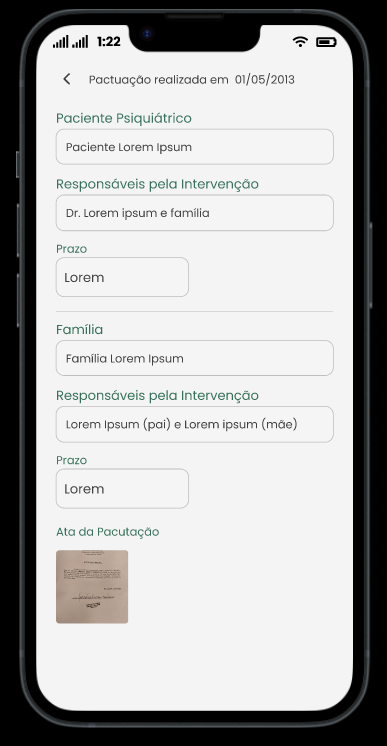

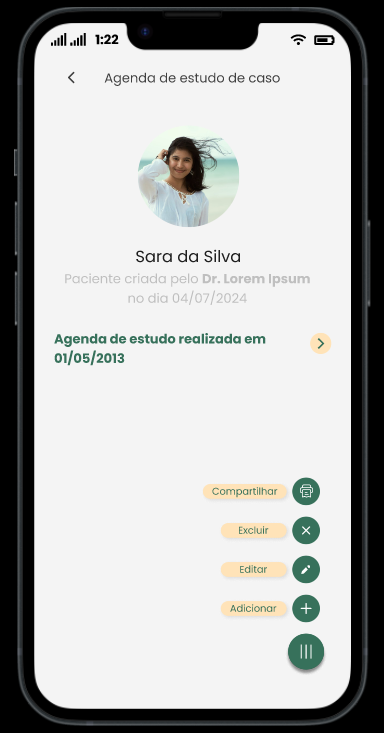

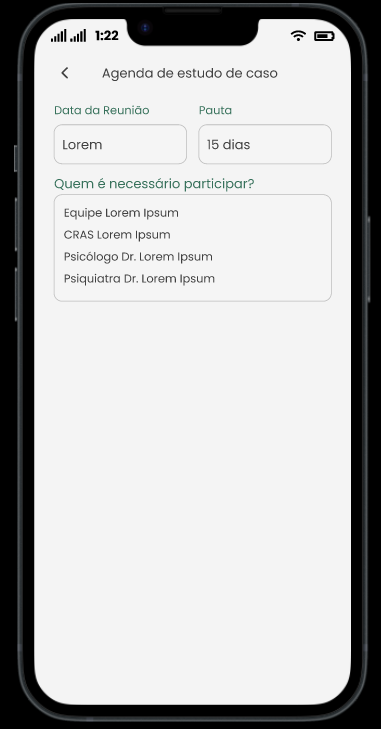

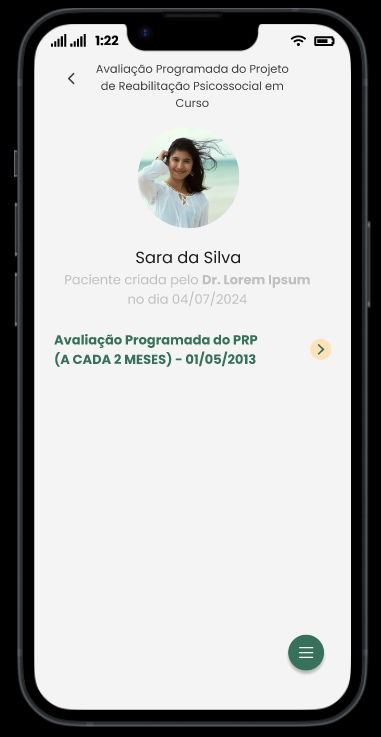

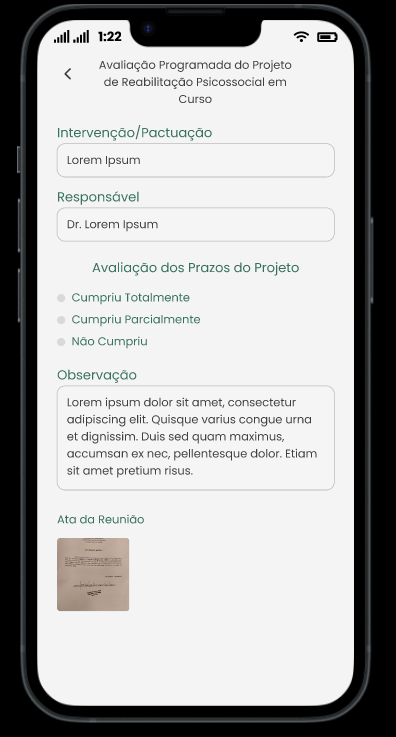

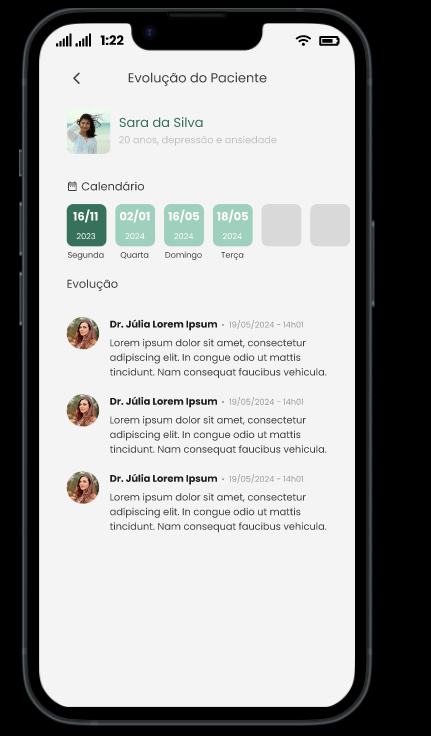

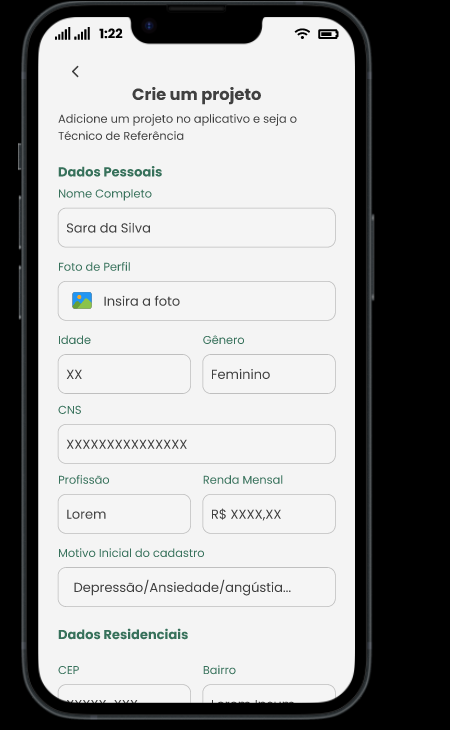

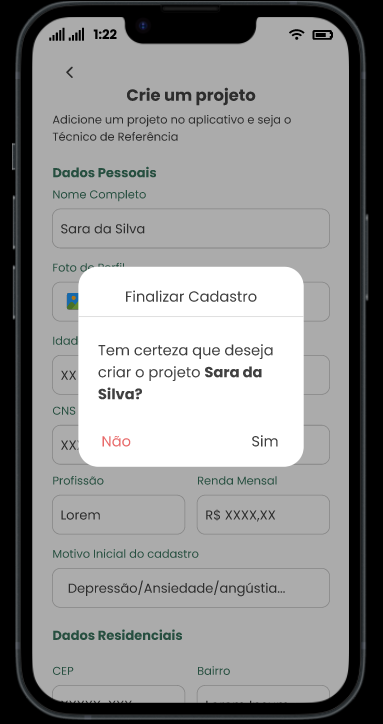


**Fonte**: Software Figma (2024)**.**
