# Supplementary material for: Development of the Psychosocial Rehabilitation Web Application (Psychosocial Rehab App)
Source: Nurs Rep. 2025 Jun 25;15(7):228. doi: 10.3390/nursrep15070228 (PMC12300239; doi:10.3390/nursrep15070228)
Supplement: Supplementary file 1 [file nursrep-15-00228-s001.zip › Supplementary Data 4 - Field Diary.docx]

**Supplementary Data 4: FIELD DIARY: Monitoring the development of the "Psychosocial Rehabilitation Project App".**

| **TRANSCRIPTION OF THE SPEECHES OF THE PARTICIPANTS OF THE MEETING 05/14/2024 (First Meeting).** |
| --- |

Mobile, which is for mobile, and a larger version, which is for computer. The last applications that people have made of learning objects have been along this line of webapp, which as they are "projects" involved without financial cumulation, the issue of maintenance is easier. That's what TIC 1 put it. For example, for you to take a product to an Apple Store, it costs 100 dollars a year. The way you register on the platform is quite complicated. Not to mention that you will need software, and also hardware resources, (....) to be able to make, compile and everything else... It's not that simple, as it is for Android. For Android it's easier and the cost is also lower. With Android you buy an account, through that account you can publish multiple apps, but on the other hand, if you only have Android, you restrict your audience. And for you, if you keep thinking, about iOS, you have an approximate annual cost of about 700 reais. If you don't make a profit from it, you have to see how much it's worth. And in the webapp, you can make this available in one place and the person accessed through a link, but through their cell phone it seems... that she is accessing an application (TIC 3).

I think that the first question, if it is okay for the researcher, I do believe, I believe that at first we follow this line (developing the WebApp), it is more, more practical (ICT 1).

So, I agree, the researcher, he understands mental health... (collective laughter). So, I think that's it..., I just have one question, because the app will generate data... And then through WebApp, what would this storage look like? It would be on people's cell phones, on the spot or we would be in a database... (Researcher).

The database is restricted to the database. And then, of course, the concern with security, we are going to do treatments for this, for example, there are some data that we can also do this, including passwords are like this, when there is a user's password that it goes to the database, even the people who created the database, We can't view this password, because it's encrypted data. So we can also follow this line, if we have other data, in relator to security, we can guarantee that everything will come out 100%. I'm going to present my screen here. OK? For us to talk about the requirements. You're getting it, right? So, this is the home page of the application about prototyping (screen 3 second version of the prototype of the "Psychosocial Rehabilitation Project App"). And here, my first doubt arose. This home, is it for a presentation of what the app is? (ICT 1). Yes, it goes to the screens as I idealized the application will be, right?! Now, I don't know if I answered your question (researcher).

Yes, yes. So, this is another question as well. For example, this question about the design of the application. If we change the design, but we keep all the features, is it okay? (ICT 1).

It's great, no problem (Researcher).

Perfect, then. So, we can leave Home as a presentation of the app. Of what it is, of what the user... Than you can add the user and everything else. On another health issue. It's this first page here. That's what I need to understand, you know? I need you to help me understand the idea behind the app, an idea that every screen does (ICT 1).

Understand... (Researcher).

So, I think that's the first page, right?! We can keep a presentation of the project with the videos (ICT 1).

Certo (Researcher).

Perfect. Here the first doubt arose. This little button here for Start, would it be kind of a Start for the videos?! (ICT 1).

This part, it was just to show that this part of starting Start... Now I realized that it doesn't make sense (Researcher).

No, no, don't worry. That's why we analyzed the prototype and that's why we're talking about it, you know?! Because we really go, we know that it is not your responsibility, but the technology part and that you stay in this part more of your area. We will take your idea and we will bring it to technology. That's why we were dealing with these requirements. Here, in this second part, I think it goes into the app project, right?" (screen 5). And is it the patient's records? (screen 8) (ICT 1).

That would be, the idea would be here how to insert a new project and there it is inside it, click here for you to see in the insert. (screen 5). Here's an example of how the projects would be inserted on this screen. Then go back there a little bit... Here, click here on the insert (screen 5), insert "new psychosocial rehabilitation project". So, here is the patient data (screen 8). So, we thought here... which is the normal registry that would be (Researcher).

We can register projects and each project will have several patients (ICT 1). Yes, each patient will have a project that is this structure that is here, patient data, situational diagnosis, goals, interventions, agreements, case study agenda and evaluation (screens 8 to 16). So, here we have the part of projects and patients, and as each project is going to be done, it will appear there (Researcher). Are these issues here defined in the project? (screen 5) (ICT 1).

From the project individually of TIC 1 (Researcher).

Oh yes, I get it (ICT 1).

Did you understand? For example, if I want to continue writing on it or opening it, it was to be able to go specific to TIC 2. There the other command is to insert a "new project". Then you, this icon was nothing, it was just to say that it's done, and this... No, pardon me, it's evolution, which was evolution apart from the patient, and X was to exclude (Researcher).

Right, perfect. So, this question is as if it were a mural. You have a project, you have the patients within the project, and then you create details for the project, to monitor the patient's evolution (ICT 1).

No, it's similar. So, it would be like, for example, there you insert a new project, this is a mural, here it will attract all the patients who have the projects open. If I click, go inside to see the patient's data or to enter, the diagnosis, the goal, the agreement... And within this project there is evolution, but then I want what?! So... It's a quick link to this evolution (screen 16). I don't know if what I just said made sense... (Researcher).

Okay, there will be patient data, but is evolution the most important category? (ICT 1).

It is an important category because I will be able, for example, to imagine that this patient, I have already filled in everything about him and there is something, it is how this evolution is a general record of the follow-up of this patient. Did you understand?! ... (ICT 1).

So he falls into these categories... (ICT 1).

It will stay, this evolution, it will stay at the end of this category there, in inserting a project. Let's go, for example, understand all these here, it will be at the end here in this one below the project evaluation or it can also be within the project evaluation (screens 15 and 16) (Researcher).

This I am asking for those who understand how the flow will be and what you need from the application (ICT 1).

But the application, like its skeleton, is this insert with these topics (screen 5) (Researcher).

Understand, it's like the most important part. Perfect! Let's go to another part, which is this sharing (screen 21). Here we share the patient's information?! (ICT 1).

From the patient to the professionals, PRP operators. Remember that the idea is to manage the psychosocial rehabilitation process and improve interprofessional communication?! Equally, for example, imagine that I need, there is a bakery course there at SEBRAE, then I call this person for the case study, who will teach the course, the institution of SEBRAE, and then I want to forward this agreement to this person and, for example, some question from the patient, But the one who will forward this is the professional, then he will score, you know?! Or, for example, I made my interventions with the patient, and I want to forward them to him, so he can remember. I want to send the agenda of the people, of the meetings I have, which is the case study. So, this is the most... it is a meaning that has been thought of (Researcher). Understand. And that's right! So we can share this patient's data (screen 21). This format, do you think it would be in PDF?! How do you imagine? (ICT 1). I had imagined it by a link opening the internet or it could be a PDF, no problem (Researcher).

Okay, I get it. So here we share patient information, as if we were going to generate reports (ICT 1).

Right!. (Researcher).

Right, perfect. And there's another tab here, which is this support tab (screen 3 and 17). Is this an aid to the user of the application?! (ICT 1).

This, to the professional. That there will be these references (screens 17, 18, 19 and 20) ... (Researcher).

There's a search too, right? (screen 20) (ICT 1).

This is the search to find that what I told you, remember? For me to see, when entering the patient's address, which support network devices for psychiatric patients are close to him, such as health centers, community center, school... Everything according to his PRP is programmed and managed by the mental health professional (Researcher).

Understand. Understand. And here, in the last one, there is this printing part (screen 22). Would this printing part be kind of the report, do you agree with me, that we generate in this part? (ICT 1).

The impression would be the same logic as sending the link (screen 21), which was a mental health professional (nurse 3), who suggested that many times the patient may not have a phone number and wanted to send the agreements with him, the agreements that were made, his intervention to know and then he could print it. It would be the same logic as sending the link (screen 21), but it is the possibility of being printed, if the patient does not have a personal smartphone (Researcher).

I understand, I understood correctly. So, I think that's the main idea of the app, right? We will have a presentation of what it is, we will have what we can say is the main part of it, which we call CRUD, which is the registration, reading, editing, deletion of information in the application, kind of total control of it. We will also have the evolution of the patient, and we can take this patient data, generate reports to send these reports, or we can take this data, generate the reports and print them. That's it?! (ICT 1).

That's it! (Researcher).

Closed! I think that here my doubts have been resolved. If anyone has any notes, something else... (ICT 1).

I think the doubts arose more when we developed the webapp... Now seeing it like this, in general, you explained it well... (ICT 2).

And it also seems relatively simple. It seems like something that won't take so much work in this way (ICT 1).

That's good! then if you want to get my phone, you can call me, have any doubt (Researcher).

| **TRANSCRIPTION OF THE SPEECHES OF THE PARTICIPANTS OF THE MEETING 05/22/2024 (Unofficial Meeting).** |
| --- |

For example in the presentation of the videos (Home screen 3), I will even share it here on Google Meet, and you can help me with that. I said that in this part you are much better than us... (ICT 1).

Here is the home and the presentation of the videos ok... Apparently there is not much secret in this part. No start (screen menu 3) here is something we can change this (ICT 1).

Then you can stay with you, it's an illustration that I wanted to present as the initial presentation of the application (Researcher) would be.

Perfect! That's what we can change a little. This is my question. In the "insert the new psychosocial rehabilitation project" (screen 5), are the psychosocial rehabilitation projects patient? (ICT 1).

Yes, it is the patients. But in the sense, ICT 1, that, for example, when a psychosocial rehabilitation project is carried out, when "the new psychosocial rehabilitation project is inserted" (screen 5), the mental health professional inserts this project that is about the patient, and the idea is that the patient, his name and the project that are under construction or completed (Researcher) appear.

Endorsement! Let's see if I understand! So does the patient represent a psychosocial rehabilitation project? (ICT 1).

This is a psychosocial rehabilitation project for each patient, the example of TIC 2 (screen 6), because the skeleton of the "Psychosocial Rehabilitation Project App" is between page 8 to 14, so each patient will have a psychosocial rehabilitation project, I don't know if it is clear now?! (Researcher).

Because that's what I wasn't understanding... Because there were patients, here, screen 5, so I thought that the professional would create a project, and he would insert, for example, 2 or 3 patients in this project (ICT 1).

| **Profile of the user/mental health professional/Reference Technician** |
| --- |
| Name: |
| Gender: ( ) Female ( ) Male |
| Race: ( ) White ( ) Black ( ) Brown ( ) Yellow ( ) Indigenous |
| CPF: |
| Profession: ( )Nurse ( )Physiotherapist ( )Psychologist ( ) Physical Education Professional ( )Nutritionist ( )Dentist ( )Pharmacist ( )Biomedical ( )Speech Therapist |
| ( ) Pedagogue ( ) Music Therapist ( ) Physician ( ) Psychiatrist ( ) Clinical Psychologist |
| ( ) Mental Health Psychologist ( ) Psychiatric Nurse/Mental Health ( ) Social Worker |
| Contacts: |
| Phone: WhatsApp: Email: |
| Personal Address: |
| Street: Nº Neighborhood: State Country |
| Professional Address: |
| Street: Nº Neighborhood: State Country |
| Workplace: |
| ( ) CAPS I |
| ( ) CAPS II |
| ( ) CAPS III |
| ( ) CAPS Ad I |
| ( ) CAPS Ad II |
| ( ) CAPS AD III |
| ( ) Mental Health Beds in General Hospitals |
| ( ) Hospital – Day |
| ( ) Street Office |
| ( ) Specialized Mental Health Team |

| **TRANSCRIPTION OF THE SPEECHES OF THE PARTICIPANTS OF THE MEETING 05/26/2024 (Unofficial Meeting).** |
| --- |

Good evening, Researcher, I have a question about the system. These project topics (screen 7) are fixed (they will always be this user/project independent) or they can be changed. Can the professional create or remove any? (ICT 1).

Ein!, ICT 1, how are you? Look, ICT 1, these topics will always be fixed and cyclical. The professional can't remove it, but he can always come back to it in a circle shape, I don't know if you understand? (Researcher).

Let me see if I understand. So, it's like a timeline. You will, for example, add things as if it were the patient's evolution. And then you can go back to a topic to see kind of what you put in the past. That was it? (ICT 1).

Not only go back what I put in the past, that's right, like timeline strategies, but I can also edit, put new information (Researcher).

I understand, I understand, right. And I was thinking too, Researcher. I don't know if it's about your need, but each topic is given, from the patient, goals, intervention, it's kind of a fully editable field, as if it were a Word. You can put texts, you can put images. If you need to put a print, if you need to take a photo of a document, put it. In this idea, more or less, the topics. Or does each topic have a specific little thing? (ICT 1).

That's right, ICT 1, okay?! But try to make the Word document, but inside the window, don't you? It won't be Word like what we see on the PC, right? That's right, in this way (Researcher).

Okay, Researcher, thank you (ICT 1).

| **TRANSCRIPTION OF THE SPEECHES OF THE PARTICIPANTS OF THE MEETING 06/05/2024 (Unofficial Meeting).** |
| --- |

**Figure 1:** Illustrative graphic design that presents the graphic design of the "Psychosocial Rehabilitation Project App"


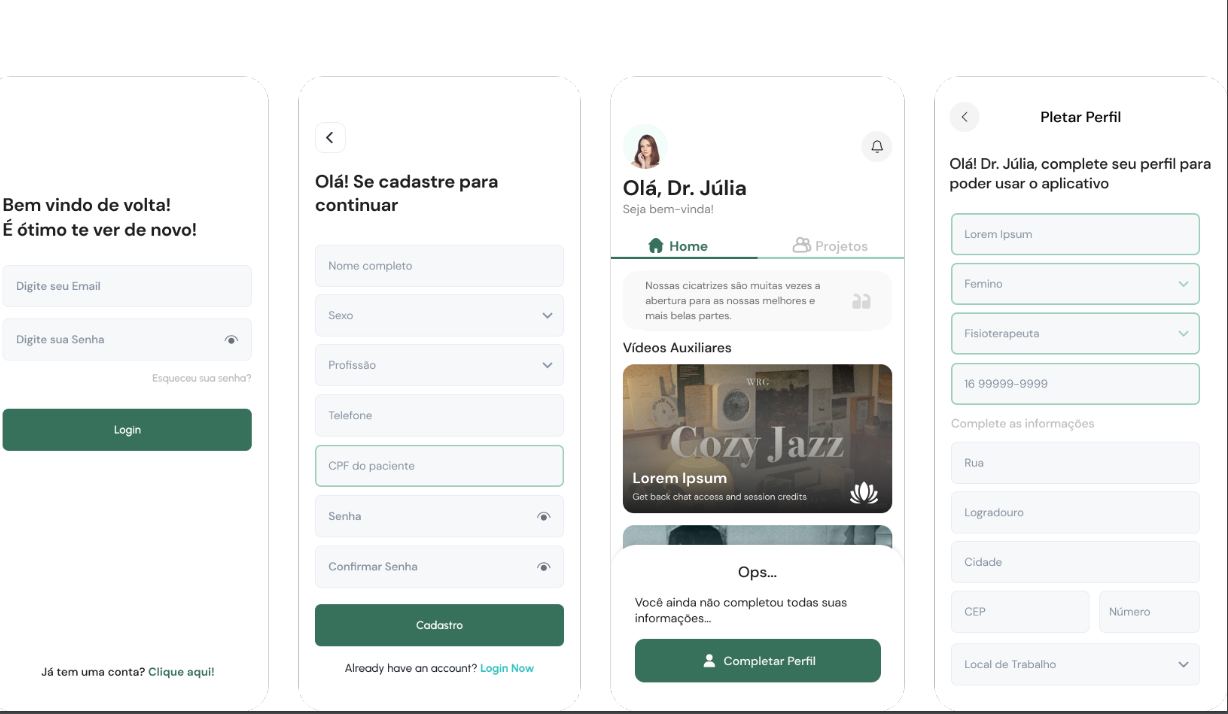


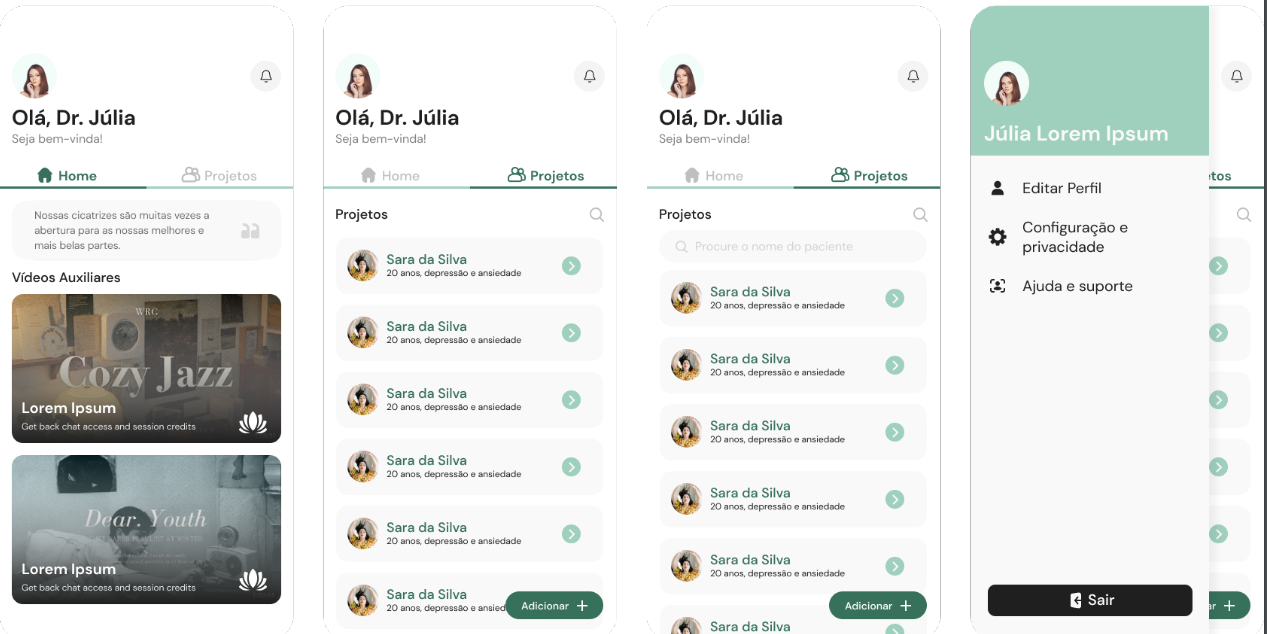


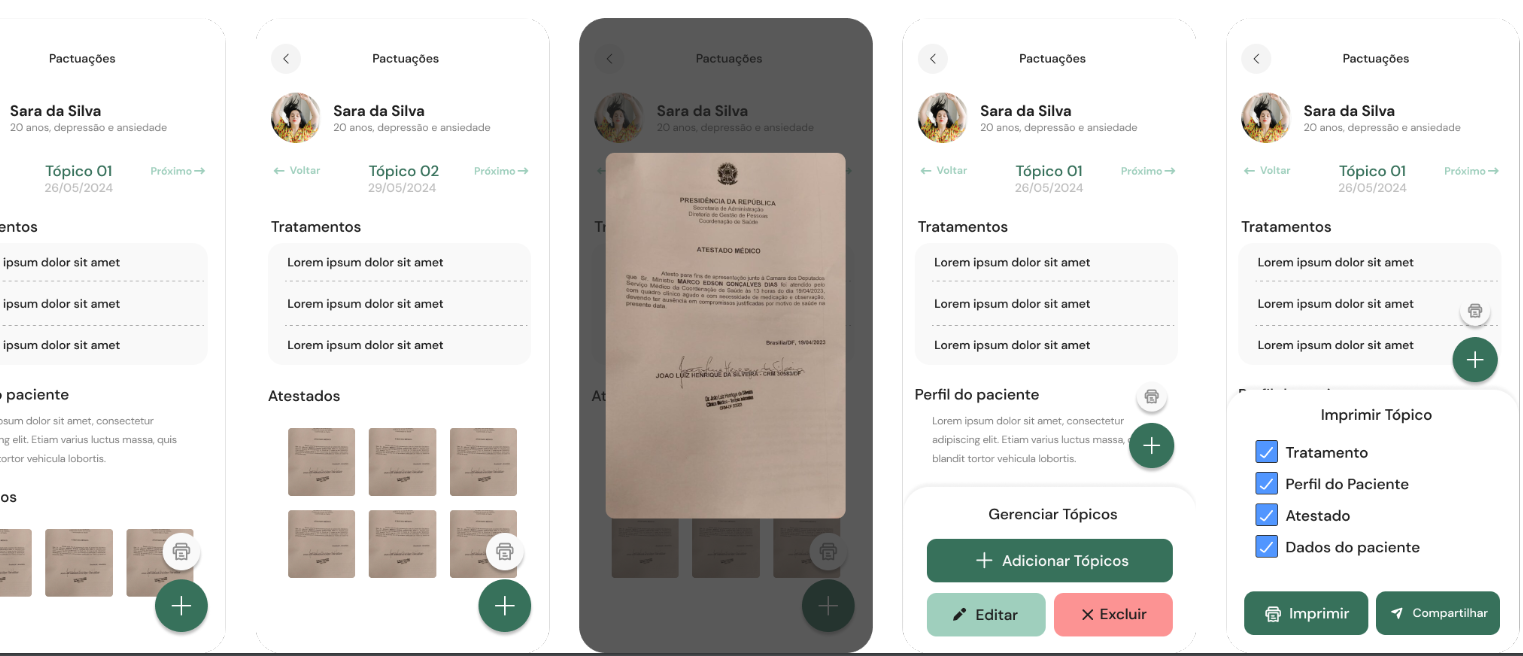


**Source:** Figma (2024).

The objective of the meeting is to present the graphic design of the "App psychosocial rehabilitation project" that will subsidize the developers in its construction. For this and for actual development, we need your feedback, if it meets your needs, if you think it's good, if you want us to change something. This one, I'm going to start introducing, this one is the registration login screen. Very normal, simple login screen, the registration screen continues in what the health professional will only be able to register from the moment he puts ONE PATIENT'S CPF (Emphasis added), and then he already links it to the patient. I thought about this issue because maybe we limit health professionals, you can only register if you already have a professional, or a patient, by the way. Here is the continuation, the health professional who registers will have this data, but he will also have an address, he will have other questions. And then it's a very large form. Instead of putting this in the register, we can register him missing some information, after he enters the application, we can bar his use while he does not complete the profile yet. Because we can limit this. Instead of putting a very large form in this part, we throw this information for when he enters the application (ICT 1).

A running text, or you can insert photos. Did you understand?! (ICT 1). I understand! (Researcher).

And then, when he completes the profile, he removes that name to this page to put this other information. As a workplace, street (...) city. This one would be the registration part (TIC 1)

It's calm, let me just understand. I wish there was a link here before in the registration so we can put it so he already knows the data protection and privacy policy. That's it, for him it's already... And here saying that he is aware, right?! of this policy... And one thing I'm thinking about here, ICT 1, is that at this moment it wasn't very good, this registration of yours, but we put the patient's CPF, I think that, I don't know if it's going to be, a good one, if it wouldn't be bad here on this app user screen, here, we demand the patient's CPF? (Researcher).

Exactly, he will not be able to use the application without determining his registration, because the other parts of the application need the data that he has not yet entered (TIC 1).

But ok, I think it is still possible, but how, for example, did he put this CPF and go straight there to his first project? (Researcher).

I might not link the project, because maybe there is no one registered yet, but I would already link the patient. Then we got into it. As I am the reference technician of this patient, you know?! Then I control if I want to show his visibility, what I want to do with him. Then it would link the patient together with the health professional (ICT 1).

Okay, but here, for example, if no one has the patient's first contact yet, who will be the first to enter this CPF? Like, for example, if no one has yet entered it because if each person is going to register, it is conditioned to a CPF if no one has yet entered the CPF of the first patient, right!! And then we may have a problem of not being able to register... (Researcher).

You can leave it there. For him to do later, right, maybe (ICT 2). According to the screen, perhaps? (ICT 1).

But, like, do you want to register the patient's CPF, yes or no?! Otherwise, he can do it later, right, too. Because then... Nothing would appear, right?!!!? (ICT 1).

Also this possibility. Because if it's going to be for later, then it's better to leave... for later in the project, isn't it? (Researcher).

Time to create the project?! (ICT 1).

Here I also don't know how to answer, at this moment how would it be... I only brought up this question, because if you don't have the first CPF, no one has put it, but who will put it?! (Researcher).

Yes, for example, we can put this manually, right? Via, for example, a database. Only... What I think, like, is the issue of security, you know?! When it comes to creating a project. He will need to type something. Like for example, I'm a psychologist, I can't visualize all the patients and choose which one I want. Because then it opens the door to malicious intent, right?! It opens a gap for me to have patient data, to be able to change and view patients who are not really mine. The system cannot allow this. I think we can keep this issue of the patient's CPF because it is a unique key, but maybe not here. But I think it's interesting that we keep this issue (ICT 1).

I understood, I think it's cool, yes, the CPF. That was a very good idea, okay?! But on the other hand, there is a problem that is being given by choosing that he stays at the beginning, because he will not be able to access it, because how will the index patients be?! (Researcher).

I understand. Now it's for this part, for us to discuss the ideas of how the system flows, which is this issue of the small screens. I'm now making the notes here too, and then we can correct the design. There is now the main menu part. This is the part of the main menu, which is the Home. These questions here, these little cards, they are videos. This Lorem Ipsum is something that we put because it's a random text, just to show that there will be a text there, but it hasn't been stipulated yet, so we don't know what to show, we put it just for the sake of visualization (Researcher).

It's perfect (Researcher).

(...) The videos, this phrase that I also put to have something, but everything can be changed, you can see it later in that report. This is the home part then (ICT 1).

I got it (Researcher).

When he changes the tab, he moves to the side, and then part of the projects remains, then he can see what projects he has, with age, what it is being treated with, all of this here can also be changed without any problem (ICT 1).

I thought this was really cool, this is everything here! At home, right.. he just changed... (ICT 1).

Exactly, just change it, it will only pass, it will pass to the side and it will have access to home along with the videos, it will pass to the side, it will have access to the projects. Then, it gives freedom to the user, what he wants to do... Here, the next part is the filter part. Imagine that he clicked on this button, he clicked on the filter button, we open a field for him to type the patient's name. And sometimes a, I'm a health professional who has several projects, this list will be extended, so it will go beyond the application screen, it will have to scroll down to view. Then here with a filter it's easier (ICT 1).

I understand. Leave it alone. Come back to the home. Then it was very cool. Let's just make it short. Let's leave just one video. Because then we don't leave much information. A video. And here I'm going to think, this sentence was very cool, I'm going to think of a phrase for us to put here, okay?! (Researcher).

Beauty (ICT 1).

It turned out great. So, this part like this, this idea... It was very cool. That's right. Okay, you can continue, it was very good. (Researcher).

Is there any highlight in this part of the project? (ICT 2).

No... Was... (Researcher).

Do you think it's okay for us to show here what is being discussed?! (ICT 1). Yes, the idea is great. And the place that shows the patient's name, age and diagnosis is very good (Researcher).

This little screen here, Researcher, it's called Drawer. What is the Drawer? The Drawer is when the person or health professional clicks on their photo. And then this screen appears, it scrolls vertically and the user's profile information appears. If he wants to edit profile, if he wants to see privacy settings, help and support, if he wants to log out of the app. So it's like a submenu. That submenu is hidden, it stays here. The person hit here, clicked, this submenu opens. She clicked off that part, it closes. It works in this little style. So, the main menu issue is this. It is the summary of the projects. And the home. (ICT 1).

Uhum... It was incredible, as I said so far. Very good (Researcher).

So, then we go here to the most complex and broad part of the app, which is the construction of the psychosocial rehabilitation project. I'm going to zoom in here. When a health professional clicks on the project, that's what it will open. These are all those topics of the psychosocial rehabilitation project, screen 7 of the prototype of the "Psychosocial rehabilitation project app" built by you (ICT 1).

And where will it click? To open the psychosocial rehabilitation project?... is... (Researcher).

To go to this part? (ICT 1).

This, to go to this part again, as if he were going to insert a new psychosocial rehabilitation project (Researcher).

Then he comes here, to add. There will be this little button here to add. Because think that here we are in the projects tab... (ICT 1).

Which are the ones he has already done, right?! Here, when he manages to click within a project like this, all the topics of that patient will appear that he will be able to edit, right?! (Researcher).

Exact. Exactly. And this is the initial part of the project (ICT 1).

But can I put it there on that add project button? (Researcher).

Perhaps for a more legitimate issue? (ICT 1) ... Right... (Researcher).

Clear. Perfect. So... This is that little part that we have in the topics of the project. Here I also thought of a new feature that he can already preview how many posts there are in that topic. So he doesn't have to click and see. Here we already achieve, for example, care and mental health goals. That is, quantify the number of topics that are... Then we can already have this information before. And here too, as the patient's evolution is as if it were a different topic, he stays in this scheme of the little button at the bottom, along with adding (ICT 1).

Legal! (Researcher).

Here we can differentiate that. Cool, cool. No, it's great. This one was cool (Researcher).

Here's an example of a detail in the topic, which he clicked and opened. This is an example of agreements. There will be information from the patient, we continue with it. And then we have topic 1, as if we had several topics registered. We have the option to go back and go to the next topic. And here we have the information on the topic. What did I think of information? We have three different types. This one that we can treat kind of by the balls, you know? How we make a document and we have those topics within each other. We have a running text and we have the option to register images. Then when registering, these three questions may appear to see what you want to register. Sometimes you want to register only a running text, sometimes you want to register only one image, sometimes you want only these separate parts. Maybe these separate parts are very interesting for patient data. You register name... which is to change something. Then in this matter of the detail of the topic, I thought of these three categories (ICT 1).

Repeat again for me, the detail of the three categories (Researcher).

Here again. He clicked on Pacts, he came to this screen. Here, this part is fixed, this part does not change. From here down, this part will change. Why? Here we are previewing the details of topic 1. So, if we are close, this will change because it will be the details of topic 2. It's like that issue of us registering the patient's evolution. Here he is visualizing the patient's evolution. Did you understand? So, what can you insert in the patient view? You can enter this type here of topic, which is separate information. You can either enter (TIC 1).

I understand. (Researcher).

This is the kind of thing you can insert into the patient topic (ICT 1).

I thought this logic was cool, but there's there, for example, remember that in the patient's data we have a text, some standard information that was validated and prototyped previously, remember?! (Researcher).

Yes, then the patient's data, for example, we can treat it differently because we will kind of... they are more fixed data. This here already has the data of the prank... Because of this, because it is something more changeable, you know?! (...) It could be just loose information and more photos. Here there was no need to write a running text, for example. These are kind of ideas for us to make these issues more customizable. Sometimes in the case study agenda, you don't want to put any loose information and no photos. In the study agenda, you want to put only one running text (ICT 1).

On the study agenda, here is just for... yes, I wouldn't need to, because it was just to remind the professional of the meeting's agenda and the day and time. But it is also this issue that you talked about, because it can be for several patients, it is not just an organization, but it is an agenda for him to organize meetings about patients with the team. I liked this idea of yours, yes, the way you put here to exemplify, I think it's valid. But we just need to pay attention to the skeleton of the psychosocial rehabilitation project. Let me, for example, I'm trying to get in there at... in that little business of ideas that I did, as it is because, for example, in the patient's data. I can't see how this will come in from here in all the steps. Like, for example, in the patient's data, he will have, like, that checklist, just like he made a professional record enter. Do you remember? I don't know if you are understanding my doubt.... (Researcher).

Do you want me to open here the link to the prototype of Marvel's "Psychosocial Rehabilitation Project App"? (ICT 1).

Oh, I wanted to (...), but so far it's amazing. That's right!!. It's just that from the evolution of these topics the icons... It was very usual for him to enter (Researcher).

It's just that we think about it when prototyping. And about these issues that the user doesn't have to learn anything about the system, it has to be intuitive. That he knows that when he presses to advance in the upper corner there is a little button to go back.... (ICT 1). Ein ICT 1. Come here on insert, insert. Go here in the patient's data (ICT 1 shows in Marvel screen 6 of the prototype of the "psychosocial rehabilitation project app"), that's it. Did you understand? Look, in the patient's data... we already have the structured information that you are going to put, such as name, age, then you put the CPF, you know, which I also put that you can leave, which is the CNS (National Health Card) that I use, the patient's address and telephone number, his profession, income, there is this information. This one about the diagnosis, so we already have it too. These are topics within it, subtopics of this diagnosis, which have, for example, the history of the patient's case and their diagnoses. And they are here. And here, look, and here it comes in, comes back there, the business here has the problems that it will identify because the project has an objective is for us to solve the problems of psychiatric patients, guided by the theory of psychosocial rehabilitation (Researcher).

Continuing here, this next step is about photos. I think we can also keep this issue, right? (ICT 1).

I think that to maintain this function of inserting the image, it is nice to keep it there in the evolution. Did you understand? Only there in evolution. If the user wants to enter... or rather... also there in the patient data. It's in the patient's data, he remembers, after we entered it. You can come back. So here, I get it... After we inserted all this from here, you can put an icon there in the patient data at the end for you to put images, right?! Attach image, files, I don't know if you understood, right? Because this will be important for the professional in the collection of patient data (Researcher).

That was my idea, Researcher. My idea was like that. We will support us to create a "new project", a new project topic. Sure? You want to add a pact. When you add a covenant, you'll have several different types of ways to enter data. So, for example, I'm adding a pact. I want to enter a data of the image type. So, I can do this in patient data, in goals, in agreements, in agendas. I think we don't need to limit ourselves, for example, photos only to patient data, you know? Whoever is creating, we give freedom to whoever is creating, enter the data he thinks is necessary for creation. I don't know if you agree with me (ICT 1).

I think this feature is cool, yes, I think it's good.... and it is personalized.... leave this freedom to the app user. But there is within this freedom, there is what I told you, that there is the project that is the structured method, which we cannot lose, which is this stage. And then, within this project, inserting these images does not make sense within, within, for example, the goal, the intervention. (Researcher).

It already exists as if it were a pre-structure... (ICT 1).

Right. What where there will be, the same for example here, now, in the agreements exist here as, for example, some images are the same for.... Here there in the patient data, at the end, about here, it can be in the patient data, or... So, like this, it would be there... more... Go to the diagnoses and... Diagnoses in mental health, that's it. So, here's where the patient... Where, in health, the patient will have images to insert. It will be more statements that he will bring, reports. So, the sense of this being valid will be more in this part of the situational diagnosis in mental health, in which the professional will consult so that he can know information about this patient. Did you understand? So, like, that's why I'm talking about the utility where it's going to be. Because here is information that is more intentional and oriented to help this patient in his psychosocial rehabilitation. So, what I want to say is that there won't be... It's not any image that will make sense... (Researcher).

For instance. One thing you said is that in this diagnosis there are some types of images, and I cannot decide to put an image in the diagnosis without being this type (ICT 1).

For example, the patient or family said that he is schizophrenic. Then he shows a report that has his diagnosis... And then it makes sense. The professional can put (Researcher).

(...) Can you send us this? For example, what photos make sense for the user to send in the diagnostic? What we can do, for example, at the time of registration... little button making insert report, insert, I don't know, attestation, something like that, you know?! (ICT 1).

That's it, that's what you're understanding. So here, but like this, in this Diagnosis registration, you can put it here, there is not much, it is a report, put it, insert an image of the report, a statement, let me see, a report (Researcher).

(...) We write it down. This (ICT 1).

Hey, Meet crashed. Let me write it down here that I send it to you. Calm down. So I'm going to put that in. Insert image of report, statement. Ein... there you are, dude... Here it starts to make sense... Look at what you brought... back there for the topic.... again... it's there in the sketches of the Marvel prototype. So look, here in the diagnosis I wrote, I see that for the image that will give, it will be to insert reports, statements and we can put other images. I'm going to send it to you. There we go to the goal.. it doesn't make sense, it's to put an image... (Researcher).

Got it (ICT 1).

Let's go to the intervention. Intervention (Researcher).

Tá (TIC 1).

An intervention... So... So also in the intervention I don't see the need to fix images... Now let's go to the agreement... You can go next time. That's it, now here in the agreement... I see... that may be necessary... insert the image, the minutes of the meeting with the agreements. Did you understand? Look, here it really is, they're going to need minutes of that meeting... (Researcher).

A question of mine. Can these mental health interventions occur in the case of a patient, for example, was he involuntarily hospitalized? (ICT 1).

It can happen from professionals... Look, go back to what I did with the intervention (screen 11 of the prototype). It may be that an intervention, it may be that the professional observes that the patient arrived and he referred him to the emergency room, referred him to be hospitalized. Did you understand? (Researcher).

I understand (ICT 1).

So, the disciplines, they go. depending on the patient's problems and situation. But then the Psychosocial Rehabilitation Project, it is not limited only to what is happening, but also to the macro, in the establishment of goals and interventions for psychosocial rehabilitation and the patient's quality of life. So, a long-term goal would be to socialize this patient in the community. So what was the problem? Patient with socialization deficit. An intervention would be to talk to the patient at the community center or at the health academy, then the person responsible will be the one who will help with this, or the reference technician himself, who is the one who is manipulating the application, the project, the user of this application who is with the patient's project, or it could be another person from the team, Or it could also be a person who was trained there in this health academy, okay?! And here is the deadline he would have to do this, you know? Like, for example... (Researcher).

I understand, I understood (ICT 1).

And the deadline... often it will not be a closed data... but so it could be "start in August 20224"... I don't know if you understood, right... (Researcher). I got it, okay... (ICT 1). And so, we organize this meeting, so we even build something round, because many things that are very different do that we do on paper. So I don't understand that even though I have this structure, in an idealizing way, here we are working in the world of ideas, you are helping to give reality and make it concrete and realistic. Because the goal is for people to be able to make the psychosocial rehabilitation project easier and less complicated than paper, because if it is this way, the application will memorize it, it will be a tool to help the mental health professional, because he will realize: Vein! How it helps me, how it improves communication, interaction, how with this application I can bring results to my patient, make my service more dynamic. What's up? (Researcher).

I understand, I understood (ICT 1 and ICT 2).

Do you think that in evaluation the need to insert images? (ICT 1).

Yes, there is and we can put it to insert minutes of the meeting (Researcher).

Minutes of the meeting? Every meeting, at the end, is it made a minute? (ICT 1).

Generally, yes. It's a way for us to co-take responsibility. I thought that's what you guys brought up with the idea of having an image. From me thinking, look, when will the image be generated? And then it's very cool, because then usually the meetings have to have minutes. And it's a way of co-responsible, because here it's giving parts that people are going to do, so having the document is easier, right?... I don't know if it made sense... (Researcher).

This also about the image is something like this, which at the beginning we don't think about, we think about it as it progresses. For example, you didn't see a need for this at first, I saw another need for this on my part, so now we join the two worlds into something that makes sense. This will also happen during the development of the "Psychosocial Rehabilitation Project App" (ICT 1).

And what will make it easier, right?! (Researcher).

Going back to the other canvases, so the image issue I think we're done, right?! ... (ICT 1).

Yes! (Researcher).

The question of what topics will be needed. This one, Researcher, what is this little menu here? This little menu is the menu at the bottom of the screen. When the user clicked more, what does it mean? It can add a new topic. So here he is in topic 1, but he wants to manage this topic because he typed something wrong. So he clicks on this + and it will open this little menu for him. If he wants to add a topic, he wants to edit that topic, or if he wants to delete that topic. This is kind of generic, because, for example, deleting and editing won't be all users who can do this. Here we are showing you as if you were the reference technician for this patient. Right. And then it's in this management part. The same thing in the printing part, because it is easier for the user to visualize. I think you're already selling the topic. Here, print out the topic, and you go to another page, and to another page show that same topic information. We can treat it the way it's happening here. If you click on print, which is a little button that is fixed at the bottom of the screen, it shows the information you want to print. So, what is this information here? These are the ones you typed, for example. I'm even going to open it up here to make it easier to view. You want to print the scheduled evaluation of this patient's project. That's what you want to print. But you want to go back to making invisible observations, because maybe it doesn't make sense to who you're showing this information to. And then when it's time to print, what will he do? It will show you if you want all this information, you know? This was my idea doing. You can say what you want to share or not share or print (ICT 1).

Perfect. And this sharing, then I will be able to share by email, WhatsApp?! (Researcher).

Yes (ICT 1).

This one, it stays in that same scheme, for example, when you download something that is in the browser, you download the PDF. You clicked on it, you clicked on share. And that's what the cell phone itself already does. It opens a tab below and then there is WhatsApp, there is Telegram, if you have it, email, Facebook... then he himself directed. In this share, I specifically think about generating a PDF of this. It's great! (Researcher).

And then in this print, too, both will generate PDF, but the share already goes to a specific tab on the cell phone. The printing is already going to another one, because perhaps this may be happening on a computer, or the cell phone itself may be connected to a printer (ICT 1).

That's right (Researcher).

It is a part of the patient's evolution, which I think is a more differentiated part. Here you will have as if it were a timeline showing the days of the publications. For example, on Monday, the 16th, there are these topics that Dr. Júlia (fictitious) made about the patient's evolution. And maybe there can also be more, because it started, but it went to someone else. So I think it's interesting to keep it like this, in the style of a social network, because then we can see who started the evolution of this patient. Because if it started and went to someone else, maybe it can also have the notes of the other. And she can visualize everything, as if she could visualize his entire timeline (ICT 1).

Cool, and that was the very meaning of evolution, but in this one, remember that here it cannot be a mixture of evolution of all patients, only of this specific patient, is that it?! Is it clear? (Researcher).

For example, we have the example of Sarah, who is a patient... (ICT 1).

Sara, that's it, then, for example, if everyone... Right... To see from all the world that had and if it has made evolution that has the access that can have Sara's access, isn't it.?! (Researcher).

Exactly! And here we clicked on add and, for example, what's marked in dark here, figure 1, you're seeing the evolution of that day. So, maybe in the second, when the project started, there was only a need for three evolution records. Therefore, we can manage this, we don't need to show everything. We can show it through sessions, through evaluations, I don't know how this issue of when evolution is recorded works. But we don't need to show everything, we can share it (ICT 1).

You can divide it or, as it is, you left it on your timeline and also keep the text running in chronological order, from the first information and the second, so that the person can visualize scrolling the bar I don't know if I think that's what you're proposing, too? (Researcher).

It's as if it were... Here it is as if it were really social network. You are selling the person's profile, for example, on Facebook. You are selling the person's profile, for example, on Facebook. Here it's not just the person who puts it, it's not the patient who puts it, it's the health professionals who put things on their profile. It is as if it were published. You are going to make a publication of evolution in the patient's profile (ICT 1).

And that's it. I just think it could, like, in a sub, equal, for example, it's here, what Dr. Júlia said, ah!, the time appears, it's perfect, that's right. It's calm, it's calm, it's calm. I just, I think it was cool, I managed to understand, yes, okay, okay, okay?! (Researcher).

The time appears and the day appears (TIC 1).

That's perfect, it was cool (Researcher).

On this issue of evolution then and of the topics we keep here (ICT 1).

Right! Very cool! We are trying to materialize the realistic form (Researcher).

And align these issues as well, but I think that there is more, for example, in this issue of an evaluation of the project, there is a meeting minutes. I think these are more technical issues, right?! (ICT 1).

That (Researcher).

That we need to align. These are more technical issues, what was that (ICT 1).

I'm going to write... and I will send it to you later. I did it here, then I send it to you in our group. I'll send it to you, so you can see where the image will be in the topics of the psychosocial rehabilitation project's screens. Then let me tell you... there's a little something... that was there that I put user support, that I had the references in psychosocial rehabilitation (Marvel, on screen 17). It's not showing up, not for me on Meet (Researcher).

yes, for me it's showing up (ICT 2).

What you did is showing up... of what the prototype is now that it's official for the app with the development interactions (Researcher).

So this part of user support will be here in Help and Support (Figure 1) (...) I can imagine it 100% round like this. I imagine professionals using it (ICT 1).

Oh, that's good. Do you have any more questions or do you think we've already done it?! (Researcher).

I think so. This stage here, these stages, in fact, we are at the beginning. We haven't started developing it yet. So, these steps are kind of the most important for people to ask a question about your prototype and align what the project actually is. So here, we're doing very well, we have to do that, we have to talk, we have to align things, add, remove, edit... because it is much easier for us to do this now than we see, this only happens when the app is "ready" (ICT 1).

I understood perfectly, I think like, for example, this alignment, which you're doing, is that when it, this design now, which is already from the app, because it's going to be very easy for you, because you're already there, and you just follow the assembly. Is that really what I'm thinking? (Researcher).

That's it, it's like the sketch here. We are seeing it as if it were the door of a house, and before we do it directly, we make its sketch beforehand. We sit down with the customer, we talk, we align, we see what he needs (ICT 1).

| **Message sent to TIC 1's WhatsApp, on the night of 06/05/2024, with the requested information.** |
| --- |

1. Proposed the phrase: "Permeating life projects with senses and meanings built in the habitat, social network and work" (Researcher).

2. Proposed resource to insert images/documents in the topics: Patient Data, Situational Diagnosis in Mental Health (reports, statements, opinions, other documents), Agreements (meeting minutes), Agenda (meeting minutes) and Evaluation (meeting minutes).

- Hi ICT 1, how are you? Look, I'm sending the message you asked me about inserting the image. I am placing the documents that are necessary according to the topics and it would be interesting to have another user of the option that as the professional inserts the documents is opened for him more options to insert. Regarding the patient's CPF at first I thought, do you know what I thought?! The important thing about the CPF, remember that it will be as you proposed, to be the control of who will have access to that patient. So what I think you can think of is a strategy of not putting it at the beginning to insert, but when in the patient's registration the professional inserts the patient's CPF is the condition. And anyone who has already used this patient should send a message to appear that this CPF needs the authorization of the other professional. And for those who sent this message of having the resource that this professional, he will accept or refuse access to this CPF and the project of this patient. Maybe with this data it will give you an idea of how to manage this, or maybe to have an administrative user resource. So I don't know, I just had this suggestion. Tell me if it makes sense and if you can perceive a technology resource that can solve this need" (Researcher).

- Hello, Researcher. No, right, I'm going to save, yes, those caveats and I'm going to take them to the next part of prototyping. And about CRUD, CRUD is Create, Read, Update and Delete. It's kind of a system through and through, you know? One type of system is that most, well, 99% of the system is this. What's what? Create is you entering data into the system, creating data. Create or Read is for you to read this data. Update is you edit this data and delete is you delete this data. It's a kind of system, you know? It's like an architecture, like that. It's a kind of way that a system is created. For example, this system that we are making is a CRUD, which is the information I told you about, because there are other types.

| **CONSTRUCTION OF THE ANIMATED VIDEO FOR THE HOME OF THE "Psychosocial Rehabilitation Project App"** |
| --- |

The content of the theoretical chapter of the thesis was synthesized, specifically in the topic "Psychosocial Rehabilitation Project", to be sent to the professional who will make the animated video.

Below is the summary material:

**PSYCHOSOCIAL REHABILITATION PROJECT**

PRP is a systematized method of care management and patient care in the mental health service, based on the theory of Psychosocial Rehabilitation (PR), and structured in the Singular Therapeutic Project (PTS) and Case Management (CM), which allows mental health professionals to diagnose the problems, psychosocial needs and demands of this patient, plan and manage their care, intervene, mobilize resources in the Psychosocial Care Network (RAPS) and/or community, make agreements and/or accountability in relation to the care to be provided to this patient, monitor, (re)evaluate and provide individualized, integral and humanistic care, aimed at the full exercise of citizenship.

Therefore, it is important to say that PRP articulates and operationalizes in mental health practice, PR, which is its foundational theory, and consists of a process that enables mental health service patients to achieve social functioning, self-determination and direction of their lives, independence, contractuality, social protagonism, citizenship, autonomy, occupation of social spaces and social insertion.

In turn, the PTS is an action plan that is shared and composed of a set of interventions that are guided by the intention of providing comprehensive and humanized care to the patient of the mental health service.

The CM, on the other hand, favors the management of the PRP, as it enables and operationalizes the management of the PRP, through its conduction, coordination, monitoring and (re)evaluation. Allowing the mental health professional to take over the coordination of the patient's PRP, with the function of managing it and ensuring that it is cared for with the support of the mental health team and any other social actor essential to the PRP.

The PRP has the following crucial objectives:

To ensure full citizenship through the interlocution and (re)construction of a global strategy of care for mental health patients in their scenarios of production of meanings of life: habitat, social networks and work.

To create conditions so that the relationships between the mental health service patient and the environment multiply and occur autonomously and opportunities are continuously available to them.

Identify which practices and conceptualizations are necessary conditions to be able to discuss PR, and elect priority demands and needs for the implementation of interventions.

Development of autonomy, independence, social functionality, social integration, citizenship and quality of life.

The structure of the PRP and are divided into four stages:

1) Assessment: it is a biopsycho-socio-spiritual assessment, which enables in-depth knowledge about the life history of the patient in the mental health service , medical and multiprofessional diagnoses, risks, vulnerabilities and difficulties, potentiality, desires/values, personal and professional interests and aspirations, significant social relationships in their territory.

2) Therapeutic Goals: they need to be contextualized and situated based on the singularities of the mental health service user, in accordance with the assumptions of PR and Psychiatric Reform.

3) Interventions and Division of Responsibilities: mental health actions are chosen to be instituted by the mental health team or any other relevant social actor to achieve the therapeutic goals in favor of the patient on care (and on its agreement). It is necessary to stipulate deadlines to be met (short, medium and long) and hold accountable those who will carry out the actions established in the goals or articulate their realization with the devices and/or professionals of the RAPS (social actors).

4) Reassessment: moment in which the evolution is discussed and the necessary adjustments are made, and other problems not contemplated in the initial assessment may be identified, difficulties, therapeutic failures or any other new need for care that arises, review/adjust interventions and agreements with the mental health service user and the other participating social actors.

Therefore, the "Psychosocial Rehabilitation Project App" will facilitate mental health professionals in conducting complex cases and their interactions with the social actors necessary for the development of PRP in mental health.

Briefly, we can summarize the assumptions of PRP that are subtended in the topics of the "Psychosocial Rehabilitation Project App": 1) PRP is a tool that enables PR for mental health service users. 2) PRP enables the construction of contractuality in patients of the mental health service through the development of care strategies that respond to their biological, psychosocial, affective and socioeconomic demands and needs. 3) The internal structure of a PRP is determined by the PTS, being broken down into the Evaluation, Therapeutic Goals, Interventions, and Division of Responsibilities and Reevaluation. 4) Case Management is a model that allows the management and operationality of the PRP through the interlocution between teamwork, case management, mobilization of resources available in the Psychosocial Care Network (RAPS), community and (re)evaluation of complex cases in mental health.

| **Official (07/12/2024) and unofficial (07/16/2024) meeting**: with the objective of presenting the graphic design of the "Psychosocial rehabilitation project App" finalized to start the development of the "Psychosocial rehabilitation project App". |
| --- |

Can you start? (ICT 1).

Pode (Researcher). This is the login screen. The login screen, the only change was the link to data protection and visibility. Then we can keep her here. And on the registration screen, what I had was the agreement with that privacy policy (ICT 1).

That (Researcher). Then we are still here with... Later I can send you this here, just this part of presentation... that you can comment there too. Then it gets easier (ICT 1). All right, no problem (Researcher).

The gender part. The registration is at that. And that's where the first question I have comes in (ICT 1).

You can talk (Researcher).

Will anyone be able to register? (ICT 1).

Anyone like that so you talk? (Researcher).

If we make this application available and we don't put anything to restrict anyone that, for example, if we play this application on the Play Store, can anyone who downloads it register? I had thought as long as it was those professions that I listed, remember there? (ICT 1).

It would be okay to do it, but I can't see a way to limit it at this moment. What did you have any idea thinking? (Researcher).

What I think we can do, for example, there were users of administrators and these administrators validated the user's registration. You download the application, you register, but you can't enter the application until another user, who has more permission than you, can validate it (TIC 1).

So cool, I think I could create this administrator of the application managers, which would be a place for us, I think it's cool, I think that's important. The administrator, yes. Necessary and valid, right? Only for the person (ICT 1).

As well as excluding, right? (ICT 1).

Yes, an account, right? (ICT 1).

Right. I think that for... That's what the user's registration is all about. There's not much of a secret. And here too don't put too much information because I think it's not convenient. I think it's more convenient for us to continue with what we were doing. To bar user input. If he has not yet completed his registration. Because from the moment he entered, there will already be screens that need data from him. So this will interrupt, it will disrupt the application (TIC 1).

Just repeat, I didn't understand what you said (Researcher).

Before the user will enter and he will see if it is his first login to the application, He will come across this small screen and he will not be able to navigate. He will need to click and complete the registration (TIC 1).

Ah, I get it (Researcher).

To continue his information, you know? (ICT 1).

That was perfect (Researcher).

Exact. And, for example, we can't let him enter the app because maybe on that screen, on those first screens, there is no data that he will use from it. As he advances, he does. So we can't let him move forward without him having entered his data in the application. He finishes his registration, continues information from the entire registration. There is race, CPF, profession. Profession we have several options. I don't know the place of work, but I put my profession in a different way. It clicks and kind of it gets redirected to a form within a form. And he can mark more than one as well (TIC 1).

Agreed, that's right (Researcher).

And the workplace, Researcher, can he also mark more than one? (ICT 1).

Pode (Researcher).

Can he work more than one place at the same time? (ICT 1).

Can. This, you can mark (Researcher).

Got it (ICT 1).

And then, this one doesn't have much of a secret, there's nothing special. And then we deal with it. Here is the home page of the application. Then it continues in the phase, this user support is a card to take him there. Here the videos. Let's just keep a video, okay? (ICT 1).

Certo (Researcher).

Then I put a bigger card here then, as if it were this one. The home itself is no big deal, but a real presentation. Then as we added more things, I changed the menu. It is no longer the menu up here, it is the menu as if it were from WhatsApp the user navigates underneath here we have the home tab which is more this presentation and here we have the projects part and we have this example here from Sarah and here there is the form of the button to add a project then if he clicks he goes to add, Here comes another question of mine, of course, you can say click on add project, I put it to that observation you pointed out last time, to make it more explicit. Here it is for the new, and if, for example, the trigger island will already appear here, if he clicked here on one of those that are listed, he would already enter the project (TIC 1).

Certo (Researcher). Here it adds, oh, let's add a project. A question of mine, will projects have photos? (ICT 1).

We hadn't thought about it... You say the patient's photo, right? (Researcher).

Exact (ICT 1).

But we can leave it, no problem. Get creative, if you think... Would this photo be than to identify or would be... In what sense did you think of this photo? Just identifying the face of this patient? (Researcher).

Yes, it's because, like, I think of it here as if it were an electronic medical record. We deal with the entry of a new patient, we deal with his treatment, and we just don't deal with the end of the process. But there is all this flow. I think that only the information, only data, only you read, is different from you visualizing. I think it would be more interesting for us to have this photo of the patient, but I also don't know if there is a case that will not be possible to take a photo (ICT 1).

As we are idealizing, there is nothing wrong with taking a picture. We will keep it. It was cool, I think it's creative. You can keep it, no problem (Researcher).

It's just that it's his form too. And all health professionals who create a project, they automatically become a reference technician. Right. And then they will have kind of authority over that project (ICT 1).

Very well (Researcher).

Exact. So this right here has the project form. We finish the registration and always when we change, delete or add, he always has to confirm to me that we treat this knows not to make wrong decisions within the application very good it was very cool then if it doesn't come back right and if yes he goes back to this screen and adds a new one then I'll enter for us to start talking about the project here it starts the part of sharing the project information. Can you get in just a little bit for me? If it doesn't get well, I can go to another method (ICT 1).

It's great. Now it's legible (Researcher).

Sure. Here we start with the information. Share his information, what you have a project for, but you want to share it with me, who am another health professional. But I don't have the need to include things. Maybe my need is just to read what is being inserted in that project. So when sharing, you can invite other technicians, but you have an option in which you will deal with the visibility of this project. Whether other technicians will be able to view or edit. Then editing implies adding, editing or deleting. Then there are kind of these two profiles, right? Has the viewer profile and editor profile (TIC 1).

And then he can go to send it to someone else, how will this send be? Because this feature was perfect. Ah, it could be for WhatsApp... (Researcher). This sending, it's kind of when you... For example, you're in an app, you click share, open this bottom edge and then there are kind of some apps there that you use. And then you can go up more and then you will have all the apps. Because I thought I'd do it through a link. Did you understand? You can send it by email, for example. You can send this link wherever you want (TIC 1).

It was perfect, ICT 1. That idea was exactly what it was (Researcher).

Or... that we can control what other health professionals do. This is the project part. This part of the project, honestly, she hasn't changed much. I added photos where I needed to add, for example, patient data. Here I think the photo begins, because here we already begin to visualize the patient. Then it gives more life, you know? (ICT 1).

Yes, this suggestion was very cool. Everything is ok so far. Give more life (Researcher).

And here we also start to deal with things in this little button. This button, it's the menu. Every time the user clicks on it, they will have several options. He can share that information, and then he can send it, for example, to WhatsApp, he can just generate the PDF, he can delete it too. If you click delete, a little option will appear for him to delete. And he can edit. Then I'm going to open here, close this little menu and I'm going to open the information. Here. This one comes in, I think the biggest doubt, like... (ICT 1).

Okay, you can talk (Researcher). For example, patient data is easy because it's that form of including a new patient, a new project. So, correct. Data, for example, personal data, other information, is not very secret. But, for example, institutional diagnosis in mental health. There are these various screens and here we have, for example, the history of the multiprofessional diagnosis case in mental health. We need to know what we need to have in this, you know? Because here is the example of a text. Here is an example of a text and a date. Know? What do you need to have in this? We needed examples of insertion, you know? What do you put into it? You enter a text with a date, a time. And here, for example, there is one that is medications in use. Ml, use of medication, stripe. Know? There are some things that are more specific (ICT 1).

I understood (Researcher). For example, clinical diseases. What do we have in diseases? Did you understand? (ICT 1).

I understand. Look, in sickness you can leave it in the text. Or else I can too... There you have it... When you send me the link will I get the little reminder to put? (Researcher).

Yes, yes (ICT 1).

I can list for you the main ones. Now, I understand the story. Now, the story of this case of multiprofessional diagnosis, here you have to leave the gap in the text for him to write. Here we won't have it ready, it will be at the discretion of the professional, he will do the running text. This too, for example (Researcher).

This is the example of a patient who is already all registered with her. We can't force the health professional to register everything when creating a new project, right? It is according to his treatment (TIC 1).

That (Researcher).

And also, for example, will every project necessarily have all these options or not? (ICT 1).

Well, everyone has to have these options (Researcher).

Do you necessarily have to have all the options? (ICT 1).

All options. But it will depend progressively. The professional builds, you know, necessary data. Then comes the diagnosis, then he goes on making the goals, the interventions. It may be, but this way, it leaves no prerequisite. It may be that he may already have to do the interventions first, it may be that the flow continues in reverse, but it will be up to the professional. Did you understand? Did it make sense? (Researcher).

Yes, yes, it did. Conforme, as if it were a timeline (ICT 1).

And this was a timeline, but it may be that, for example, he made the patient's data, the diagnosis, but it may be that this patient arrived in crisis, he succeeded, he will already have to intervene, so he will... The first thing he will do will be the intervention, and it may be that only the next time he sees the patient, that he will fill in the other questions, you know? (Researcher).

I understand. Then we can leave, for example, a... When he clicks, it's because he kind of already has an example of everything. For example, it is the agreement. There is an example of a pact. Yes. Then he clicks and opens... This is data that I put just to show, you know? (ICT 1).

But this data to show was what we had already talked about, isn't it?... The person in charge... Right... (Researcher).

It's just that there are some that really are, it's very specific, for example, it's... medication. Know? What do we have to put in a medication for... What is the field, do you know what I say? (ICT 1).

I understood. What do we have to... (Researcher).

Put, what is the field? (ICT 1). Okay, the medication, I think this is enough for us to solve. The medication is usually the name of the medication, we will have the name, it is the principle, let me just remember the name here. The amount, let me put here, dipyrone, for example, dipyrone is 500 mg 4 times a day. Generally, in this prescription of medicine comes the name, the milligram... the... the attendance on the day... the frequency and the route... if it will be oral... Endo... intramuscular... in the muscle... in the vein... So, generally, the standard of medication is this... is the name... the medication, which is the active ingredient, the milligram and the dosage, which we call it, which is the frequency of use. Sure. Or, look, in this very specific question, or you can let it be written that way, of the gaps, or leave the text too, right? The running text. Yes, the running text. It can be (ICT 1).

And then we kind of work with only two types of fields, right? Which are texts and images. For example, there were tables there. I was seeing about tables. But it was going to be very squeezed. I think it wouldn't be good for the user. And each field of a table becomes as if it were a topic (TIC 1).

And this creativity of the topic (Researcher) was so cool. I think that's the process. Instead of profession being a table and having the data underneath, profession it becomes as if it were a topic, you know? And below the field that is the result. But that's the part of the project. We have the projects, you can continue that. For example, inside is because there are several project categories, right? Categories here. Then, for example, within the diagnosis, there are already several categories, subcategories of diagnosis. Then he thinks that the user is in this place. He is in institutional diagnosis in mental health. Within diagnosis, he has several options. He can visualize what he wants, or he can move between them, as if it were the flow as well. Then it comes to the last one, it will always come back. Here, diagnosis. Then it continues in what it manages to advance. And here the little menu will always have it, right? He can always share or generate a PDF, he can delete, edit or add. I think that's what the project is all about, right? (ICT 1).

Right. Let me just see the intervention, how it turned out. The intervention, here (Researcher). The intervention was one of the cases that I didn't have much idea of what to put in. There are some things there, for example, goal, deadline (TIC 1).

That, but that's it, that's just the problem. But you have to remember that this... Also that I put a date, for me it's what to remember, you know? What needs a date, which is sometimes an entry that needs, right? Yes, that's important. The person in charge... Let me see the intervention. But like this, look, remember I told you that this problem is what he will identify there in that part at the beginning, remember? There in the evaluation. So, because there will be several problems and it can also be several interventions. So, you can't limit it to just one (Researcher).

No, yes. She can, for example, she can add more interventions, you know? (ICT 1).

Oh, I see. This here, we are showing the detail of an intervention. But she can add more interventions. And the interventions appear here (Researcher).

Exact. And this here too, this here are all examples of titles, you know? Sometimes there is, I don't know, intervention and the name of the place. I don't know. It's in these specific data that I think more of a part of you comes in than ours, you know? (ICT 1).

No, here look, here is it, it's mental health intervention and here it's intervention. He was putting it on, maybe... Click here on this first example of this intervention, when we get back there. Here the titles wouldn't change, right? (Researcher).

They would change their content (ICT 1).

I got it (Researcher).

He could advance too. To have an example of what it would be like (ICT 1).

Okay, let's ... let's go to the next one I think it's going well... That the project part is this, in several categories within each category, there will be more categories, right, and then you can add it here within that, I understand. There is also the filter here, right? (Researcher).

That sometimes registering a lot, it may be easier for the person to filter by the patient's name. And here too it was something that I don't know if it should be said. This is in the project, here. Initial reason for registration. Do you think it is appropriate for us to put what was the initial reason that the project was registered? (ICT 1).

This is not necessary. This is unnecessary, no (Researcher). Beauty. Now I can take that off. Now let's move on to the evolution part. That is a part... Before, evolution was in this little part here. But evolution, you agree with me, which is kind of as important as these categories, all of them. So, I believe it's better for us to treat this in just a small part of the menu. She can see the patient and his evolution. And then if you click, you have access, continuing that, you have access to the patient's evolution, Sarah's. You have the calendar of when the evolution was added. You can also navigate between them (ICT 1).

I got it (Researcher).

And another question here. Evolution, do we register only text? (ICT 1).

It can be, just text. I can't see another one the way you had thought? (Researcher).

No, it was a question I had, because I don't know if any photos are needed, Usually they are the types of field, you know? (ICT 1).

Usually it will be just the text. What would be good is that there was a text for him to insert, but he had suggestions of what was done, just like for example. Beyond the main problems he had... and the interventions and goals... the goals and interventions that have been worked on so far... to stay as a suggestion... then the professional, based on that, he... he was going to write his text (Researcher).

And in this, when creating? (ICT 1).

When creating (Researcher).

Right (ICT 1).

When it's time to make... insert this little square, as you have there from Dr. Júlia, right, that there were the suggestions of the... of the interventions, the goals they brought (Researcher).

Got it (ICT 1).

Then he would see that it was going to put, you know, his text (ICT 1).

Uhum, I got it (Researcher).

This is a remark he made. But it would really be just text. But we can put that when creating. Because I was also doubtful about the screen of... for us to register the evolution. I even forgot to put her here. So, suggestions for... (ICT 1).

Evolution... Suggestion of evolution in text and that visualization appears that is like when we do it, those word suggestions don't come to us, we just click and it appears, or gives enter, but what he has done so far with the patient would come, right? mainly the goals and problems, the goals and interventions. The problems he posed, the goals and the interventions (Researcher).

What we can do is a registration screen, within this registration screen you can finally have the data of this, of problems, goals and interventions. Because he thinks that every time we show something, this something that we are showing, it has to come from a place. So, it's okay for us to take it from another canvas and bring it to this one. We can show this data at the time he is going to do it (ICT 1).

Right! (Researcher). The evolution registration will be very similar to the project registration. Maybe here at the beginning of it we can put that, you know? Suggestion, something like that (ICT 1).

But that's it, but like this, but then you don't need to... Will this bunch of data appear again? Oh, it's going to show up, right? No, it is important. I think it's... (Researcher).

No, but evolution... It's because this right here is the project creation form. It is the form of creation of evolution. I haven't done it yet. I had these doubts (ICT 1).

Or, look, look at that cool thing. I think... I don't know if that's what you're trying to tell me, because when the text will be rushed for him to do, but that he can have the places where he can view the patient's record... the information he put there... in the diagnoses... that history... there's not that first one there... the situational diagnosis of the patient... that he would see the interventions... the goals and the interventions... but that he didn't have to come back... If it was... I don't know if it made sense to you... to see that we have all these topics, right? Sure. From the patient, but there's the macro topic, right? When you came back, Sarah (Researcher) entered.

Yes, there is the data, this here is categories, and then we have, we can visualize it, right? (ICT 1).

yes, how so? Then he goes back to the evolution, he will write the text, but you agree that for him to write this text, it would be nice if he could retrieve this information very quickly, but without losing it, having to go back there. Just like here, see that you have a timeline of interventions. If there was a way for him to be able to navigate this information, just visualize what he has for each of those tops that he has built so far, the patient, mainly, that about the data, those evaluations he has of him, the problems, the goals and interventions, because in evolution he will need to know that. I understand. Did you understand? If there was a resource that he could get (Researcher).

But do you think he would need to visualize everything or does he have some more important things? (ICT 1).

What would be most important for him to see at this point, look, all this main data here that you're showing on this screen, goes back to the... Here when you click on the diagnosis you go to that there, everything, right? Yes, so this one has to see and so it is indispensable, you know? Right. His data would no longer be so needed. I wouldn't need to see it. Understand? (Researcher).

Got it (ICT 1).

Now, the goals would be important. Very well. Then go back there. And the interventions. They would be very important (Researcher).

So these three? (ICT 1).

These three! (Researcher).

It can evolve (ICT 1).

There are agreements as well. I think it would be cool for him because he would see what was done, who is participating. These four topics would be nice to see there, but like, just visualize nothing that he could write at that moment there, you know? It would be like, look, do you understand when you click that it generates that timeline of interventions, of the evolutions that you made? If there was this, like this thing for him, you know? For each of those tops that I talked about (Researcher).

It's because what happens if we click here, we're going to redirect it to some other place, what I'm thinking about is how we bring this because it's a lot of information, do you agree with me? that's what I'm thinking because here we already have a lot of category and within category we have several (ICT 1).

So this would be ideal, because then one of the difficulties we have (Researcher) would not be lost.

I understand. Okay, this is a question. And the project information that we need to bring would be the diagnosis, goals, interventions and agreement (ICT 1).

And then, TIC 1, just go back to the main screen, to the project, to the one you brought me (Researcher).

This one? (ICT 1).

Right... But on the other hand, we also do not summarize this information. Wouldn't it exist here like today, when we have the resource of artificial intelligence, of it being able to give a summary? (Researcher).

Of this patient? For example, there is another project that we are doing, which is a Google project. And it uses artificial intelligence. But until that moment, it is free. In the future it may not be, you know? But I don't know how faithful it is for us to deliver a lot of data to him and we talk, summarize it. And what will he deliver to us, you know? I understood. Because you're dealing with... Do you agree with me? Which is something very serious, that he can't go wrong. AI has to take what the word represents, summarize and deliver what has been summarized perfectly (ICT 1).

I got it (Researcher).

Because we're dealing with a patient's story, you know? (ICT 1).

You're totally... correct... To try to minimize this pain, that we can't go to Evolution to see this data, what is it then... because I can't think of any other way (Researcher).

It's because here I realized that there are also some things, right? For example, there is the evaluation of the deadline and this person will evaluate whether or not the patient has met the deadline (...)If it increases a lot, you agree with me, it will be week (ICT 1).

You say like this, you're going to do an evaluation today, then in two months there will be a notification? (Researcher).

Yes (ICT 1).

Or having the notification, remember, of the goal, because here is the great feeling of this application, it is the evaluation where it will be interconnected with all those steps, especially the problem, the goal and intervention. So remember, in the goal, if he gave a deadline and made the intervention and also gave the deadline, it's to warn, look, the goal is coming up (Researcher).

Why did I ask that? We can treat this topic, evaluation of the psychosocial habilitation project, as if it were a mini evolution. Let's treat it not the mini, it's the evolution. Because it is a more systematized evolution and easy to rescue. My idea, for example, to force the user month by month, or two months to two months, to do the evaluation and here we can show statistics of this. Did you understand? (ICT 1).

Yes (Researcher).

This right here, this screen can be a generation of statistics taking the data from there. What is the percentage of goal that the patient meets in the short term, in the long term, you know? What were the goals that he managed to meet or that he managed to meet. For example, if the patient sets a goal that is recurrent, but in six months he has not been able to achieve it, we can have control of it and the health professor may be able to indicate to him to increase the deadline for this goal. I think that we can deal with this here more with statistics (ICT 1).

Great (Researcher).

So this screen would kind of be the follow-up. Evolution statistics. And indicators (ICT 1).

Because, think to me, we're going to have a feature and this feature will only happen in two months, correct? So we would have to, in theory, of course, wait two months just to test this, to make sure it's working. Of course, we can think that two months is a given and we can already change that. We can, for example, show this change every hour. So we know that it is working every hour. So we're going to increase this data to give two months, you know? (ICT 1).

I got it (Researcher).

(...) This is high-fidelity prototyping. So, you can use it as if it were really the application. What you can't get, for example, a field, you can't write in it, precisely because it's a prototyping, you know, it doesn't have this kind of function. For example, profile, exit, you can see the flow, what it looks like, (...), which is that you click and see the options, you know, open and close, log in, look there, login, then you go in, then you enter that little part, Then, for example, in this little part here you have the function of clicking on this part, because we need to move forward, Right? You can kind of circumvent this, but at the time you won't have it, right? Because we need to bar the user's entry. Then any questions just send it to us. And then, here you can move forward and use it as if it were really an application (ICT 1).

(...)

| **Transcription of the meetings and meetings held in Júlio, August, September, October, November and December 2024, January and February 2025** |
| --- |

In the meantime, let me talk about something. We're following that part of Figma. Which is that part of the design and prototyping. So we finished that prototyping part, it's those screens, but that part of the module that we talked about a long time ago, is about the administrative module, on the part of the administrator, that the user has to approve the login of the other user, not just anyone can register in the application. We are following this idea. This is the login screen. This is the app's main menu screen. The home part, the projects part. This is the patient's evolution part and the menu part. And when you enter the project, there are those options that we talked about. This here about color and everything, I'm leaving these details for the end and doing more of the main thing. Then here we have those options for patient data. They will have these options. We managed to do this here in a dynamic way. (...) But so far, that's what we have. The design of the prototyping, but about the actual development and creation of the application it is stopped here. It must be said that this is about 30% (ICT 1).

Hey ICT 1, let me tell you, so this part here is prototyping, isn't it? (Researcher).

No, prototyping nowadays is over. It's the menu the way it's going to look. Did you understand? (ICT 1).

I got it (Researcher).

The most refined design. Here we already have shadows on things, we have the cards. Then there is a little something, but that's how we work. We create the canvas, we create its function. And then, for example, this one that is a screen that is under construction, we now at the beginning, you take so many details of it, we will first work and then leave it in a more ... (ICT 1).

Cuter, more pleasant, just like this one (Researcher).

But for now that's it (ICT 1).

Ok (Researcher).

(...)

It's called Android Studio, let me show you, this is the build part. Buildar is like playing a game, you know? (ICT 1).

What is Buildar? (Researcher).

Build is when you co-build, it's build... It's when you set things up, for example here you write the code and then you want to see the result of it. Then you build, you know? (ICT 1).

I understand. That's how it works then. So, just so I understand, in this part is already the development, so... (Researcher).

Yes, we are in development, we are in the final stage (ICT 1).

Then, when it's over, that's when you'll have that idea of... of the... that TIC 3 talked about, to have the link to the application that you can see, use in any... on any device (Researcher).

Here, just like I showed you, here it's just the screen, imagine that it's just the device that we are accessing. This is an Android device, but this screen will be the same if you enter your cell phone, regardless of anyone, in the link on the website. Here it is as if it were in the application so that we can visualize what is happening (ICT 1).

I got it (Researcher).

But for now that's it (ICT 1).

(...)

The webapp is being developed in Visual Studio Code software with language and framework: Darte and Flutter, Database: Firestore, Authentication and Storage (TIC 1).

Market like this, it needs to be at least 6 days. Our CPF that comes from the profession to which she gave up (...). Here we also have the photo, so for now we took the photo, I'm getting it ready to choose (ICT 2).

How cool (Researcher).

This phone, registration, you're tidying up the bank (TIC 2). Are you going to walk to the place, are you going to register this here? Even this is the professional's registration, ok, that's right (Researcher).

Then I would register that. Saw? I'll see, the "guy" will be able to log in. So... would be the main page (...). I presented all the projects and in this case there is already a project (ICT 2).

I got it (ICT 2).

And also a research thing. I was doing this here, oh, registering patients. He had his name, date of birth, CNS, email, phone number, gender, profession, salary, residence, photo as well and what other information, would they come here too or would it be later? (ICT 1).

What do you mean let me see? Maybe supportive? (ICT 2).

It is in the registration as well. That leaves me just going up a little bit down a little bit just for me to see it right there. Another main piece of information is an address, a reference technician. That's right, that (Researcher).

Then I left... When he creates this account, his password will be the last 4 digits of the phone then it was the first login I talk about, you see here? First access, yes. Oh, from the illustrator too. That this would be the administrator's even for now. And the professionals who are already accepted and those who are pending (ICT 2).

So you can talk... (Researcher).

Here, in this case, oh, I would stay if I accepted. Then you would click on open a small screen with all his information, you could refuse or accept and that what would already be normal would already be there (ICT 2).

And then each one, for example, we will have, the administrator will have to access, this is to authorize each professional, right? That's okay, that's cool, right? And in the future, as, for example, you can divide this administrator, for example, the service is not yes either. But now this way is great. Let me just see the home page of the application, no (Researcher).

This comes in (ICT 1).

I'm very fast. Then I have the main presentation, right? Here will be the video that I will send you. It's here, right? (Researcher).

It's the part of projects, this evolution, I wasn't part of it (ICT 2).

That and let me, let me see. On the part of the projects, it was cool here you will put the additional structure, project. This, adds (Researcher).

Register. The patient. Is this CNS more effective? SUS Card (ICT 2).

Right, this is the patient's record, isn't it? (Researcher).

It is the patient's. Will it work? He was patient, but here it's not showing, but he's registered in the bank. Younger. He is already coming here, oh, it is registered that it was Paulo, I already register all the information (TIC 2).

What about the password, which is the last 2 phone? Then after he registers, he will send a table. What will it be? (ICT 2).

(...) (Researcher).

Normal patients, that's it, for now (TIC 2).

That's it, okay, okay, okay? That's what waiting is. Have a question? (Researcher).

Not so far, but in a little while. It is emerging, it is emerging (ICT 1).

Okay, do you think how much? (Researcher).

How many days can we see each other again? 15 days (TIC 1 and TIC 2).

So, okay, okay, let me, I'll share. It's just that I summarized our meetings that we had. To see if we can do it. I did one before, tell me a little bit like this, how is the development going? It's like that's like, what are you doing, in what application, what is it? As if you were able to describe you... What would it be like for a lay person, what are you doing, I think?... (Researcher).

That this is the part where we take the design that was created and we implement it via code, you know? We really build. Now we add the features, register a user. Before it was just an image. Now, in fact, we have a form, we put the information in this form and the data goes to a database. And then we get this data, you know? Now it is as if it were coming to life, the project is truly usable (ICT 1).

It would be the (...) the programming of the development of programming and where the software is called... resource (Researcher).

The framework language... That's the technology we're using to build this. You know, it's like a tool. There are several types of tools. And then we chose these to build (ICT 1).

This application is the framework for the ... (Researcher).

Technologies are also like it. As if we took Dart, which is a more brutal thing, and did more things to facilitate development. This is how they are tools (ICT 1).

Okay, very well. You have written wonderfully. I made a table and I'm not able to put it in our chat. Let me try again. I'll send it to WhatsApp, okay? See if you can do it. It's just that I synthesized those meetings we had, you can put them there for us to see. Look, I think so. I tried, that we had those meetings of discussing and I, I, I, I tried to structure. Because I think it was really cool here for us to understand the app, right? On the home, right? That comes the short video that we decided to be there, which was the one that excluded that start button, which was not cool. So look at the record, which was what you showed you have, the patient's, the professional's, isn't it? We remember that we are going to support the user. Where you will have it is mental health references. Legislation and the productivity record. Then comes the project, which is the topics. Isn't it that you had put the evaluation that remained? Diagnosis is not goals, interventions, agreement and evolution, okay? (Researcher).

And I think there's more... (ICT 1).

What is it? (Researcher).

Let me open the movie here quickly? Of the quantity, I think the quantity is a greater or we have a correct assessment, goals... Correct, correct agreements, case study agenda? (ICT 1).

This is the case study agenda (Researcher).

This. It's within the case. There are goals for mental health care, which is goals, right? And here too, we have patient data to put here. The way I share it is easier to visualize. I'm going to put one here, oh, this little part here is this part, correct? (ICT 1).

This, patient data, situational diagnosis, goals, interventions, agreement, case study agenda and rehabilitation. That's it? Is that right? (Researcher).

I think there was only a lack of diagnosis, you know, institutional diagnosis and mental health (ICT 1).

That's what it is in the place of evaluation. Then I'll replace it, so okay (ICT 1).

Ok (Researcher).

And there... of functions that we discussed was sharing, remember, which is sharing the topics report with what you did, a feature of WhatsApp, social networks and the search of the device by address (Researcher).

So it was just this one and the database that was the encryption, to connect too (ICT 1).

(...)

What is the name of the database? (Researcher). It calls firebase. That's what I typed here in the chat.

Now? (Researcher).

Is it if I can go deeper too? (Researcher).

Thus, firebase is the name of the that which is the name of the product. Within this product, we use 3 of their functions, you know? It has more functions, but we only use 3 (ICT 1).

And what are these functions that you use? (Researcher).

And there is always firebase before each. We can send it later so that each one is good (ICT 1).

Ok (...). So that's it, I'm going to send you the term of the conditions of use of the application, okay? I didn't send it. It's in PDF, OK! (Researcher).

We can sign... (ICT 1).

No, it's not the term for you to sign it's not from there isn't that one doesn't have a link. Before the person, this of the registration that there when you showed before that you accept to use the app (Researcher).

This one, oh (TIC 2).

That was when you entered?... There is no protection and viability there, there is no protection and viability somewhere at the time. I agree with that. This policy here I'm going to send the term to add, okay? Data protection, which would be the same thing, is the same (Researcher).

(The same) Thing (ICT 1).

It's the same thing, just to be aware of the person accepting it (Privacy and Data Security Policy Term). Okay, it's the same. It's the same thing, okay I'm going to read it just one more. Then I'll send it to you, can it be? (Researcher).

Maybe. (ICT1).

(...)

What can we do afterwards too?... when it has most of the features, we can do it, because how it works, it's as if we play this software that we use, then this screen appears that TIC 2 is moving, you know? As if it were the result of our code And it is done through a link, but this link is only on the TIC 2 machine... And then there is also a way for us to put this link in a way that you can access. So you can also see through your cell phone how it is looking, which then already has the most real use of it. (ICT 1).

That's cool (Researcher).

You can also see... (ICT 1).

To do. Do you think that by the end of 2024 you can finish it? Oh, so great, why what happens? Remember I said that we. I'm going to forward the link to technology professionals, so that they will apply a scale to see in a technical way how the application is doing, right? So, so that's it. I wish I was looking? Because then we will be very tight, because if it is done in the end, then I can solve it in January. Then you make the adjustments and in February it closes, okay? I am, I will, I am. Everything we're talking about, I'm helping the article, but when the article comes to an end, I'll send it to you, okay? So let's just schedule it for next time, in 15 days, I think... (Researcher).

Could (ICT 1).

(...)

Ball show, very cool, very beautiful. We see, don't we? Anything I'm available, you can call. Maximum, if I don't answer right away, it's because I'll be thinking, okay there, answer, I'll answer again, I'll send you, I'll send the term to TIC 1, I'll just read it and as soon as we finish the video, I'll send the video to put there in that link that is, But from then on I wait for you and what good is this project? And it's very cool. Congratulations, okay (Researcher).

Big hug, bye (ICT 1).

Bye (ICT 2).

(...)

Can you see? (ICT 1).

Yes, I am (Researcher).

(...) We'll show you standing there (ICT 2).

Beauty (Researcher).

Something came, now I understand what was there, I don't know. There is another screen there (ICT 2).

Already (Researcher).

Places small (TIC 1).

That's the simulation. It's the login screen that we changed too (ICT 2).

And it's the simulation of how it looks on the cell phone. That then it simulates the screen of a cell phone, you know? (ICT 1).

I logged in, here's the video, right? You click on this green whiting here and the video is (TIC 2). And the projects that are the people, right? (Researcher).

Here everything just teaching me to see, to test. That's where it works. And here everything is registering now. And I did a business like, to share a project. It was on a web like this, normal, oh. It copies the person's code, which in this case is... But when it's time to create it will be a bigger code and for cell phones it opens below, knowing the apps are patient data that I had to use, normal one, that I already had... here diagnosis, now it's registering register, here register (...). Test too I have already left it in the cloud. So you can go here and test it. It will be better for us (ICT 2).

OK. Just send me the login (Researcher).

It will be here. Evolution is also something else, which I have also done. Put one thing, he appears here... also the research... Here in this case, I did... Then he shows up too. Then this one, it would be at the time that we went to share the project. I shared, copied the link, which in this case has already opened, look, it's that way. (...) Then you would come here and research (ICT 2).

(...) This way of sharing is as if they had a code, then you share the other person's code, and then in that search, you link to them (ICT 1).

I think it's better to show the map as well. The map (RAPS service search resource) we had an idea like this. I think it has become easier. Because I don't think there's a way to mark it on the map. It's a more complicated business. What we have as a means is to score one by one. Like this, if we were... Then everything that has to do with mental health appears (ICT 2).

But that's it! (Researcher).

But on the map there is no way to show it (ICT 2).

You don't need to, you don't need to show it on the map. This way is what we really need. It shows the information, the information sought. That's right (Researcher).

(...)

Then we make your little screen cuter. What you can do too, which I think looks cool, is to also be able to put an arrow, if the person clicks on that arrow, then it opens that address on Google Maps (TIC 1).

Perfect, that's it. Very good! (Researcher).

There's also the guy's part, right? of ADM, right? (ICT 2).

He is (Researcher).

This is the module that is the account that creates the other accounts, both administrator and professional. We pass the link, we pass the accounts and the password email of each (ICT 1).

Agreed, then you send me the link and the account. But people, look, that's right, the proposal now is just finalize, that's coherent! I'll send... I'm finishing there from the references... In productivity like, if it's an option that doesn't have those balls, like, not that you can be free, what are you going to choose, right? That we already check the option. But then there has to be a feature that every time she enters the application, she can always be choosing. Does it make sense what I'm trying to say? Like, for example, she entered the app and went there and there will be, for example, individual care. Then she went there, scheduled, she left the app, entered again and she realized that, oh, she registered, she did a group service, then she scheduled, left and saved. Understand? (Researcher).

(...)

Where do you think you are? (Researcher).

At 90%? (ICT 1).

Oh yes, now you just have to see it (ICT 2).

We lack the mistakes... (Finish)... (ICT 2).

We tidied it up. But look, okay... The idea was cool, it was perfect. If there was a way... There is not that one... There in my prototype. That little brain you have? Like that little log? (Researcher).

Yes, yes (ICT 1).

Yes (ICT 2).

(...)

Cool, cool, then I'll go in but there's not much it's just, just, we line up what is is... (Researcher).

(...) You can tinker (TIC 2).

I'm going to snoop... just send me the link there on whatsapp and mine and the login for me..., but look... It's amazing, I don't even know what to say! This versatility was very cool! if it is in the application it is in the application, if it goes to the computer it is larger. That was top! Cool, cool, cool! (Researcher).

(...)

Plant some things, chat. So, we made the changes (TIC 1).

Is... (Researcher).

Now I think it's interesting that we already do a process, ... which is that we take what's on our server, which is this application, and we play it for that server that you rented. And then this process, it takes a long time. Before, it took about 12 hours. I believe that today is earlier. I think about 6 like that. If we do it now in the morning, in the afternoon it will be working. And then I need, I'm even going to share the screen here, which makes it easier for us. Can you see? (ICT 1).

(...)

And what does that mean? Which is hosted on this site? (Researcher).

That's how it is. I think if I explain it it's easier. We have here the place is our home, basically. The app is here now. What are we going to do? We will send this application to the public servant. Which is the www.com.br, for example. Or just... point... com, in our case. And then, this passage of the application from our home to the server calls the process that we are going to do now. Which is... hosting. That then we take him out of here and we put him in a place that everyone can visualize. Which is where you bought the domain. Then this process. On average, this here took a day. I think it's a deadline that they give. But it always tends to be faster. It's not usually all that, no. So today already... already falls (ICT 1).

Then when, for example, now, when you access the entire resource of the application, it will stay here, no longer where it was on this computer, is that it? (Researcher).

Instead of him staying here on the computer, for example, if something happens, I don't know, it burns, I don't know, he leaves here and goes to another place, you know? We take all the files, throw them to this public server here, which in this case is HostGator, right? And then they put... Then when someone types in Google, for example, any browser, in fact, Safari, for those who have an iPhone, reabilitasocial.com, then he will always enter. I also created, here in the app, a... Let me see if I'm sharing that screen, I think I am. (...) So, what do I recommend? I recommend me, you and TIC 2... We have our own administrator account because we can look at what's happening in the app. As if we were a step above permission, you know? We can do things, if we must do things that ordinary users don't do. So then I created an account for you too, then you even change the password, because the password is... It's your email, and then you can log in as an administrator (ICT 1).

Agreed, we woke up on that, everything is fine. Now I told you that there is this stage of my survey that they will evaluate, but they will not touch it, they will just enter the application and see if it is working or not and they will answer a questionnaire. That's what I want to know with you, what do you think is comfortable or important to create access for the evaluators or do they create it themselves when they enter? Will they be able to go with the email and password and log in? (Researcher).

Ô... Another question as well. Yes, for example, we thought of a code, right? This is the result of a code. That code, do you need it somewhere too? (ICT 1).

What is a code? (Researcher).

A code, oh, I'll even share it, because it's easier to show it. I'm going to take this screen out of here. I'll give you a big overview of what development is. Here is the real app, let's say. Everything we write, for example, this one is the login screen. Hello, welcome to Reabilita Social. If I put it here, test, the result of the code is the application. Then the test appeared here. This is like this, the functions, it's how she was set up, it's all under the hood. This here generates the application that we are using, that we are opening. Do you need that code somewhere? (ICT 1).

No, I won't need that in my thesis, no. I won't need it. of the code, but I think that, like, this code has to be saved, has to put it in some host, something? (Researcher).

So, there is a platform that we use, that we save there, it's a matter of security, you know? As if it were a drive. We always save codes in this place. To keep it safe, if we need it someday, we don't have it in our machine. We have it in this place, which is free, we leave it there and 100% safe. Because if we want to resume a day, regardless of the date, we can stop where we left off. What I ask is, because it has this validation, for people to use, technology professionals, it won't be that deep. So they're not going to look at code, that kind of thing. We need to show them (ICT 1).

So, from looking at the code, I don't think so. Then, when we finish this, before going to I'll schedule a meeting with you, so we can show you the instrument and we call, I'll send it to the technology professionals and we'll see what they request in this case, because it's based on the instruments... Now, the code is important for us to save, because what happens? Remember that I said that when it is ready we will register our names with the INPI, which is the registration of the code. I don't know, because they ask for a lot of things, I think they will ask for it. So, it's good for us to keep it, because when I finish my doctorate, and if the application goes through all the validations, we'll go in and have that conversation, so we can try to apply, and who knows that this application doesn't start to generate money. So, it is important for us to maintain all our partnership, and everything there is in the application, because it will enter the operation stage, and the improvement operation, and it can generate value, right? Perfect. Did you understand? So that's it now. Because I think it was like that a lot, okay? It seems that it's very cool that it's an app. Now we think, if it passed, validated, produced the product, okay? The first step now is when you close, it's... Then I'll sit down with you and we'll register the app. Then we'll see. We will register it in our name, as agreed. fly to this validation and there will be the last validation with more than 100 professionals that they will test, but this is until the end of the year... Then, for the post-doctorate, it's the implementation, we're at the end of the doctorate, in the last stages until May of next year (Researcher).

(...)

Ah, so that's pretty cool. Because it's an interest that we can get from wanting to play for the Play Store. You understand? (ICT 1).

Yes, absolutely. This is one of the advantages of the technology we used, which is called multiplatform, that we create a code and in this code we can use, just like we are using for a browser, for a Chrome, for a Safari, for any browser, the same code, we can use, play for the iPhone and play for Android, which is for the Play Store. So it's a more reusable thing. The resources, we are in the profile, help and support, I put the rest, the ordinances, patient evolution this one here I also changed the design that I think there were some mistakes here I put the patient's photo bigger and a little title... I thought it was really cool to participate, I think it's a very interesting project (ICT 1).

What did you find interesting? (Researcher).

I think one more theme, you know, because as in the area of technology development, normally what we do, we do website, we do inventory system, you know, there is a lot and concentrate on a different time, for example, this mental health is a different thing to work on. for a cooler theme, as if it were more useful, you know? It is more interesting to do this here for a user who is mental health and everything else than for a stock user, for example. It seems that it is a more (broad) thing... (ICT 1).

Oh, that's cool (Researcher).

That's it. Then I'll give you your access properly. Then you can test, create user. Here you can do whatever you want. You can create a user, you can create everything. Because then we can delete this data. So you can test it, you can create anything to see if it's working. You can be very free here. I am going... Then you can wait when you're already there on that platform for you to send me access, okay? (ICT 1).

Yes, yes. Tranquilo (Researcher).

Perfect (ICT 1).

Already, it's very beautiful, very pleasant. You were very... with an expertise... A very big learning experience. I hope that we close these stages and can build more partnerships for us to really make this work and maybe be a source of income for us (Researcher).

(...)

Presentation you know, there... And what then, agreed? (Researcher).

Agreed, agreed, bye (ICT 1).

Bye, bye, a hug (Researcher).

**Supplementary data 4: FIELD DIARY: Monitoring the development of the “Psychosocial Rehabilitation Project App” (portuguese).**

| **TRANSCRIÇÃO DAS FALAS DOS PARTICIPANTES DA REUNIÃO 14/05/2024 (Primeira Reunião).** |
| --- |

Mobile, que é para celular, e uma versão maior, que é para computador. Os últimos aplicativos que as pessoas têm feito de objetos de aprendizagem têm sido nessa linha de webapp, que como são "projetos" envolvidos sem cúmulo financeiro, ficam mais fáceis a questão da manutenção. Foi isso que o TIC 1 colocou. Por exemplo, para você levar um produto para uma loja da Apple Store, tem um custo de, se eu não me enganar, são 100 dólares anuais. A forma de você registrar na plataforma é bastante complicada. Sem falar que você vai precisar de softwares, e, também, de recursos de hardware, (....) para poder fazer, compilar e tudo mais... Não é tão simples assim, como para o Android. Para o Android é mais fácil e o custo também é menor. Com o Android você compra uma conta, por essa conta você pode publicar vários aplicativos, mas por outro lado, se você tem apenas o Android, você restringe seu público. E para você, se manter, pensando no iOS, você tem um custo aí anual aproximado de uns 700 reais. Se você não tem lucro com isso, tem que ver o quanto vale a pena. E no webapp, você pode disponibilizar isso num local e a pessoa acessada por um link, mas pelo celular dela parece... que ela está acessando um aplicativo (TIC 3).

Eu acho que a primeira questão, se tiver tudo bem para o pesquisador, eu acredito sim, eu que num primeiro momento a gente seguir essa linha (desenvolver o WebApp), ela é mais, mais prática (TIC 1).

Então, eu concordo, o pesquisador, ele entende de saúde mental... (risos coletivos). Então, eu acho que é isso..., só tenho uma dúvida, porque o aplicativo vai gerar dados... e aí pela webapp, como seria esse armazenamento? Ficaria no celular das pessoas, no local ou a gente ficaria num banco de dados... (Pesquisador).

O banco de dados fica restrito ao banco de dados. E aí, claro, a preocupação com a segurança, a gente vai fazer tratamentos para isso, por exemplo, existem alguns dados que a gente também pode fazer isso, inclusive as senhas são assim, quando tem uma senha de um usuário que ela vai para o banco de dados, mesmo a gente que criou o banco de dados, a gente não consegue visualizar essa senha, porque é um dado criptografado. Então a gente pode seguir também essa linha, se tiver outros dados, em relação à segurança, A gente consegue garantir que tudo vai sair 100%. Vou apresentar minha tela aqui. Tudo bem? Para a gente conversar sobre os requisitos. Você está conseguindo, certo? Então, esse aqui é a home do aplicativo sobre a prototipação (tela 3 segunda versão do protótipo do "App projeto de reabilitação psicossocial"). E aqui, surgiu a minha primeira dúvida. Essa home, ela serve para uma apresentação do que é o app? (TIC 1).
Isso, ela vai para as telas como eu idealizei que será o aplicativo, né?! Agora, não sei se eu respondi a sua pergunta (pesquisador).

Sim, sim. Então, essa é uma outra dúvida também. Por exemplo, essa questão sobre o design do aplicativo. Se a gente muda o design, mas a gente mantém todas as funcionalidades, tá tudo bem? (TIC 1).

Tá ótimo, sem problema (Pesquisador).

Perfeito, então. Então, a Home a gente pode deixar como uma apresentação do app. Do que é, do que o usuário... Do que você pode agregar o usuário e tudo mais. Sobre mais uma questão de saúde. É essa primeira página aqui. É isso que eu preciso entender, sabe? Eu preciso que você me ajude a entender a ideia por trás do aplicativo, uma ideia que cada tela faz (TIC 1).

Entendo... (Pesquisador).

Então, acho que a primeira página é isso, né?! A gente pode manter uma apresentação do projeto com os vídeos (TIC 1).

Certo (Pesquisador).

Perfeito. Aqui surgiu a primeira dúvida. Esse botãozinho aqui de Start, seria meio que um Start para os vídeos?! (TIC 1).

Essa parte, ela era só para mostrar que essa parte de iniciar o Start... Agora percebi que não faz sentido (Pesquisador).

Não, não, tranquilo. Por isso que a gente analisou o protótipo e por isso que a gente está conversando sobre, entendeu?! Porque a gente vai realmente, a gente sabe que não é da sua alçada, mas a parte da tecnologia e que você fica por essa parte mais da sua área. A gente vai pegar sua ideia e a gente vai trazer isso para a tecnologia. Por isso que a gente estava lidando com esses requisitos. Aqui, nessa segunda parte, eu acho que entra no projeto do aplicativo, né?" (tela 5). E são os cadastros do paciente? (tela 8) (TIC 1).

Isso, seria, a ideia seria aqui como inserir um novo projeto e ali está dentro dele, clique aqui para você ver no inserir. (tela 5). Aqui está um exemplo de como os projetos seriam inseridos nessa tela. Aí volta lá um pouquinho... Aqui, clique aqui no inserir (tela 5), inserir “novo projeto de reabilitação psicossocial”. Então, aqui estão os dados do paciente (tela 8). Então, a gente pensou aqui... que é o cadastro normal que seria (Pesquisador).

A gente pode cadastrar projetos e cada projeto terá vários pacientes (TIC 1).
Isso, cada paciente vai ter um projeto que é essa estrutura que está aqui, dados dos pacientes, diagnóstico situacional, metas, intervenções, pactuações, agenda de estudo de caso e avaliação (telas 8 a 16). Então, aqui a gente tem a parte de projetos e pacientes, e conforme vai ser fazendo cada projeto, vai aparecendo lá (Pesquisador).
Essas questões aqui estão definidas no projeto? (tela 5) (TIC 1).

Do projeto individualmente do TIC 1 (Pesquisador).

Ah tá, entendi (TIC 1).

Entendeu? Como por exemplo, se eu quiser continuar escrevendo nele ou abrindo, era para poder ir específico para o TIC 2. Lá o outro comando é para inserir um “novo projeto”. Aí você, esse ícone não era nada, era só para dizer que está feito, e esse... Não, perdão, é a evolução, que era a evolução à parte do paciente, e o X era para excluir (Pesquisador).

Certo, perfeito. Então, essa questão é como se fosse um mural. Você tem um projeto, você tem os pacientes dentro do projeto, e aí você cria detalhes para o projeto, para acompanhar a evolução do paciente (TIC 1).

Não, é parecido. Então, seria como, por exemplo, lá você inserir um novo projeto, isso é um mural mesmo, aqui atrairá todos os pacientes que estão com os projetos abertos. Se eu clicar, vá lá dentro para ver os dados do paciente ou para inserir, o diagnóstico, a meta, a pactuação... E dentro desse projeto tem a evolução, só que aí eu quero o quê?! Então... é um link rápido para essa evolução (tela 16). Não sei se fez sentido o que acabei de falar... (Pesquisador).

Certo, vão ter dados dos pacientes, mas a evolução é a categoria mais importante? (TIC 1).

É uma categoria importante porque eu vou poder, como por exemplo, imaginar que esse paciente, eu já preenchi tudo dele e tem alguma coisa, é como essa evolução é um registro geral do acompanhamento desse paciente. Entendeu?!... (TIC 1).

Então ele fica nessas categorias... (TIC 1).

Ele vai ficar, essa evolução, ela vai ficar no final dessa categoria lá, em se inserir projeto. Vamos por exemplo, entende todas essas daqui ela vai estar no final aqui nessa abaixo da avaliação do projeto ou pode estar dentro também da avaliação do projeto (telas 15 e 16) (Pesquisador).

Isso eu estou perguntando para quem entende como vai ser o fluxo e o que você precisa do aplicativo (TIC 1).

Só que o aplicativo assim o esqueleto dele é esse inserir com esses tópicos (tela 5) (Pesquisador).

Entenda, é como se fosse a parte mais importante. Perfeito! Vamos para uma outra parte, que é esse compartilhamento (tela 21). Aqui a gente compartilha as informações do paciente?! (TIC 1).

Do paciente com os profissionais, operadores do PRP. Lembra que a ideia é gerenciar o processo de reabilitação psicossocial e melhorar a comunicação interprofissional?! Igual, por exemplo, imagine que eu preciso, lá na minha intervenção, existe um curso de padaria lá no SEBRAE, aí eu chamo essa pessoa para o estudo de caso, quem vai dar o curso, a instituição do SEBRAE, e aí eu quero encaminhar essa pactuação para essa pessoa e, como por exemplo, alguma questão do paciente, mas quem vai encaminhar isso é o profissional, aí ele vai marcar, entende?! Ou então, como por exemplo, eu fiz as minhas intervenções com o paciente, e eu quero encaminhar pra ele, pra ele poder lembrar. Eu quero mandar a agenda das pessoas, das reuniões que eu tenho, que é o estudo de caso. Então, esse é o mais... é um sentido que foi pensado (Pesquisador).
Entendo. E está certo! Então a gente consegue compartilhar os dados desse paciente (tela 21). Esse formato, você acha que seria em PDF?! Como você imagina? (TIC 1).
Eu tinha imaginado por um link abrindo a internet ou pode ser um PDF, não tem problema (Pesquisador).

Certo, entendi. Então aqui a gente faz um compartilhamento das informações do paciente, como se a gente fosse gerar relatórios (TIC 1).

Isso!. (Pesquisador).

Certo, perfeito. E tem uma outra aba aqui, que é essa de suporte (tela 3 e 17). Isso é um auxílio ao usuário do aplicativo?! (TIC 1).

Isso, ao profissional. Que aí vão ter essas referências (telas 17, 18, 19 e 20) ... (Pesquisador).

Tem uma busca também, né? (tela 20) (TIC 1).

Essa é a busca é pra achar que aquilo que eu te falei, lembra? Pra eu ver, ao inserir o endereço do paciente, quais os dispositivos de rede de apoio ao paciente psiquiátrico, estão perto dele, como postos de saúde, centro comunitário, escola... Tudo de acordo com o PRP dele e programado e gerido pelo profissional de saúde mental (Pesquisador).

Entendo. Entendo. E aqui, na última, tem essa parte de impressão (tela 22). Essa parte de impressão seria meio que o relatório, você concorda comigo, que a gente gera nessa parte? (TIC 1).

A impressão seria a mesma lógica de enviar o link (tela 21), que foi um profissional de saúde mental (enfermeira 3), que sugeriu, que muitas vezes pode ser que o paciente não tenha um telefone e queria mandar as pactuações com ele, os acordos que foram feitos, a intervenção dele saber e aí ele poderia imprimir. Seria a mesma lógica de enviar o link (tela 21), porém é a possibilidade de ser impresso, se o paciente não tiver smartphone pessoal (Pesquisador).

Entendo, entendi certo. Então, acho que essa é a ideia principal do aplicativo, né? A gente vai ter uma apresentação do que é, a gente vai ter essa que a gente pode dizer que é a parte principal dele, que a gente chama de CRUD, que é o cadastro, leitura, edição, exclusão de informações no aplicativo, meio que o controle total dele. A gente vai ter também a evolução do paciente, e a gente pode pegar esses dados do paciente, gerar relatórios para enviar esses relatórios, ou a gente pode pegar esses dados, gerar os relatórios e imprimi-los. É isso?! (TIC 1).

É isso! (Pesquisador).

Fechou! eu acho que aqui as minhas dúvidas foram sanadas. Se alguém tiver algum apontamento, outra coisa... (TIC 1).

Acho que as dúvidas surgiram mais quando desenvolvemos o webapp... agora vendo assim, por geral, você explicou bem... (TIC 2).

E parece, também, algo relativamente simples. Parece algo que não vai dar tanto trabalho em assim (TIC 1).

Que bom! aí se você quiser pegar meu telefone, pode me ligar, tenha caso de dúvida (Pesquisador).

| **TRANSCRIÇÃO DAS FALAS DOS PARTICIPANTES DA REUNIÃO 22/05/2024 (Encontro Extraoficial).** |
| --- |

Por exemplo na apresentação dos vídeos (Home tela 3), vou até compartilhar aqui no Google Meet, e você pode me ajudar nisso. Eu disse que nessa parte você é bem melhor que a gente... (TIC 1).

Aqui é a home é a apresentação dos vídeos ok... aparentemente não tem muito segredo nessa parte. No start (menu tela 3) aqui é alguma coisa que a gente pode mudar isso (TIC 1).

Aí pode ficar com vocês é ilustração que eu quis apresentar como seria a apresentação inicial do aplicativo (Pesquisador).

Perfeito! isso que a gente consegue mudar um pouco. Essa aqui é minha dúvida. No “inserir o novo projeto de reabilitação psicossocial” (tela 5), os projetos de reabilitação psicossocial são pacientes? (TIC 1).

Sim, são os pacientes. Mas no sentido, TIC 1, de que como por exemplo, ao ser feito um projeto de reabilitação psicossocial, ao se “inserir o novo projeto de reabilitação psicossocial” (tela 5), o profissional de saúde mental, vai inserindo esse projeto que é sobre o paciente, e a ideia é que vai aparecendo, o paciente, o nome dele e o projeto que estão em construção ou finalizado (Pesquisador).

Tá! Vamos ver se eu entendi! Então o paciente representa um projeto de reabilitação psicossocial? (TIC 1).

Isso é um projeto de reabilitação psicossocial de cada paciente, o exemplo do TIC 2 (tela 6), pois o esqueleto do “App projeto de reabilitação psicossocial” está entre a página 8 a 14, então cada paciente vai ter um projeto de reabilitação psicossocial, não sei se ficou agora claro?! (Pesquisador).

Porque é isso que eu não estava entendendo... Porque tinha os pacientes, aqui, tela 5, então eu achava que o profissional criava um projeto, e ele inseria por exemplo 2 ou 3 pacientes nesse projeto (TIC 1).

| **Perfil do usuário/profissional de saúde mental/Técnico de Referência** |
| --- |
| Nome: |
| Sexo: ( ) Feminino ( ) Masculino |
| Raça: ( ) Branca ( ) Preta ( ) Parda ( ) Amarela ( ) Indígena |
| CPF: |
| Profissão: ( )Enfermeiro ( )Fisioterapeuta ( )Psicólogo ( ) Profissional de Educação Física ( )Nutricionista ( )Odontólogo ( )Farmacêutico ( )Biomédico ( )Fonoaudiólogo |
| ( ) Pedagogo ( ) Musicoterapeuta ( ) Médico ( ) Médico Psiquiatra ( ) Psicólogo Clínico |
| ( ) Psicólogo de Saúde Mental ( ) Enfermeiro Psiquiátrico/Saúde Mental ( ) Assistente-social |
| Contatos: |
| Telefone: WhatsApp: Email: |
| Endereço Pessoal: |
| Rua: Nº Bairro: Estado País |
| Endereço Profissional: |
| Rua: Nº Bairro: Estado País |
| Local de Trabalho: |
| ( ) CAPS I |
| ( ) CAPS II |
| ( ) CAPS III |
| ( ) CAPS Ad I |
| ( ) CAPS Ad II |
| ( ) CAPS AD III |
| ( ) Leitos de Saúde Mental em Hospitais Gerais |
| ( ) Hospital – Dia |
| ( ) Consultório na Rua |
| ( ) Equipe Especializada em Saúde Mental |

| **TRANSCRIÇÃO DAS FALAS DOS PARTICIPANTES DA REUNIÃO 26/05/2024 (Encontro Extraoficial).** |
| --- |

Boa noite, Pesquisador, tenho uma dúvida do sistema. Esses tópicos do projeto (tela 7) são fixos (sempre serão esse independente do usuário/projeto) ou eles podem ser alterados. O profissional pode criar ou remover algum? (TIC 1).

Ein!, TIC 1, tudo bem? Olha, TIC 1, esses tópicos sempre serão fixos e cíclicos. O profissional não pode remover, mas ele pode voltar sempre nele numa forma de círculo, não sei se entendeu? (Pesquisador).

Deixa eu ver se eu entendi. Então, é como se fosse uma linha do tempo. Você vai, por exemplo, adicionando coisas como se fosse a evolução do paciente. E aí depois você pode voltar num tópico pra ver meio que o que você colocou no passado. Foi isso? (TIC 1).

Não só voltar o que eu coloquei no passado, isso mesmo, como estratégias de linha de tempo, mas eu também posso editar, colocar novas informações (Pesquisador).

Entendo, entendi, certo. E eu também estava pensando, Pesquisador. Não sei se sobre sua necessidade, mas cada tópico é dado, do paciente, metas, intervenção, é meio que um campo totalmente editável, como se fosse um Word. Você consegue colocar textos, você consegue colocar imagem. Se precisar colocar print, se precisar tirar foto de um documento, coloque. Nessa ideia, mais ou menos, os tópicos. Ou cada tópico tem uma coisinha específica? (TIC 1).

É isso mesmo, TIC 1, tá?! Mas tenta fazer o documento do Word, mas dentro da janela, não é? Não vai ser o Word igual o que a gente vê no PC, né? É isso mesmo, dessa forma (Pesquisador).

Certo, Pesquisador, obrigado (TIC 1).

| **TRANSCRIÇÃO DAS FALAS DOS PARTICIPANTES DA REUNIÃO 05/06/2024 (Encontro Extraoficial).** |
| --- |

**Figura 1:** Desing Gráficos ilustrativo que apresenta o projeto gráfico do “App projeto de reabilitação Psicossocial”


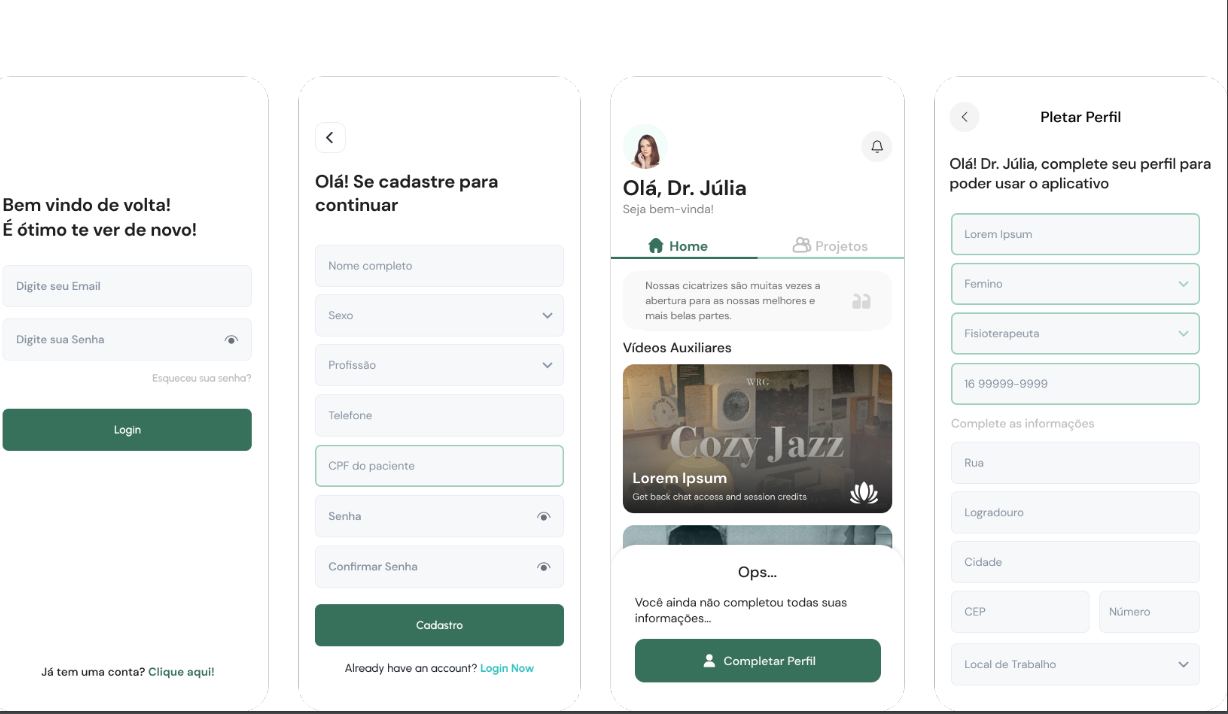


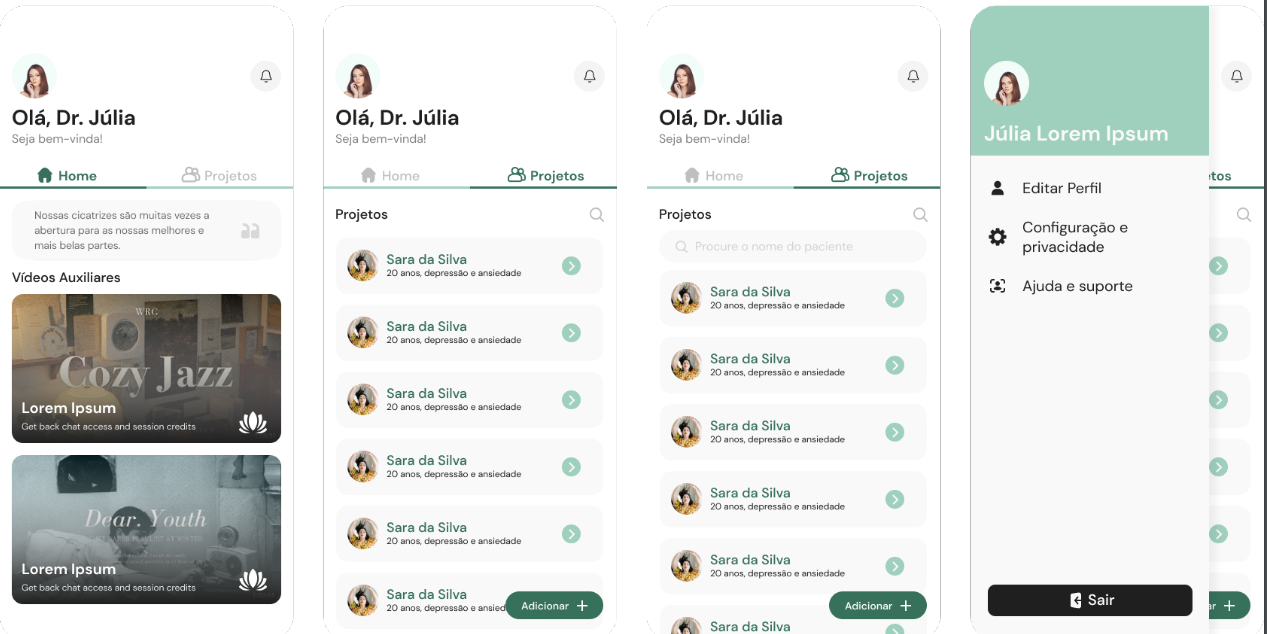


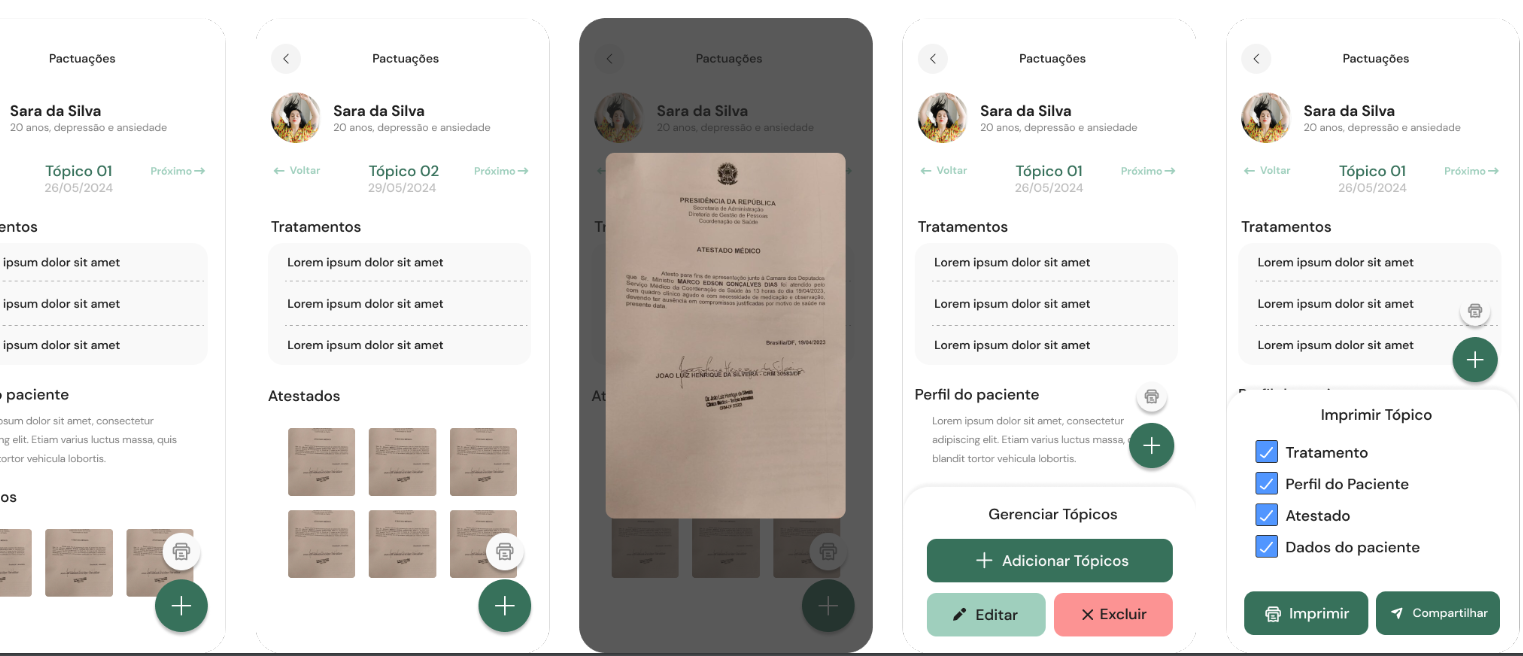


**Fonte:** Figma (2024).

O objetivo da reunião é apresentar o design gráfico do "App projeto de reabilitação psicossocial" que vai subsidiar os desenvolvedores na sua construção. Para isso e para o desenvolvimento de fato, a gente precisa do feedback seu, se atende às suas necessidades, se achou que tá bom, se você quer que altere alguma coisa. Essa aqui, eu vou começar a apresentar, essa aqui é a tela de login de cadastro. Tela de login bem normal, simples, a tela de cadastro continua naquilo que o profissional da saúde só vai conseguir se cadastrar a partir do momento que ele colocar UM CPF DO PACIENTE (Grifo Nosso), e aí ele já vincula ele ao paciente. Pensei nessa questão porque talvez a gente limite os profissionais da saúde, você só pode se cadastrar se você já tiver um profissional, ou um paciente, aliás. Aqui tem a continuação, o profissional da saúde que se cadastrar vai ter esses dados, só que ele também vai ter endereço, ele vai ter outras questões. E aí é um formulário muito grande. Em vez de a gente colocar isso no cadastro, a gente pode realizar o cadastro dele faltando algumas informações, depois que ele entrar no aplicativo, a gente pode barrar o uso dele enquanto ele não completar o perfil ainda. Porque a gente consegue limitar isso. Em vez de a gente colocar um formulário muito grande nessa parte, a gente joga essas informações pra quando ele entrar no aplicativo (TIC 1).

Um texto corrido, ou você pode inserir fotos. Entendeu?! (TIC 1). Entendi! (Pesquisador).

E aí, quando ele completar o perfil, ele retira esse nome pra essa página pra colocar essas outras informações. Como local de trabalho, rua (...) cidade. Essa aqui ficaria a parte do cadastro (TIC 1)

Tá tranquilo, deixa eu só entender. Eu queria que tivesse um link aqui antes no cadastro pra gente colocar pra ele já saber da política de proteção e privacidade de dados. Isso, pra ele já tá... E aqui dizendo que ele tá ciente, né?! dessa política... E uma coisa que eu tô pensando aqui, TIC 1, é que nesse momento não ficou muito bom, esse cadastro seu, mas a gente colocar o CPF do paciente, eu acho que, eu não sei se vai ser, uma boa, se não seria ruim aqui nessa tela do usuário do app, aqui, a gente exigir o CPF do paciente? (Pesquisador).

Exato, ele não vai conseguir usar o aplicativo sem determinar o cadastro dele, porque as outras partes do aplicativo precisam dos dados que ele ainda não colocou (TIC 1).

Mas ok, eu acho que ainda dá, mas como, por exemplo, ele colocou esse CPF e já vai direto lá para o primeiro projeto dele? (Pesquisador).

Eu talvez não vincularia o projeto, porque talvez não tenha nenhum cadastrado ainda, mas já vincularia o paciente. Aí a gente entrava naquilo. Como eu sou o técnico de referência desse paciente, sabe?! Aí eu controlo se eu quero mostrar a visibilidade dele, o que eu quero fazer com ele. Aí já vincularia o paciente junto com o profissional da saúde (TIC 1).

Tá, só que aqui, como por exemplo, se ninguém tiver ainda o primeiro contato do paciente, quem que vai ser o primeiro que vai inserir esse CPF? como, por exemplo, se ninguém ainda tiver inserido porque se cada pessoa for cadastrar é condicionado a um CPF se ninguém tiver ainda inserindo o CPF do primeiro paciente, né!!! e aí a gente pode ter um problema de não conseguir o cadastro... (Pesquisador).

Pode deixar isso aí. Para ele depois fazer, né, talvez (TIC 2). Segundo a tela, talvez? (TIC 1).

É. Mas, tipo assim, você quer cadastrar o CPF, do paciente, sim ou não?! Senão, ele pode fazer posterior, né, também. Porque aí... Não apareceria nada, né?!!!? (TIC 1).

Também essa possibilidade. Porque se for ficar para posterior, então é melhor deixar... para depois no projeto, não é? (Pesquisador).

Hora de se criar o projeto?! (TIC 1).

Aqui eu também não sei responder, nesse momento como seria... só trouxe essa questão, porque se não tiver o primeiro CPF, ninguém tiver colocado, mas quem que vai colocar?! (Pesquisador).

É, por exemplo, a gente pode colocar isso manual, né? Via, por exemplo, banco de dados. Só que... O que eu penso, assim, é a questão de segurança, sabe?! Na hora de ele criar um projeto. Ele vai precisar digitar alguma coisa. Como por exemplo, eu sou um psicólogo, eu não posso visualizar todos os pacientes e escolher qual eu quero. Porque aí abre brecha pra um mal-intencionamento, né?! Abre brecha para eu ter dados de pacientes, eu conseguir alterar e visualizar pacientes que não são meus de fato. O sistema não pode permitir isso. Eu acho que a gente pode manter nessa questão do CPF do paciente por ser uma chave única, só que talvez não aqui. Mas eu acho interessante que a gente mantenha essa questão (TIC 1).

Eu entendi, eu acho legal, sim, do CPF. Isso ficou uma ideia muito boa, tá?! Mas por outro lado, há um problema que tá dando por escolher que ele fique no começo, porque ele não vai conseguir acessar, por que como que vai ser os pacientes índices?! (Pesquisador).

Entendi. Agora é pra essa parte mesmo, pra gente discutir as ideias de como do fluxo do sistema, que é essa questão das telinhas. Eu tô fazendo agora as anotações aqui também, que aí depois a gente corrige o design. Aí agora tem a parte do menu principal. Essa aqui é a parte do menu principal, que é a Home. Essas questões aqui, esses cardizinhos, eles são vídeos. Esse Lorem Ipsum é uma coisa que a gente coloca por ser um texto aleatório, só para mostrar que ali vai ter um texto, só que ainda não foi estipulado, então a gente não sabe o que mostrar, a gente coloca isso só por questão de visualização (Pesquisador).

Tá perfeito (Pesquisador).

(...) Os vídeos, essa frase que eu coloquei também para ter alguma coisa, mas tudo pode ser alterado, pode visualizar depois naquele relatório. Essa aqui é a parte da home então (TIC 1).

Entendi (Pesquisador).

Quando ele troca de abinha ele mexe para o lado, e aí já fica parte dos projetos, aí pode visualizar quais projetos ele tem com a idade o que ela está sendo tratada tudo isso aqui também pode ser mudado sem problema nenhum (TIC 1).

Eu achei que isso ficou muito legal, isso aqui é tudo é tudo! na home né!.. ele só mudar... (TIC 1).

Exato só mudar ele só vai passar ele passa pro lado e ele vai ter acesso a home junto com os vídeos, passou pro lado, vai ter acesso aos projetos. Aí, dá liberdade pro usuário, o que que ele quer fazer... Aqui, a próxima parte é a parte do filtro. Imagina que ele clicou nesse botão, ele clicou no botão de filtro, a gente abre um campo pra ele digitar o nome do paciente. E as vezes um, eu sou um profissional de saúde que eu tenho vários projetos, essa lista ela vai se estender, então ela vai passar da tela do aplicativo, ele vai ter que rolar pra baixo pra visualizar. Aí aqui com filtro fica mais fácil (TIC 1).

Entendi. Deixa só. Volta lá na home. Depois ficou muito legal. Vamos só para encurtar. Vamos deixar só um vídeo. Porque aí a gente não deixa muita informação. Um vídeo. E aqui eu vou pensar, ficou muito legal essa frase, eu vou pensar numa frase pra gente colocar aqui, tá?! (Pesquisador).

Beleza (TIC 1).

Ficou ótimo. Então, essa parte assim, essa ideia... ficou muito legal. É isso mesmo. Tá, pode continuar, ficou muito bom. (Pesquisador).

Essa parte do projeto, tem alguma ressalta? (TIC 2).

Não... ficou... (Pesquisador).

Você acha tudo bem a gente mostrar aqui o que tá sendo tratado?! (TIC 1). Sim, tá ótima a ideia. E o local que mostra o nome do paciente, idade e diagnóstico está muito bom (Pesquisador).

Essa telinha aqui, Pesquisador, ela chama Drawer. O que é o Drawer? O Drawer é quando a pessoa ou o profissional de saúde clica na foto dele. E aí essa tela aparece, ela rola verticalmente e aparece as informações do perfil do usuário. Se ele quer editar perfil, se ele quer ver configurações de privacidade, ajuda e suporte, se ele quer sair do aplicativo. Então é como se fosse um submenu. Esse submenu fica escondido, ele fica aqui. A pessoa bateu aqui, clicou, esse submenu abre. Ela clicou fora dessa parte, ele fecha. Funciona nesse estilinho. Então, a questão do menu principal é essa. É o resumo dos projetos. E a home. (TIC 1).

Uhum... Ficou incrível, como eu falei até agora. Muito bom (Pesquisador).

Então, aí a gente vai aqui para a parte mais complexa e ampla do app, que a construção do projeto de reabilitação psicossocial. Vou dar um zoom aqui. Quando um profissional de saúde clica no projeto, é isso que ele vai abrir. São todos aqueles tópicos do projeto de reabilitação psicossocial, tela 7 do protótipo do "App projeto de reabilitação psicossocial" construído por você (TIC 1).

E onde que ele vai clicar? Pra abrir o projeto de reabilitação psicossocial?... é... (Pesquisador).

Pra ir pra essa parte? (TIC 1).

Isso, pra ir pra essa parte de novo, como se ele fosse inserir um novo projeto de reabilitação psicossocial (Pesquisador).

Aí ele vem pra cá, adicionar. vai ter esse botãozinho aqui de adicionar. Porque pensa que aqui a gente está na aba projetos... (TIC 1).

Que são os que ele já fez, né?! Aqui, quando ele conseguir clicar dentro de um projeto desse, vai aparecer todos os tópicos daquele paciente que ele vai poder editar, não é?! (Pesquisador).

Exato. Exatamente. E essa aqui é a parte inicial do projeto (TIC 1).

Mas dá para eu colocar lá naquele botão adicionar projeto? (Pesquisador).

Talvez por questão mais legítima? (TIC 1) ... Isso... (Pesquisador).

Claro. Perfeito. Então... Essa aqui é aquela partezinha que a gente tem nos tópicos do projeto. Aqui também eu pensei numa funcionalidade nova de ele já conseguir pré-visualizar quantas publicações tem nesse tópico. Pra ele não precisar clicar e ver. Aqui a gente já consegue, por exemplo, metas de cuidado e saúde mental. Ou seja, quantificar a quantidade de tópicos que estão... Aí a gente já consegue ter essa informação antes. E aqui também, como evolução do paciente é como se fosse um tópico diferente, ele fica nesse esquema do botãozinho de baixo, junto com o adicionar (TIC 1).

Legal! (Pesquisador).

Aqui a gente consegue diferenciar isso. Legal, legal. Não, tá ótimo. Essa ficou legal (Pesquisador).

Aqui é um exemplo de um detalhe do tópico, que ele clicou e abriu. Esse aqui é um exemplo de pactuações. Vai ter informação do paciente, a gente continua com isso. E aí a gente tem o tópico 1, como se tivesse vários tópicos cadastrados. A gente tem a opção de voltar e ir para o próximo tópico. E aqui a gente tem as informações do tópico. O que eu pensei em informações? A gente tem três tipos diferentes. Essa que a gente pode tratar meio que pelas bolinhas, sabe? Como a gente faz um documento e a gente tem aqueles tópicos dentro um do outro. A gente tem um texto corrido e a gente tem a opção de cadastrar imagens. Aí na hora de cadastrar pode aparecer essas três questões para ver o que você quer cadastrar. Às vezes você quer cadastrar só um texto corrido, às vezes você quer cadastrar só uma imagem, às vezes você quer só essas partes separadas. Talvez essas partes separadas sejam bem interessantes para dados do paciente. Você cadastrar nome... que é alterar alguma coisa. Aí nessa questão do detalhe do tópico, eu pensei nessas três categorias (TIC 1).

Repete de novo pra mim, do detalhe das três categorias (Pesquisador).

Aqui de novo. Ele clicou em Pactuações, ele veio pra essa tela. Aqui, essa parte ela é fixa, essa parte não muda. Daqui pra baixo, essa parte irá mudar. Por quê? Aqui a gente está visualizando os detalhes do tópico 1. Então, se a gente for próximo, isso vai mudar porque vai ser os detalhes do tópico 2. É como aquela questão da gente cadastrar a evolução do paciente. Aqui ele está visualizando a evolução do paciente. Entendeu? E aí, o que você pode inserir na visualização do paciente? Você pode inserir esse tipo aqui de tópico, que são informações separadas. Você pode ou inserir (TIC 1).

Entendi. (Investigador).

Esse aqui é o tipo de coisa que você pode inserir no tópico do paciente (TIC 1).

Eu achei legal essa lógica, só que tem lá, como por exemplo, lembra que nos dados do paciente a gente tem um texto, umas informações já padrão que foi validado e prototipado anteriormente, lembra?! (Pesquisador).

Sim, aí os dados do paciente, por exemplo, a gente pode tratar diferente porque a gente vai meio que... são dados mais fixos. Isso aqui já tem o dado da pacutuação... por conta disso, por ser algo mais alterável, sabe?! (...) Pode ser só informações meio que soltas e mais fotos. Aqui não teve a necessidade de escrever um texto corrido, por exemplo. Isso aqui são meio que ideias para a gente deixar essas questões mais personalizáveis. Às vezes, na agenda de estudo de caso, você não quer colocar nenhuma informação solta e nenhuma foto. Na agenda de estudo, você quer colocar só um texto corrido (TIC 1).

Na agenda de estudo, aqui é só para... É, não ia precisar, porque era só para lembrar o profissional da pauta da reunião e o dia e a hora. Só que é essa questão também que você falou, porque pode ser para vários pacientes, não é só uma organização, mas é uma agenda para ele organizar as reuniões sobre os pacientes com a equipe. Eu gostei dessa ideia sua, sim, da forma que você colocou aqui de exemplificar, acho válido. Só que a gente só precisa se atentar pelo esqueleto do projeto de reabilitação psicossocial. Deixa eu, como por exemplo, Eu tô tentando entrar lá no... naquele negocinho das ideias que eu fiz, como é porque, por exemplo, no dado do paciente. Eu não tô conseguindo visualizar como que vai entrar isso daqui em todos os passos. Como, por exemplo, nos dados do paciente, ele vai ter, tipo, aquele checklist, igual que fez um cadastro do profissional entrar. Você lembra? Não sei se vocês estão entendendo a minha dúvida.... (Pesquisador).

Você quer que eu abra aqui o link do protótipo do "App projeto de reabilitação psicossocial" do Marvel? (TIC 1).

Ah, eu queria (...), mas até aqui ficou incrível. É isso mesmo!!!. É que da evolução esses tópicos os ícones... Ficou bem usual para ele entrar (Pesquisador).

É que a gente pensa nisso na hora da prototipação. E sobre essas questões de que o usuário não tem que aprender nada sobre o sistema, tem que ser intuitivo. De ele saber que quando ele aperta pra avançar no canto superior tem um botãozinho pra voltar.... (TIC 1). Ein TIC 1. Vem aqui no inserir, inserir. Vai aqui no dado do paciente (TIC 1 mostra no Marvel a tela 6 do protótipo do "app projeto de reabilitação psicossocial"), é isso. Entendeu? Olha, no dado do paciente... a gente já tem as informações estruturadas que vai colocar, como o nome, a idade, aí que você colocou o CPF, né, que eu coloquei também que pode deixar, que é o CNS (Cartão Nacional de Saúde) que eu uso, o endereço e telefone do paciente, a profissão dele, a renda, aí tem essas informações. Essa do diagnóstico, então a gente já tem também. Essas são tópicos dentro dele, subtópicos desse diagnóstico, que tem como, por exemplo, a história do caso do paciente e os seus diagnósticos. E são aqui. E aqui, olha, e aqui que entra, volta lá, o negócio aqui tem os problemas que ele vai identificar porque o projeto ele tem um objetivo é a gente resolver os problemas dos pacientes psiquiátricos, orientados pela teoria da reabilitação psicossocial (Pesquisador).

Continuando aqui, essa próxima etapa é sobre fotos. Eu acho que a gente pode manter também essa questão, não é? (TIC 1).

Eu acho que de manter essa função da inserção da imagem, é legal manter lá na evolução. Entendeu? Só lá na evolução. Se o usuário quer inserir... ou melhor... também lá nos dados do paciente. É nos dados do paciente, lembra, depois que a gente inseriu. Pode voltar. Então aqui, entendi... Depois que a gente inseriu tudo isso daqui, pode colocar um ícone lá nos dados do paciente no final para você colocar imagens, né?! Anexar imagem, arquivos, não sei se você entendeu, né? Porque isso será importante para o profissional na coleta de dados do paciente (Pesquisador).

Essa foi minha ideia, Pesquisador. A minha ideia era assim. Vamos apoiar que a gente crie um “novo projeto”, um novo tópico do projeto. Certo? Você quer adicionar uma pactuação. Ao adicionar uma pactuação, você terá vários tipos de formas diferentes para inserir dados. Então, por exemplo, estou adicionando uma pactuação. Eu quero inserir um dado do tipo de imagem. Então, eu posso fazer isso nos dados do paciente, em metas, em pactuações, em agendas. Eu acho que a gente não precisa se limitar, por exemplo, fotos só em dados dos pacientes, sabe? Quem estiver criando, a gente dá liberdade para quem estiver criando, insira o dado que ele acha necessário para a criação. Não sei se você concorda comigo (TIC 1).

Eu acho legal esse recurso sim, eu acho que é bom.... e é personalizado.... deixar essa liberdade do usuário do aplicativo. Só que existe dentro dessa liberdade, existe o que eu te disse, que existe o projeto que é o método estruturado, que a gente não pode perder, que é essa etapa. E aí, dentro desse projeto, o inserir essas imagens não faz sentido dentro, dentro, por exemplo, da meta, da intervenção. (Pesquisador).

Já existe como se fosse uma pré-estrutura... (TIC 1).

Isso. O que onde que vai ter, igual por exemplo aqui, agora, nas pactuações existem aqui como, por exemplo, algumas imagens são iguais para.... aqui lá nos dados do paciente, no final, sobre aqui, pode ser nos dados do paciente, ou... Então, assim, seria lá... mais... Vai lá nos diagnósticos e... Diagnósticos em saúde mental, isso. Então, aqui é onde o paciente... Onde, na saúde, o paciente vai ter imagens para inserir. Será mais declarações que ele vai trazer, laudos. Então, o sentido disso ser válido, será mais nessa parte do diagnóstico situacional em saúde mental, em que o profissional vai consultar para ele poder saber informações desse paciente. Entendeu? Então, assim, é por isso que estou falando sobre a utilidade aonde vai ser. Porque aqui são informações que são mais intencionais e orientadas para ajudar esse paciente em sua reabilitação psicossocial. Então, o que eu quero dizer é que não vai ter... não é nenhuma imagem que vai fazer sentido... (Pesquisador).

Por exemplo. Uma coisa que você falou é que nesse diagnóstico existem alguns tipos de imagens, e eu não posso decidir colocar uma imagem no diagnóstico sem ser esse tipo (TIC 1).

Por exemplo, é que o paciente ou a família falou que ele é esquizofrênico. Aí ele mostra um laudo que tem o diagnóstico dele... e aí faz sentido. O profissional pode colocar (Pesquisador).

(...) Você consegue mandar pra gente isso? Por exemplo, quais fotos fazem sentido para o usuário enviar no diagnóstico? O que a gente pode fazer, por exemplo, no horário do cadastro... botãozinho fazendo inserir laudo, inserir, não sei, atestado, alguma coisa assim, sabe?! (TIC 1).

É isso, é aquilo que você está entendendo. Então aqui, mas assim, nesse cadastro do Diagnóstico, você pode colocar aqui, não tem muito, é laudo, coloca, inserir imagem de laudo, declaração, deixa eu ver, laudo (Pesquisador).

(...) A gente anota. Isso (TIC 1).

Ei, travou o Meet. Deixa-me anotar aqui que eu envio para vocês. Calma aí. Então vou colocar isso. Inserir imagem de laudo, declaração. Ein... tá aí bicho... aqui começa a fazer sentido... olha no que vocês trouxeram... volta lá para o tópico.... de novo... é lá nos esboços do protótipo Marvel. Então olha, aqui no diagnóstico que escrevi, eu vejo que para imagem que vai dar, vai ser inserir laudos, declarações e a gente pode colocar outras imagens. Eu vou mandar isso para vocês. Aí vamos lá na meta.. não tem não tem sentido é de colocar imagem... (Pesquisador).

Entendi (TIC 1).

Vamos lá pra intervenção. Intervenção (Pesquisador).

Tá (TIC 1).

Uma intervenção... Então... Então também na intervenção não vejo a necessidade de fixação de imagens... Agora vamos lá na pactuação... Pode ir na próxima. Isso, agora aqui na pactuação... Eu vejo... que pode ser necessário... inserir a imagem, a ata da reunião com as pactuações. Você entendeu? Olha, aqui está realmente, eles vão precisar de uma ata dessa reunião... (Pesquisador).

Uma dúvida minha. Essas intervenções de saúde mental podem ocorrer no caso de um paciente, por exemplo, ele foi internado involuntariamente? (TIC 1).

Pode ocorrer dos profissionais... olha, volta lá no que eu fiz da intervenção (tela 11 do protótipo). Pode ser que uma intervenção, possa ser que o profissional observe que o paciente chegou e ele encaminhou pra urgência, encaminhou pra ele ser internado. Entendeu? (Pesquisador).

Entendo (TIC 1).

Então, as disciplinas, elas vão. dependendo dos problemas e da situação do paciente. Só que aí o Projeto de Reabilitação Psicossocial, ele não se limita só ao que está acontecendo, mas também ao macro, no estabelecimento de metas e intervenções para a reabilitação psicossocial e qualidade de vida do paciente. Então, uma meta a longo prazo seria socialização desse paciente na comunidade. Então, qual era o problema? Paciente com déficit de socialização. Uma intervenção seria conversar com o paciente no centro comunitário ou na academia de saúde, aí o responsável vai ser quem vai ajudar nisso, ou o próprio técnico de referência, que é o que está manipulando o aplicativo, o projeto, o usuário desse aplicativo que é com o projeto do paciente, ou poderia ser uma outra pessoa da equipe, ou também poderia ser uma pessoa que foi treinada lá nessa academia de saúde, tá?! E aqui o prazo que ele teria para fazer isso, entendeu? Como, por exemplo... (Pesquisador).

Entendo, entendi (TIC 1).

E o prazo... muitas vezes não vai ser um dado fechado... mas assim ele poderia ser "começar em agosto de 20224"... não sei se você entendeu né... (Pesquisador). Entendi tá... (TIC 1). E assim, essa reunião a gente vai organizando, então a gente vai construindo até uma coisa redonda, porque muitas coisas que são muito diferentes fazem que a gente faz no papel. Então não entendo que mesmo eu tendo essa estrutura, de forma idealizando, aqui a gente está trabalhando no mundo das ideias, vocês estão ajudando a dar realidade e tornar isso concreto e realista. Porque o objetivo é que as pessoas consigam tornar o projeto de reabilitação psicossocial mais fácil e menos complicado do que o papel, porque se ele for dessa forma o aplicativo vai decorar, vai ser uma ferramenta de ajuda ao profissional de saúde mental, pois esse vai perceber: Veia! como ele me ajuda, como melhora a comunicação, a interação, como que com esse aplicativo eu consigo trazer resultados para o meu para o meu paciente, deixe o meu serviço mais dinâmico. E aí? (Pesquisador).

Entendo, entendi (TIC 1 e TIC 2).

Cê acha que em avaliação a necessidade de inserir imagens? (TIC 1).

Tem sim e a gente pode colocar para inserir ata da reunião (Pesquisador).

Ata da reunião? Toda reunião, no final, ela é feita uma ata? (TIC 1).

Geralmente, sim. É uma forma da gente corresponsabilizar. Eu achei que isso foi o que vocês trouxeram a ideia de ter uma imagem. De eu pensar, olha, quando vai gerar a imagem? E aí é muito legal, porque aí geralmente as reuniões têm que ter ata. E é uma forma de corresponsabilizar, porque aqui tá dando partes que as pessoas vão fazer então, ter o documento fica mais fácil, né?... Não sei se fez sentido... (Pesquisador).

Isso também da imagem é uma coisa assim, que no início a gente não pensa, a gente pensa no decorrer. Por exemplo, você não viu uma necessidade disso no início, eu vi uma outra necessidade disso da minha parte, aí agora a gente junta os dois mundos em algo que faz sentido. Isso também vai acontecer durante o desenvolvimento do "App projeto de reabilitação psicossocial" (TIC 1).

E o que vai facilitar, né?! (Pesquisador).

Voltando para as outras telas, então a questão da imagem acho que a gente finalizou, né?!... (TIC 1).

Sim! (Pesquisador).

A questão de quais tópicos serão necessários. Essa aqui, Pesquisador, o que é esse menuzinho aqui? Esse menuzinho é o menu na parte inferior da tela. Quando o usuário clicou em mais, o que significa? Ele pode adicionar um novo tópico. Então aqui ele tá no tópico 1, só que ele quer gerenciar esse tópico porque ele digitou alguma coisa errada. Então ele clica nesse + e vai abrir esse menuzinho pra ele. Se ele quiser adicionar um tópico, ele quiser editar esse tópico ou se ele quiser excluir esse tópico. Isso aqui é meio genérico, porque, por exemplo, a exclusão e a edição não serão todos os usuários que possam fazer isso. Aqui a gente está mostrando como se você fosse o técnico de referência desse paciente. Isso. E aí fica nessa parte de gerenciamento. A mesma coisa na parte de impressão, porque é mais fácil de visualizar para o usuário. Acho que você já está vendendo o tópico. Aqui, imprima o tópico, e você vai para outra página, e para outra página mostrar essas mesmas informações do tópico. A gente pode tratar do jeito que está acontecendo aqui. Se você clicar em imprimir, que é um botãozinho que fica fixo no canto inferior da tela, ele mostra as informações que você deseja imprimir. E aí, o que são essas informações aqui? São as que você digitou, por exemplo. Vou até abrir aqui para ficar mais fácil de visualizar. você quer imprimir a avaliação programada do projeto desse paciente. É isso que você quer imprimir. Só que você quer voltar a fazer observações invisíveis, porque talvez não faça sentido pra quem você está mostrando essas informações. E aí na hora de imprimir, o que ele vai fazer? Ele vai mostrar pra você saber se você quer todas essas informações, entendeu? Essa aqui foi a minha ideia fazendo. Você pode dizer o que deseja ou não compartilhar ou imprimir (TIC 1).

Perfeito. E esse compartilhar, aí eu vou poder compartilhar por e-mail, WhatsApp?! (Pesquisador).

Sim (TIC 1).

Esse aqui, ele fica naquele mesmo esquema, por exemplo, quando você baixa alguma coisa que está no navegador, você baixa o PDF. Você clicou nele, clicou em compartilhar. E isso é o próprio celular que já faz. Ele abre uma aba abaixo e aí tem lá WhatsApp, tem Telegram, se você tiver, e-mail, Facebook... aí ele próprio direto. Nesse compartilhar, eu penso especificamente em gerar um PDF disso. É ótimo! (Pesquisador).

E aí nesse imprimir, também, os dois vão gerar PDF, só que o compartilhar já vai para uma aba específica do celular. A impressão já vai para uma outra, porque talvez isso possa estar acontecendo num computador, ou o próprio celular pode estar conectado a uma impressora (TIC 1).

É isso mesmo (Pesquisador).

É uma parte da evolução do paciente, que aí eu acho que é uma parte mais diferenciada. Aqui vai ter como se fosse uma timeline mostrando os dias das publicações. Por exemplo, segunda-feira, dia 16, há esses tópicos que a doutora Júlia (fictício) fez sobre a evolução do paciente. E também talvez possa existir mais, porque ela começou, só que foi pra um outro. Então eu acho interessante manter-se assim, no estilo meio de rede social, porque aí a gente consegue ver quem começou a evolução desse paciente. Porque se ela começou e foi pra um outro, talvez possa ter também as anotações do outro. E ela consegue visualizar tudo, como se ela conseguisse visualizar a timeline inteira dele (TIC 1).

Legal, e isso era o sentido mesmo da evolução, só que assim, nessa, lembre-se de que aqui não pode ser uma mistura de evolução de todos os pacientes, só desse paciente específico, é isso?! Tá claro? (Pesquisador).

Por exemplo, a gente tem o exemplo da Sarah, que é uma paciente... (TIC 1).

A Sara, isso, aí que então, por exemplo, se todos...Isso... visualizar de todo o mundo que teve e se fez evolução que tem o acesso que pode ter o acesso da Sara não é.?! (Pesquisador).

Exatamente! E aqui a gente clicou em adicionar e, por exemplo, o que está marcado em escuro aqui, figura 1, você está vendo a evolução desse dia. Então, talvez na segunda, quando o projeto começou, só houve a necessidade de três registros de evolução. Portanto, a gente pode gerenciar isso, a gente não precisa mostrar tudo. A gente pode mostrar por sessões, por avaliações, não sei como é que funciona essa questão de quando a evolução é registrada. Mas a gente não precisa mostrar tudo, a gente pode dividir isso (TIC 1).

Pode dividir ou então, do jeito que tá, você deixou na sua linha do tempo e ficar também o texto corrido por ordem cronológica, da primeira informação e a segunda, de forma que a pessoa consegue visualizar rolando a barra não sei se eu acho que era isso o que você está propondo, também? (Pesquisador).

É como se fosse... aqui é como se fosse realmente rede social. Você está vendendo o perfil da pessoa, por exemplo, no Facebook. Você está vendendo o perfil da pessoa, por exemplo, no Facebook. Aqui não é só a pessoa que coloca, não é o paciente que coloca, são os profissionais da saúde que colocam coisas no perfil dele. É como se fosse publicado. Você vai fazer uma publicação de evolução no perfil do paciente (TIC 1).

E é isso. Eu só acho que poderia, assim, numa sub, igual, por exemplo, tá aqui, o que a doutora Júlia colocou, de ah!!, aparece a hora, tá perfeito, é isso mesmo. Tá tranquilo, tá tranquilo, tá tranquilo. Eu só, eu acho que ficou legal, consegui entender, sim, tá, beleza, tá?! (Pesquisador).

Aparece a hora e aparece o dia (TIC 1).

Isso, tá perfeito, ficou legal (Pesquisador).

Nessa questão da evolução então e dos tópicos a gente mantém aqui (TIC 1).

Isso! Muito legal! A gente está tentando concretizar a forma realista (Pesquisador).

E alinhar essas questões também, mas acho que aí já entra mais, por exemplo, nessa questão de que uma avaliação do projeto, existe uma ata de reunião. Acho que já são questões mais técnicas, né?! (TIC 1).

Isso (Pesquisador).

Que a gente precisa alinhar. São questões mais técnicas, o que foi isso (TIC 1).

Eu vou escrever... e te enviarei depois. Eu fiz aqui, aí eu mando lá pra você no nosso grupo. Vou mandar para você, para você ver onde vai ter a imagem nos tópicos das telas do projeto de reabilitação psicossocial. Aí deixa eu te falar... tem alguma coisinha... que foi lá que eu coloquei do suporte do usuário, que eu tinha as referências em reabilitação psicossocial (Marvel, na tela 17). Não tá aparecendo, não para mim no Meet (Pesquisador).

É, pra mim tá aparecendo (TIC 2).

Tá aparecendo o que você fez... do que é o protótipo agora que é oficial do aplicativo com as interações de desenvolvimento (Pesquisador).

Então essa parte do suporte do usuário, vai ficar aqui na Ajuda e Suporte (Figura 1) (...) eu consigo imaginá-lo 100% redondinho assim do jeito. Eu imagino profissionais usando ele (TIC 1).

Ai, que bom. Você tem mais alguma dúvida ou acha que a gente já conseguiu?! (Pesquisador).

Acho que sim. Essa etapa aqui, essas etapas, na verdade, a gente está no começo. A gente não começou a desenvolvê-lo ainda. Então, essas etapas são meio que as mais importantes para as pessoas tirarem uma dúvida, quanto ao seu protótipo, e alinhar o que de fato é projeto. Então aqui, isso a gente tá muito bem, a gente tem que fazer isso mesmo, tem que conversar, tem que alinhar coisas, adicionar, remover, editar... porque é muito mais fácil a gente fazer isso agora do que a gente vê isso só acontece quando o app estiver "pronto" (TIC 1).

Eu entendi perfeitamente, acho que como por exemplo, esse alinhamento, que você está fazendo, é que quando ele, esse design agora, que já é do aplicativo, porque vai ser bem fácil para você, porque você já está aí, e é só seguir a montagem. É isso mesmo que eu estou pensando? (Pesquisador).

É isso, é como se fosse o esboço aqui. A gente está vendo como se fosse a porta de uma casa, e antes de a gente fazer ela diretamente, a gente faz seu esboço antes. A gente senta com o cliente, a gente conversa, alinha, vê o que ele precisa (TIC 1).

| **Mensagem enviado ao WhatsApp do TIC 1, na noite de 05/06/2024, com as informações solicitadas.** |
| --- |

1. Proposta a frase: “Permeando projetos de vida com sentidos e significados construídos no habitat, rede social e trabalho” (Pesquisador).

2. Proposto recurso para inserir imagens/documentos nos tópicos: Dados do Paciente, Diagnóstico Situacional em Saúde Mental (laudos, declarações, pareceres, outros documentos), Pactuações (atas de reunião), Agenda (atas de reunião) e Avaliação (atas de reunião).

- Oi TIC 1, tudo bem? Olha, eu estou enviando a mensagem que você me pediu sobre inserir a imagem. Estou colocando os documentos que são necessários conforme os tópicos e seria interessante ter um outro usuário da opção que conforme o profissional vai inserindo os documentos seja aberto para ele mais opções de inserir. Em relação ao CPF do paciente no início eu pensei, sabe o que eu pensei?! O importante do CPF, lembra que ele vai ser como você propôs, de ser o controle de quem vai ter acesso àquele paciente. Então o que eu acho que você pode pensar numa estratégia de não colocar ele no começo para inserir, mas quando no cadastro do paciente o profissional inserir o CPF do paciente ser a condicionante. É quem já tiver usado esse paciente devia mandar uma mensagem de aparecer que esse CPF precisa da autorização do outro profissional. E pra quem enviou essa mensagem de ter o recurso que esse profissional, ele vai aceitar ou recusar o acesso a esse CPF e o projeto desse paciente. Talvez com esses dados te dar uma ideia de como gerir isso, ou talvez de ter um recurso de administrativo do usuário. Então eu não sei, eu só tive essa sugestão. Me fala aí se faz sentido e se você consegue perceber um recurso da tecnologia que consiga sanar essa necessidade” (Pesquisador).

- Olá, Pesquisador. Não, certo, eu vou salvar, sim, essas ressalvas e eu vou levá-las para a próxima parte da prototipação. E sobre o CRUD, o CRUD é Create, Read, Update e Delete. É meio que um sistema por completo, sabe? Um tipo de sistema e que a maioria, assim, 99% do sistema é isso. O que é o que? O Create é você inserir dados no sistema, criar dados. Create ou Read é você ler esses dados. Update é você editar esses dados e delete é você excluir esses dados. É um tipo de sistema, sabe? É como se fosse uma arquitetura, assim. É um tipo de jeito que é criado um sistema. Por exemplo, esse sistema que a gente está fazendo é um CRUD, que são essas informações que eu te falei, porque existem outros tipos.

| **CONSTRUÇÃO DO VÍDEO ANIMADO PARA A HOME DO “App projeto de reabilitação psicossocial”** |
| --- |

Foi realizado a sintetização do conteúdo do capítulo teórico da tese especificamente no tópico “Projeto de Reabilitação Psicossocial” para ser enviado a profissional que realizará o vídeo animado.

Abaixo o material resumido:

**PROJETO DE REABILITAÇÃO PSICOSSOCIAL**

O PRP é um método sistematizado de gestão do cuidado e assistência ao paciente do serviço de saúde mental, fundamentado na teoria da Reabilitação Psicossocial (RP), e estruturado no Projeto Terapêutico Singular (PTS) e Case Management (CM), que permite ao profissional de saúde mental diagnosticar os problemas, necessidades psicossociais e demandas deste paciente, planejar e gerir o seu cuidado, intervir, mobilizar recursos na Rede de Atenção Psicossocial (RAPS) e/ou comunidade, fazer pactuações e/ou responsabilização em relação ao cuidado a ser fornecido a este paciente, monitorar, (re)avaliar e fornecer uma assistência singularizada, integral e humanística, voltada para o exercício pleno da cidadania.

Logo é importante dizer que o PRP articula e operacionaliza na prática de saúde mental, a RP, que é sua teoria de fundamento, e consiste em um processo que possibilita ao paciente do serviço de saúde mental alcançar funcionamento social, autodeterminação e direção de sua vida, independência, contratualidade, protagonismo social, cidadania, autonomia, ocupação de espaços sociais e inserção social.

Por sua vez o PTS é um plano de ação que é compartilhado e composto por um conjunto de intervenções que se guiam por meio da intencionalidade de se proporcionar um cuidado integral e humanizado ao paciente do serviço de saúde mental.

Já o CM favorece o gerenciamento do PRP, pois viabiliza e operacionaliza a gestão do PRP, por meio de sua condução, coordenação, monitoração e (re)avaliação. Permitindo que o profissional de saúde mental assuma coordenação de PRP do paciente, tendo como função geri-lo e garantir que este seja cuidado com apoio da equipe de saúde mental e qualquer outro ator social imprescindível ao PRP.

O PRP tem como objetivos cruciais:

Garantir a cidadania plena através da interlocução e (re)construção de uma estratégia global de assistência aos pacientes em saúde mental em seus cenários de produção de sentidos de vida: habitat, redes sociais e trabalho.

Criar condições para que as relações entre o paciente do serviço de saúde mental e o meio ambiente se multipliquem e ocorram de forma autônoma e as oportunidades se encontrem continuamente a sua disposição.

Identificar quais práticas e conceituações são condições necessárias para poder discutir a RP, e eleger demandas e necessidades prioritárias para implementação de intervenções.

Desenvolvimento da autonomia, independência, funcionalidade social, integração social, cidadania e qualidade de vida.

A estrutura do PRP e são divididas em quatro etapas:

1) Avaliação: é uma avaliação biopsicossocioespiritual, que possibilita o conhecimento profundo a respeito da história de vida do paciente do serviço de saúde mental, diagnósticos médicos e multiprofissionais, riscos, vulnerabilidades e dificuldades, potencialidade, desejos/valores, interesses e aspirações pessoais e profissionais, relações sociais significativas em seu território.

2) Metas Terapêuticas: precisam ser contextualizadas e situadas com base nas singularidades do usuário do serviço de saúde mental, em concordância com os pressupostos da RP e Reforma psiquiátrica.

3) Intervenções e Divisão de Responsabilidades: são eleitas ações de saúde mental a serem instituídas pela equipe de saúde mental ou qualquer outro ator social relevante para o alcance das metas terapêuticas em prol do paciente sobre cuidado (e sobre pactuação deste). É preciso estipular prazos a serem cumpridos (curto, médio e longo) e responsabilizar quem realizará as ações estabelecidas nas metas ou articulará sua realização com os dispositivos ou/e profissionais da RAPS (atores sociais).

4) Reavaliação: momento em que se discute a evolução e são realizados os devidos ajustamentos, podendo ser identificados outros problemas não contemplados na avaliação inicial, as dificuldades, falhas terapêuticas ou qualquer outra nova necessidade de cuidado surgidas, rever/ajustar intervenções e pactuações com o usuário do serviço de saúde mental e os demais atores sociais participantes.

Por isso o “App projeto de reabilitação psicossocial” facilitará os profissionais de saúde mental na condução de casos complexos e suas interações com os atores sociais necessários ao desenvolvimento do PRP em saúde mental.

Resumidamente podemos sintetizar os pressupostos do PRP que são subtendidos nos tópicos do “App projeto de reabilitação psicossocial”: 1) PRP é uma ferramenta que viabiliza a RP dos usuários do serviço de saúde mental. 2) PRP possibilita a construção da contratualidade em pacientes do serviço de saúde mental por meio do desenvolvimento de estratégias de assistência que respondem às suas demandas e necessidades biológicas, psicossociais, afetivas e socioeconômicas. 3) A estrutura interna de um PRP é determinada pelo PTS, sendo desdobrada no Avaliação, Metas Terapêuticas, Intervenções e Divisão de Responsabilidades e Reavaliação. 4) Case Management é um modelo que permite a gestão e operacionalidade do PRP pela interlocução entre trabalho em equipe, gestão de caso, mobilização dos recursos disponíveis na Rede de Atenção Psicossocial (RAPS), comunidade e (re)avaliação de casos complexos em saúde mental.

| **Encontro Oficial (12/07/2024) e extraoficial (16/07/2024)**: com objetivo de apresentar o design gráfico do “App projeto de reabilitação psicossocial” finalizado para começar o desenvolvimento “App projeto de reabilitação psicossocial”. |
| --- |

Pode começar? (TIC 1).

Pode (Pesquisador). Essa aqui é a tela de login. A tela de login, a única alteração que teve foi o link pra proteção e visibilidade de dados. Aí a gente pode manter ela aqui. E na tela de cadastro, o que teve era o concordo com aquela política de privacidade (TIC 1).

Isso (Pesquisador). Aí a gente continua aqui com... Depois eu consigo te mandar isso aqui, só essa parte de apresentação... que aí você consegue comentar também. Aí fica mais fácil (TIC 1). Tudo bem, sem problema (Pesquisador).

A parte de gênero. O cadastro fica nisso. E aí entra a primeira dúvida que eu tenho (TIC 1).

Pode falar (Pesquisador).

Qualquer pessoa vai conseguir fazer o cadastro? (TIC 1).

Qualquer pessoa como assim que você fala? (Pesquisador).

Se a gente disponibiliza esse aplicativo e a gente não coloca nada pra restringir qualquer pessoa que, por exemplo, se a gente joga esse aplicativo na Play Store, qualquer pessoa que baixa ele pode fazer o cadastro? Eu tinha pensado desde que seja aquelas profissões que eu listei, lembra lá? (TIC 1).

Não teria problema de fazer, mas eu não consigo ver uma forma de limitar nesse momento não. O que você tinha ideia de pensar? (Pesquisador).

O que eu acho que a gente pode fazer, por exemplo, existiam usuários de administradores e esses administradores validaram o cadastro do usuário. Você baixa o aplicativo, você faz o seu cadastro, só que você não consegue entrar no aplicativo até que um outro usuário, que tenha mais permissão que você, consiga validar isso (TIC 1).

Então legal, eu acho que podia criar esse administrador dos gestores do aplicativo, que seria um local pra gente, eu acho legal, acho isso importante. O administrador, sim. Necessário e válido, né? Só pra pessoa (TIC 1).

Como também a gente excluir, não é? (TIC 1).

Sim, uma conta, né? (TIC 1).

Isso. Eu acho que pra... Pro cadastro do usuário é isso. Não tem muito segredo. E aqui também não colocar muita informação porque eu acho que não convém. Eu acho que convém mais a gente continuar naquilo que a gente tava fazendo. De barrar a entrada do usuário. Caso ele ainda não tenha finalizado o cadastro dele. Porque a partir do momento que ele entrou, já vai ter telas que precisam de dados dele. Então isso vai interromper, vai atrapalhar o aplicativo (TIC 1).

Só repete, não entendi o que você falou (Pesquisador).

Antes o usuário vai entrar e ele vai ver caso seja o primeiro login dele no aplicativo, Ele vai se deparar com essa telinha e ele não vai conseguir navegar. Ele vai precisar clicar e finalizar cadastro (TIC 1).

Ah, entendi (Pesquisador).

Para continuar as informações dele, entendeu? (TIC 1).

Ficou perfeito isso (Pesquisador).

Exato. E, por exemplo, a gente não pode deixar ele entrar no aplicativo porque talvez nessa tela, nessas primeiras telas, não tenha nenhum dado que vai usar dele. Conforme ele avança, tem. Então a gente não pode deixar ele avançar sem ele ter inserido os dados dele na aplicação. Ele finaliza o cadastro dele, continua informações de todo o cadastro. Tem raça, CPF, profissão. Profissão a gente tem várias opções. Eu não sei o local de trabalho, mas profissão eu coloquei de um jeitinho diferente. Ele clica e meio que ele é redirecionado para um formulário dentro de um formulário. E ele pode marcar mais de uma também (TIC 1).

Combinado, isso mesmo (Pesquisador).

E o local de trabalho, Pesquisador, ele também pode marcar mais de um? (TIC 1).

Pode (Pesquisador).

Ele pode trabalhar mais de um lugar ao mesmo tempo? (TIC 1).

Pode. Isso, pode marcar (Pesquisador).

Entendi (TIC 1).

E aí, esse aqui não tem muito segredo, não tem nada demais. E aí a gente lida com aquilo. Aqui é a home do aplicativo. Aí continua na fase, esse suporte usuário é um card pra levar ele até lá. Aqui os vídeos. Só vamos manter um vídeo, tá? (TIC 1).

Certo (Pesquisador).

Aí depois eu coloco um card maior aqui então, como se fosse esse. A home em si ela não tem nada de mais, mas uma apresentação mesmo. Aí como a gente adicionou mais coisas, eu mudei o menu. Ele não fica mais o menu aqui em cima, ele fica o menu como se fosse do WhatsApp o usuário ele navega por baixo aqui a gente tem a aba da home que é mais essa apresentação e aqui a gente tem a parte dos projetos e a gente tem esse exemplo aqui da Sarah e aqui tem o formulário de o botão pra adicionar um projeto ai se ele clica ele vai pra adicionar, aqui já entra uma outra dúvida minha claro, pode falar clicou em adicionar projeto, eu coloquei para aquela observação que você apontou da última vez, para deixar mais explícito. Aqui é para o novo, e se, como por exemplo, a ilha de acionar já vai aparecer aqui, se ele clicasse aqui em um desses que estão listados, ele já entraria no projeto (TIC 1).

Certo (Pesquisador). Aqui ele adiciona, ó, vamos adicionar um projeto. Uma dúvida minha, projetos terão fotos? (TIC 1).

A gente não tinha pensado... você fala a foto do paciente, né? (Pesquisador).

Exato (TIC 1).

Mas a gente pode deixar, não tem problema. Fica criativo, se você acha... essa foto seria do que para identificar ou seria... em que sentido que você pensou essa foto? Só de identificar o rosto desse paciente? (Pesquisador).

Sim, é porque, assim, eu penso isso aqui como se fosse um prontuário eletrônico. A gente lida com a entrada de um novo paciente, a gente lida com o tratamento dele, e a gente só não lida com meio que o final ali do processo. Mas tem todo esse fluxo. Eu acho que só as informações, só dados, só você ler, é diferente de você visualizar. Eu acho que seria mais interessante a gente ter essa foto do paciente, só que eu também não sei se se tem algum caso que não vai dar para tirar foto (TIC 1).

Como a gente está idealizando, não tem nada demais de tirar uma foto. Vamos manter. Ficou legal, acho criativo. Pode manter, não tem problema (Pesquisador).

É que é o formulário também dele. E todos os profissionais da saúde que criam um projeto, eles viram automaticamente técnico de referência. Isso. E aí eles vão ter meio que autoridade sobre aquele projeto (TIC 1).

Muito bem (Pesquisador).

Exato. Então isso aqui tem o formulário do projeto. A gente finaliza o cadastro e sempre quando a gente altera, a gente exclui ou a gente adiciona, ele tem sempre que confirmar para mim que a gente tratar isso sabe não tomar decisões erradas dentro do aplicativo muito bom ficou muito legal aí se não volta né e se sim ele volta para essa tela e adiciona um novo aí eu vou entrar para a gente começar a falar sobre o projeto aqui começa a parte de compartilhar as informações do projeto. Você consegue entrar só um pouquinho pra mim? Se não ficar bom eu posso ir pra um outro método (TIC 1).

Tá ótimo. Agora tá legível (Pesquisador).

Certo. Aqui a gente começa nas informações. Compartilhar as informações dele, daquilo que você tem um projeto, só que você quer compartilhar comigo, que sou um outro profissional de saúde. Só que eu não tenho a necessidade de incluir coisas. Talvez a minha necessidade seja só ler o que está sendo inserido naquele projeto. Então na hora de compartilhar, você pode convidar outros técnicos, só que você tem uma opção em que você vai tratar a visibilidade desse projeto. Se os outros técnicos vão conseguir visualizar ou editar. Aí editar implica em adicionar, editar ou excluir. Aí tem meio que esses dois perfis, né? Tem o perfil de visualizador e perfil de editor (TIC 1).

E aí ele pode ir pra enviar pra outra pessoa, como que vai ser esse envio? Porque ficou perfeito esse recurso. Ah, pode ser pro WhatsApp... (Pesquisador). Esse envio, ele é meio que quando você... Por exemplo, você está em um aplicativo, você clica em compartilhar, abre essa bordinha de baixo e aí meio que já tem alguns aplicativos ali que você usa. E aí você pode subir mais ela e aí vai ter todos os aplicativos. Porque eu pensei em fazer isso através de um link. Entendeu? Você pode mandar por e-mail, por exemplo. Pode mandar para onde você quiser esse link (TIC 1).

Ficou perfeito, TIC 1. Essa ideia era isso mesmo que era (Pesquisador).

O...que a gente consegue controlar o que outros profissionais da saúde. Essa aqui é a parte do projeto. Essa parte do projeto, sinceramente, ela não mudou muito. Eu adicionei fotos onde precisava adicionar, por exemplo, dados do paciente. Aqui eu acho que já começa a foto, porque aqui a gente já começa a visualizar o paciente. Aí dá mais vida, sabe? (TIC 1).

Sim, ficou muito legal essa sugestão. Tá tudo ok até agora. Dar mais vida (Pesquisador).

E aqui a gente começa a tratar também as coisas nesse botãozinho. Esse botão, ele é o menu. Toda vez que o usuário vai clicar nele, vai ter várias opções. Ele pode compartilhar aquelas informações, e aí ele pode mandar, por exemplo, para o WhatsApp, ele pode só gerar o PDF, ele pode excluir também. Se você clicar em excluir, vai aparecer uma opçãozinha para ele excluir. E ele pode editar. Aí eu vou abrir aqui, fechar esse menuzinho e vou abrir as informações. Aqui. Essa aqui entra, eu acho que a maior dúvida, assim... (TIC 1).

Certo, pode falar (Pesquisador). Por exemplo, dados do paciente é fácil porque é aquele formulário de incluir um novo paciente, um novo projeto. Então, correto. Dados, por exemplo, dados pessoais, outras informações, não tem muito segredo. Só que, por exemplo, diagnóstico institucional em saúde mental. Aí tem essas várias telinhas e aqui tem, por exemplo, história do caso diagnóstico multiprofissional em saúde mental. A gente precisa saber o que precisa ter nisso, sabe? Porque aqui é o exemplo de um texto. Aqui é um exemplo de um texto e uma data. Sabe? O que precisa ter nisso? A gente precisava de exemplos de inserção, sabe? O que você insere nele? Você insere um texto com uma data, um horário. E aqui, por exemplo, tem um que é medicações em uso. Ml, uso da medicação, tarja. Sabe? Tem umas coisas que são mais específicas (TIC 1).

Eu entendi (Pesquisador). Por exemplo, doenças clínicas. O que a gente tem em doenças? Entendeu? (TIC 1).

Entendi. Olha, na doença você pode deixar no texto. Ou então também eu posso... Lá tem... Quando você me mandar o link eu consigo o lembrestinho pra colocar? (Pesquisador).

Sim, sim (TIC 1).

Eu posso listar para você as principais. Agora, entendi a história. Agora, a história desse caso de diagnóstico multiprofissional, aqui tem que se deixar a lacuna no texto para ele escrever. Aqui a gente não vai ter pronto, vai ficar a critério do profissional, ele vai fazer o texto corrido. Isso também, por exemplo (Pesquisador).

Isso aqui é o exemplo de uma paciente que já está tudo cadastrado dela. A gente não pode obrigar o profissional da saúde a cadastrar tudo na hora de criar um novo projeto, né? É conforme o tratamento dele (TIC 1).

Isso (Pesquisador).

E também, por exemplo, todo projeto necessariamente vai ter todas essas opções ou não? (TIC 1).

Bom, todos têm que ter essas opções (Pesquisador).

Necessariamente tem que ter todas as opções? (TIC 1).

Todas as opções. Só que vai depender progressivamente. O profissional vai construindo, né, necessários dados. Aí vem o diagnóstico, aí depois ele vai fazendo as metas, as intervenções. Pode ser, só que assim, não deixa pré-requisito. Pode ser que ele pode já ter que fazer as intervenções primeiro, pode ser que continua o fluxo seja ao contrário, mas vai ficar sobre o profissional. Entendeu? Fez sentido? (Pesquisador).

Sim, sim, fez. Conforme, como se fosse uma linha cronológica (TIC 1).

E isso fosse uma linha cronológica, mas pode ser que, como por exemplo, ele fez os dados do paciente, o diagnóstico, mas pode ser que esse paciente chegou em crise, ele conseguiu, ele já vai ter que intervir, então ele vai... a primeira coisa que ele vai fazer vai ser a intervenção, e pode ser que só na outra vez que ele vê o paciente, que ele vai ir preenchendo as outras questões, entendeu? (Pesquisador).

Entendi. Aí a gente pode deixar, por exemplo, um... Quando ele clicar, é porque meio que já tem exemplo de tudo. Por exemplo, é a pactuação. Tem exemplo de uma pactuação. Sim. Aí ele clica e abre... Isso aqui são dados que eu coloquei só pra mostrar, sabe? (TIC 1).

Mas esses dados pra mostrar foi o que a gente já tinha conversado, não é?...O responsável... isso... (Pesquisador).

É que tem uns que realmente são, é bem específico, por exemplo, é... medicação. Sabe? O que que a gente tem que colocar de uma medicação pra... qual que é o campo, sabe o que eu falo? (TIC 1).

Eu entendi. O que que a gente tem que... (Pesquisador).

Colocar, qual que é o campo? (TIC 1). Tá, a medicação, eu acho que isso já dá pra gente resolver. A medicação geralmente é o nome do medicamento, a gente vai ter o nome, é o princípio, Deixa eu só lembrar aqui o nome. A quantidade, deixa eu colocar aqui, dipirona, como por exemplo, dipirona é 500 mg 4 x ao dia. Geralmente, nessa prescrição de medicamento vem o nome, a miligrama... a...a frequência no dia...a frequência e a via...se vai ser oral...endo...intramuscular...no músculo...na veia...então, geralmente, o padrão da medicação é isso...é o nome...da medicação, que é o princípio ativo, a miligrama e a posologia, que a gente chama, que é a frequência de uso. Certo. Ou então, olha, nessa questão muito específica, ou pode deixar escrever dessa forma, das lacunas, ou deixar o texto também, não é? O texto corrido. É, o texto corrido. Pode ser (TIC 1).

E aí meio que a gente trabalha só com dois tipos de campo, né? Que são textos e imagens. Por exemplo, lá tinha tabelas. Eu estava vendo sobre tabelas. Só que ia ficar muito espremido. Eu acho que não seria bom para o usuário. E cada campo de uma tabela vira como se fosse um tópico (TIC 1).

E ficou tão legal essa criatividade do tópico (Pesquisador). Eu acho que é esse o processo. Em vez de profissão ser uma tabela e ter o dado embaixo, profissão ele vira como se fosse um tópico, sabe? E embaixo o campo que é o resultado. Mas a parte do projeto é isso. A gente tem os projetos, você consegue ir continuando aquilo. Por exemplo, dentro é porque existe várias categorias de projeto, né? Categorias aqui. Aí, por exemplo, dentro do diagnóstico, já tem várias categorias, subcategorias de diagnóstico. Aí pensa que o usuário tá nesse local. Ele tá em diagnóstico institucional em saúde mental. Dentro de diagnóstico, ele tem várias opções. Ele pode visualizar o que ele quer, ou ele pode avançar entre elas, como se fosse o fluxo também. Aí chega na última, ela sempre vai voltar. Aqui, diagnóstico. Aí continua naquilo, que ela consegue avançar. E aqui o menuzinho sempre vai ter, né? Ele sempre pode compartilhar ou gerar um PDF, ele pode excluir, editar ou adicionar. Eu acho que na parte do projeto é isso, né? (TIC 1).

Isso. Deixa eu só ver a intervenção, como que ficou. A intervenção, aqui (Pesquisador). A intervenção foi um dos casos que eu não tive muita ideia do que colocar. Tem algumas coisas lá, por exemplo, meta, prazo (TIC 1).

Isso, mas é isso, só o problema. Só que tem que lembrar que esse... Também que eu coloquei data, pra mim é que lembrar, sabe? O que precisa de data, que às vezes é uma entrada que precisa, né? Sim, isso é importante. O responsável... Deixa eu ver a intervenção. Só que assim, olha, lembra que eu te falei que esse problema é que ele vai identificar lá naquela parte no começo, lembra? Lá na avaliação. Então, porque vai ter vários problemas e pode ser também várias intervenções. Então, não pode limitar só a uma (Pesquisador).

Não, sim. Ela pode, por exemplo, ela pode adicionar mais intervenções, entendeu? (TIC 1).

Ah, entendi. Isso aqui, a gente tá exibindo o detalhe de uma intervenção. Só que ela pode adicionar mais intervenções. E vai aparecendo aqui as intervenções (Pesquisador).

Exato. E isso aqui também, isso aqui tudo são exemplos de títulos, sabe? Às vezes tem, sei lá, intervenção e o nome do local. Não sei. É nesses dados específicos que eu acho que entra mais uma parte sua do que nossa, sabe? (TIC 1).

Não, aqui olha, aqui é isso, é intervenção em saúde mental e aqui é intervenção. Ele foi colocando, talvez... Clica aqui nessa primeira exemplo dessa intervenção, quando a gente volta lá. Aqui os títulos eles não mudariam, né? (Pesquisador).

Eles mudariam o conteúdo deles (TIC 1).

Entendi (Pesquisador).

Ele poderia avançar também. Para ter um exemplo do que como seria (TIC 1).

Certo, vamos ... vamos para o próximo eu acho que tá indo bem... Que a parte do projeto é isso em várias categorias dentro de cada categoria vão ter mais categorias né e aí pode adicionando aqui dentro disso entendi. Aí aqui também tem o filtro, né? (Pesquisador).

Que às vezes se cadastrar muito, talvez seja mais fácil a pessoa filtrar pelo nome da paciente. E aqui também foi uma coisa que eu não sei se convém colocar. Isso aqui tá no projeto, aqui. Motivo inicial do cadastro. Você acha que convém a gente colocar qual que foi o motivo inicial que o projeto foi cadastrado? (TIC 1).

Isso não precisa não. Isso é desnecessário, não (Pesquisador). Beleza. Agora eu posso tirar isso. Agora vamos para a parte de evolução. Que aí é uma parte...Antes, evolução ficava nessa partezinha aqui. Só que evolução, você concorda comigo, que meio que é tão importante quanto essas categorias, todas. Então, eu acredito que fica melhor a gente tratar isso numa partezinha só do menu. Ela consegue ver o paciente e a evolução dele. E aí se você clica, você tem acesso, continuando aquilo, você tem acesso da evolução da paciente, da Sarah. Você tem o calendário de quando foi adicionada a evolução. Você também consegue navegar entre elas (TIC 1).

Entendi (Pesquisador).

E uma outra dúvida aqui. Evolução, a gente cadastra só texto? (TIC 1).

Pode ser, só texto. Eu não consigo ver outra da forma que é que você tinha pensado? (Pesquisador).

Não, era uma dúvida que eu tinha, porque eu não sei se é necessário alguma foto, Normalmente são os tipos de campo, sabe? (TIC 1).

Geralmente vai ser só o texto mesmo. O que seria bom é que aí tivesse um texto pra ele inserir, mas tivesse as sugestões do que foi feito, igual por exemplo. Lá dos principais problemas que ele teve... e as intervenções e as metas... as metas e as intervenções que foram trabalhadas até agora... para ficar como sugestão... aí o profissional, com base nisso, ele... ele iria fazer o texto dele (Pesquisador).

E nisso, na hora de criar? (TIC 1).

Na hora de criar (Pesquisador).

Certo (TIC 1).

Na hora de fazer... inserir esse quadradinho, como você tem ali da doutora Júlia, né, que houvesse as sugestões do... das intervenções, as metas que ele trouxeram (Pesquisador).

Entendi (TIC 1).

Aí ele ia ver que aquilo ia colocar, né, o texto dele (TIC 1).

Uhum, entendi (Pesquisador).

Essa aqui é uma observação que ele colocou. Mas realmente seria só texto. Mas isso aí dá pra gente colocar na hora de criar. Porque isso também eu tava com dúvida da tela de... pra gente fazer o cadastro da evolução. Eu acabei até esquecendo de colocar ela aqui. Então, sugestões de... (TIC 1).

Evolução... Sugestão de evolução em texto e que aparece aquela visualização que é tipo quando a gente faz, não vem aquelas sugestões de palavra pra gente, a gente só clica e ela aparece, ou dá o enter, mas que viesse o que ele fez até agora com o paciente, não é? principalmente as metas e os problemas, as metas e intervenções. Os problemas que ele colocou, as metas e as intervenções (Pesquisador).

O que a gente pode fazer é uma tela de cadastro, dentro dessa tela de cadastro pode enfim, os dados disso, de problemas, metas e intervenções. Porque ele pensa que toda vez que a gente mostra alguma coisa, essa alguma coisa que a gente tá mostrando, ela tem que vir de um local. Então, não tem problema a gente pegar lá de uma outra tela e trazer pra essa. A gente pode mostrar esses dados na hora que ele for fazer (TIC 1).

Isso! (Pesquisador). O cadastro de evolução vai ser bem parecido com o cadastro de projeto. Talvez aqui no início dele a gente pode colocar isso, sabe? Sugestão, alguma coisinha assim (TIC 1).

Só que é isso, mas assim, só que aí não precisa... vai aparecer esse monte de dado de novo? Ah, vai aparecer, né? Não, é importante. Eu acho que é... (Pesquisador).

Não, mas a evolução... É porque esse aqui é o formulário de criação do projeto. É o formulário de criação da evolução. Eu ainda não fiz ele. Eu estava com essas dúvidas (TIC 1).

Ou então, olha, olha que coisa legal. Eu acho... Eu não sei se é isso que você está querendo me dizer, porque quando o texto vai ser corrido para ele fazer, mas que ele consiga ter os locais que ele conseguisse visualizar o cadastro do paciente... as informações que ele colocou lá... nos diagnósticos... aquele histórico... não tem aquele primeiro lá... o diagnóstico situacional do paciente... que ele vesse as intervenções... as metas e as intervenções... mas que ele não precisasse voltar... se fosse... não sei se fez sentido para você... ver que a gente tem todos esses tópicos, né? Certo. Do paciente, mas tem o tópico macro, né? Lá como você voltou, entrou na Sarah (Pesquisador).

É, tem os dados, isso aqui são categorias, e aí a gente tem, a gente consegue visualizar isso, né? (TIC 1).

É, como assim? Aí volta lá na evolução, ele vai escrever o texto, mas você concorda que pra ele escrever esse texto, seria legal que ele conseguisse resgatar bem rápido essas informações, mas sem perder, ter que voltar lá. Igual aqui, ver que você tem uma linha do tempo das intervenções. Se tivesse uma forma dele conseguir navegar nessas informações, só visualizar o que tem para cada um daqueles topos que ele construiu até agora, do paciente, principalmente, aquilo sobre os dados, aquelas avaliações que tem dele, os problemas, as metas e intervenções, porque na evolução ele vai precisar saber disso. Entendi. Entendeu? Se tivesse um recurso que ele conseguisse (Pesquisador).

Mas você acha que ele precisaria visualizar tudo ou tem algumas coisas mais importantes? (TIC 1).

O que seria mais importante para ele ver nesse momento, olha, todos esses dados principais aqui que você está mostrando nessa tela, volta lá para o... aqui quando você clica no diagnóstico vai para aquilo lá, tudinho, não é? Isso, então esse tem que ver e assim é indispensável, entendeu? Isso. Os dados dele já não teriam tanta necessidade. Não precisaria ver. Entende? (Pesquisador).

Entendi (TIC 1).

Agora, as metas seriam importantes. Muito bem. Aí volta lá. E as intervenções. Seriam muito importantes (Pesquisador).

Então esses três? (TIC 1).

Esses três! (Pesquisador).

Pode fazer evolução (TIC 1).

Há pactuações também. Eu acho que seria legal para ele porque ele ia ver o que foi feito, quem está participando. Esses quatro tópicos seria legal de ver é lá, mas assim, só visualizar nada que ele conseguisse naquele momento ali escrever, entendeu? Seria tipo assim, olha, entende quando você clica que vai gerando aquela linha do tempo das intervenções, das evoluções que você fez? Se tivesse esse, tipo esse negócio pra ele, entendeu? Pra cada um daqueles topos que eu falei (Pesquisador).

É porque o que acontece se a gente clica aqui a gente vai redirecionar ele para algum outro local o que eu tô pensando é o modo da gente trazer isso porque é muita informação você concorda comigo? é isso que eu tô pensando porque aqui a gente já tem bastante categoria e dentro de categoria a gente tem várias (TIC 1).

Então isso seria o ideal, porque aí ele não se perderia uma das dificuldades que a gente tem (Pesquisador).

Entendi. Tá, essa aqui é uma questão. E as informações do projeto que a gente precisa trazer seria o diagnóstico, metas, intervenções e pactuação (TIC 1).

E aí, ô TIC 1, só volta lá na tela principal, lá do projeto, naquela que você me trouxe (Pesquisador).

Essa aqui? (TIC 1).

Isso... só que por outro lado a gente também não faz um resumo dessas informações. Será que aqui não existiria como hoje, quando a gente tem o recurso da inteligência artificial, de ela poder dar um resumo? (Pesquisador).

Desse paciente? Por exemplo, tem um outro projeto que a gente está fazendo, que é um projeto da Google. E ele usa a inteligência artificial. Só que, até esse momento, é gratuito. Em um futuro pode não ser, sabe? Só que eu não sei o quão fiel é a gente entregar vários dados pra ele e a gente falar, resuma isso. e o que ele vai entregar pra gente, sabe? Eu entendi. Porque tá tratando de... Você concorda comigo? Que é algo muito sério, que ele não pode errar. A IA tem que pegar o que a palavra representa, resumir e entregar o que foi resumido perfeitamente (TIC 1).

Entendi (Pesquisador).

Porque a gente tá lidando com a história de um paciente, sabe? (TIC 1).

Você está totalmente... correto...para tentar minimizar essa dor, que não conseguir ir na evolução ver esses dados, o que é que é então a gente... porque eu não consigo pensar outra forma (Pesquisador).

É porque aqui eu percebi que também tem umas coisas, né? Por exemplo, tem a avaliação do prazo e essa pessoa vai avaliar se o paciente cumpriu ou não o prazo (...)
Se aumentar muito, você concorda comigo, vai ser semana (TIC 1).

Você fala tipo assim, você vai fazer uma avaliação hoje, aí daqui dois meses vai ter uma notificação? (Pesquisador).

Sim (TIC 1).

Ou ter a notificação, lembra lá, da meta, porque aqui que é o grande feeling desse aplicativo, é a avaliação onde que vai estar interligado com todas aquelas etapas, principalmente o problema, a meta e intervenção. Então lembra lá, na meta, se ele deu um prazo e fez a intervenção e também deu o prazo, é de alertar, olha, tá chegando a meta (Pesquisador).

Por que que eu perguntei isso? A gente pode tratar esse tópico, avaliação do projeto de habilitação psicossocial, como se fosse uma mini evolução. Vamos tratar ele não a mini, é a evolução. Porque é uma evolução mais sistematizada e de fácil resgate. A minha ideia, por exemplo, forçar o usuário mês a mês, ou dois meses a dois meses, fazer a avaliação e aqui a gente consegue mostrar estatísticas disso. Entendeu? (TIC 1).

Sim (Pesquisador).

Isso aqui, essa tela pode ser uma geração de estatísticas pegando os dados de lá. Qual que é a porcentagem de meta que o paciente cumpre a curto prazo, a longo prazo, sabe? Quais foram as metas que ele conseguiu cumprir ou que ele conseguiu cumprir. Por exemplo, se o paciente coloca uma meta que ela é recorrente, só que em seis meses ele não conseguiu alcançar, a gente pode ter um controle disso e o professor de saúde talvez possa indicar pra ele aumentar o prazo pra essa meta. Eu acho que isso aqui a gente pode tratar mais com estatística (TIC 1).

Ótimo (Pesquisador).

Então essa tela seria meio que o acompanhamento. Estatísticas da evolução. E indicadores (TIC 1).

Porque, pensa comigo, a gente vai ter uma funcionalidade e essa funcionalidade só vai acontecer em dois meses, correto? Então a gente teria que, da teoria, né, claro, esperar dois meses só para testar isso, para a gente ter certeza de que está funcionando. Claro que a gente pode pensar que dois meses é um dado e a gente já consegue alterar isso. A gente consegue, por exemplo, mostrar essa alteração de hora em hora. Então a gente sabe que em hora em hora ela está funcionando. Então a gente vai aumentar esse dado para dar dois meses, entendeu? (TIC 1).

Entendi (Pesquisador).

(...) Isso aqui é uma prototipagem de alta fidelidade. Então, você consegue usar como se realmente fosse o aplicativo. O que você não consegue, por exemplo, um campo, você não consegue escrever nele, justamente por ser uma prototipagem, sabe, não tem esse tipo de função. Por exemplo, perfil, sair, você consegue ver o fluxo, como que é, (...), que é isso de você clicar e ver as opções, sabe, abrir e fechar, fazer login, olha lá, login, aí vai entrar, aí entra naquela partezinha, Aí, por exemplo, nessa partezinha aqui tem a função de você clicar nessa parte, porque a gente precisa avançar, né? Você consegue meio que burlar isso, só que na hora não vai ter, né? Porque a gente precisa barrar a entrada do usuário. Aí qualquer dúvida é só mandar pra gente. E aí, aqui você consegue avançar e usar como se fosse realmente um aplicativo (TIC 1).

(...)

| **Transcrição das reuniões e encontros realizados em Júlio, agosto, setembro, outubro, novembro e dezembro de 2024, janeiro e fevereiro de 2025** |
| --- |

Enquanto isso, deixa eu conversar sobre uma coisa. A gente tá seguindo aquela parte do Figma. Que é aquela parte do design e da prototipação. Então a gente acabou aquela parte de prototipação, são aquelas telinhas, mas aquela parte do módulo que a gente conversou faz bastante tempo, é sobre o módulo de administrativo, da parte do administrador, que o usuário tem que aprovar o login do outro usuário, não é qualquer pessoa que pode se cadastrar no aplicativo. A gente está seguindo essa ideia. Essa aqui é a tela de login. Essa aqui é a tela do menu principal do aplicativo. A parte da home, a parte dos projetos. Essa aqui é a parte da evolução do paciente e a parte de menu. E quando você entra no projeto, tem aquelas opções que a gente conversou. Isso aqui sobre cor e tudo mais, eu tô deixando mais pro final esses detalhes e fazendo mais o principal. Aí aqui a gente tem aquelas opções de dados do paciente. Vão ter essas opções. Isso aqui a gente conseguiu fazer de um jeito dinâmico. (...) Mas até agora, é isso que a gente tem. O design da prototipação, mas sobre o desenvolvimento e criação de fato do aplicativo ele está parado aqui. Deve dizer que isso aqui é uns 30% (TIC 1).

Ô TIC 1, deixa eu te falar, então essa parte aqui é a prototipação, não é? (Pesquisador).

Não, a prototipação hoje em dia acabou. É o menu do jeito que ele vai ficar. Entendeu? (TIC 1).

Entendi (Pesquisador).

O design mais refinado. Aqui a gente já tem sombra nas coisas, a gente tem os cards. Aí tem alguma coisinha, mas é assim que a gente trabalha. A gente cria a tela, cria a função dela. E aí, por exemplo, essa aqui que é uma tela que está em construção, a gente agora no começo, você pega tantos detalhes disso, a gente vai primeiro funcionar para depois deixar ela num design mais... (TIC 1).

Mais bonitinho, mais agradável, igual a esse aqui (Pesquisador).

Mas por enquanto é isso (TIC 1).

Ok (Pesquisador).

(...)

Se chama Android Studio deixa eu te mostrar, essa aqui é a parte de buildar. Buildar é tipo dar um play, sabe? (TIC 1).

O que que é Buildar? (Pesquisador).

Buildar é quando você co-build, é construção... É quando você monta as coisas, por exemplo aqui você escreve o código e aí você quer ver o resultado dele. Aí você builda, entendeu? (TIC 1).

Entendi. É assim que funciona então. Assim, só pra eu entender, nessa parte já tá o desenvolvimento, então... (Pesquisador).

Sim, a gente tá no desenvolvimento, a gente tá na etapa final (TIC 1).

Aí, quando terminar, aí que vai ter aquela ideia de...do...que o TIC 3 falou, de ter o link do aplicativo que consegue ver, usar em qualquer... em qualquer dispositivo (Pesquisador).

Aqui, igual eu te mostrei, aqui é só a tela, imagina que é só o dispositivo que a gente está acessando. Esse é um dispositivo Android, mas essa tela vai ser igual se você entrar no seu celular, independente de qualquer um, no link no site. Aqui é como se estivesse no aplicativo para a gente conseguir visualizar o que está acontecendo (TIC 1).

Entendi (Pesquisador).

Mas por enquanto é isso (TIC 1).

(...)

O webapp está sendo desenvolvido no software Visual Studio Code com linguagem e framework: Darte e Flutter, Banco de dados: Firestore, Authentication e Storage (TIC 1).

Mercado assim, é preciso ser pelo menos 6 dias. Nossa CPF que vem a profissão a que ela cedeu (...). Aqui também tem a foto, aí por enquanto a gente tirou a foto, eu estou arrumando pra escolher (TIC 2).

Que legal (Pesquisador).

Esse telefone, cadastro, você tá arrumando o banco (TIC 2). Vai andar pro lugar, vai cadastrar isso aqui? Mesmo esse é o cadastro da profissional, ok, é isso mesmo (Pesquisador).

Aí cadastraria isso mesmo. Viu? Eu vou ver, o “cara” vai dar pra logar. Então... seria a página principal (...). Eu apresentei todos os projetos e no caso já tem um projeto (TIC 2).

Entendi (TIC 2).

E também coisa de pesquisa. Eu tava fazendo isso aqui, ó, cadastrar paciente. Ele teve o nome, data de nascimento, CNS, mail, número de telefone, gênero, profissão, salário, residência, foto também e que as outras informações, elas viriam aqui também ou seria depois? (TIC 1).

Como assim deixa eu ver? Talvez de apoio? (TIC 2).

É no cadastro também. Isso deixa eu só sobe um pouquinho abaixo um pouco só para mim ver isso aí mesmo. Outra informações principal é um endereço, um técnico de referência. Isso mesmo, isso (Pesquisador).

Aí eu deixei... Quando ele cria essa conta, a senha dele vai ser os 4 últimos dígitos do telefone aí foi a primeira login que eu falo, tá vendo aqui? Primeiro acesso, sim. Ah, do ilustrador também. Que esse aqui seria o até do administrador, por enquanto. E os profissionais que já são aceitos e os que estão pendentes (TIC 2).

Então pode falar... (Pesquisador).

Aqui, no caso, ó, ficaria é se aceitasse. Aí clicaria em abrir uma telinha com todas as informações dele, você poderia recusar ou aceitar e que os que já seria normal já estaria lá (TIC 2).

E aí cada como por exemplo, a gente vai ter, o administrador vai ter que acessar, esse é autorizar cada profissional, né? Isso aí tá, isso legal, né? E futuramente, como, por exemplo, pode dividir esse administrador como por exemplo, o serviço também não é sim. Mas agora desse jeito está ótimo. Deixa eu só ver a home do aplicativo, não (Pesquisador).

Entra isso (TIC 1).

Estou bem velozinho. Aí tenho a apresentação principal, né? Aqui vai ter o vídeo que eu vou mandar para vocês. É aqui, né? (Pesquisador).

É a parte de projetos, essa evolução, não fiz parte (TIC 2).

Isso e deixa eu, deixa eu ver. Da parte dos projetos, ficou legal aqui você vai colocar a estrutura adicional, projeto. Isso, adiciona (Pesquisador).

Cadastrar. O paciente. Isso CNS é mais efetivo? Cartão do SUS (TIC 2).

Né, esse é o cadastro do paciente, não é? (Pesquisador).

É do paciente. Vai dar certo? Deu paciente, só que aqui não tá mostrando, mas ele tá cadastrado no banco. Mais nova. Ele já vem pra cá, ó, está cadastrado que era o Paulo, já cadastro todas as informações (TIC 2).

E a senha, que é os últimos 2 de telefone? Aí depois que ele cadastrar, ele vai gente mandar uma tabela. Que vai ser? (TIC 2).

(...) (Pesquisador).

Pacientes normais, é isso, por enquanto (TIC 2).

É isso, tá bom, tá legal, tá? É isso que é esperar. Tem alguma dúvida? (Pesquisador).

Até agora não, mas daqui um pouquinho. Vai surgindo, tá surgindo (TIC 1).

Tá, vocês acham que o quanto? (Pesquisador).

Quantos dias a gente pode se ver de novo? 15 dias (TIC 1 e TIC 2).

Então, ok, tá, deixa eu, eu vou compartilhar. É que eu sintetizei as nossas reuniões que a gente teve. Pra pra ver se a gente consegue. Eu fiz um é antes, me fala um pouquinho assim, como que tá sendo o desenvolvimento? É como que é como que, o que que vocês estão fazendo, em qual aplicativo, o que que é? Como se você fosse pudesse descrever vocês...Como que seria para uma pessoa leiga, o que que vocês estão fazendo, eu acho?... (Pesquisador).

Que o essa aqui é a parte onde a gente pega o design que foi criado e a gente implementa ele via código, sabe? A gente constrói de verdade. Agora a gente adiciona as funcionalidades, cadastrar um usuário. Antes era só uma imagem. Agora, de fato, a gente tem um formulário, a gente coloca as informações desse formulário e os dados vão para um banco de dados. E depois a gente pega esses dados, entendeu? Agora é como se fosse ganhando vida, o projeto utilizável de verdade (TIC 1).

Seria a (...) a programação sim do desenvolvimento da programação e aonde que como que se chama o software... recurso (Pesquisador).

A linguagem de framework... É assim a tecnologia que a gente tá usando pra construir isso. Sabe, é como se fosse uma ferramenta. Existem vários tipos de ferramentas. E aí a gente escolheu essas pra construir (TIC 1).

Esse aplicativo é a estrutura... (Pesquisador).

Tecnologias também é como se fosse. Como se a gente pegasse o Dart, que é uma coisa mais bruta, e fizesse coisas mais para facilitar o desenvolvimento. Assim são ferramentas (TIC 1).

Ok, muito bem. Vocês escreveram maravilhosamente. Eu fiz uma tabela e não estou conseguindo colocar no nosso chat. Deixa eu tentar de novo. Eu vou enviar lá pro WhatsApp, tá? Vê se você consegue. É que eu sintetizei aquelas as reuniões que a gente teve, consegue colocar aí pra gente ver. Olha, eu acho isso. Eu tentei, que a gente teve aquelas reuniões de discutindo e eu, eu, eu, eu tentei estruturar. Porque eu acho que aqui ficou bem legal pra gente entender o aplicativo, né? Na home, né? Que vem o vídeo curto que a gente decidiu tá lá, que era aquele excluía aquele botão start, que não tava legal. Então olha o cadastro, que foi o que você mostrou que tem, o do paciente, do profissional, não é? A gente lembra que a gente ficou que vai fazer o suporte do usuário. Onde vai ter é as referências de saúde mental. A legislação e o registro da produtividade. Aí vem pro projeto, que é os tópicos. Não é que vocês tinham colocado a avaliação que ficou? Diagnóstico não é metas, intervenções, pactuação e evolução, tá? (Pesquisador).

E eu acho que tem mais... (TIC 1).

Qual que é? (Pesquisador).

Deixa eu abrir aqui o filme na rapidão? Da quantidade, eu acho que a quantidade é um maior ou a gente tem avaliação correta, metas... correto, pactuações correto, agenda de estudo de caso? (TIC 1).

Isso a agenda de estudo de caso (Pesquisador).

Isto. É dentro de caso. Tem metas de cuidado em saúde mental, que é metas, né? E também aqui a gente tem dados do paciente colocar aqui. O jeito que eu que eu vou compartilhando fico mais fácil de visualizar. Vou colocar um aqui, ó, essa partezinha aqui é essa parte, correto? (TIC 1).

Isso, dados do paciente, diagnóstico situacional, metas, intervenções, pactuação, agenda de estudo de caso e reabilitação. É isso? Isso mesmo? (Pesquisador).

Eu acho que faltou só diagnóstico, né, diagnóstico institucional e saúde mental (TIC 1).

Isso que é tá no lugar da avaliação. Aí eu vou substituir, então tá (TIC 1).

Ok (Pesquisador).

E lá... de funções que a gente discutiu foi o compartilhar, lembra, que é compartilhar os tópicos relatório com o que vocês fizeram, um recurso do WhatsApp, das redes sociais e a busca do dispositivo pelo endereço (Pesquisador).

Então era só mais esse e o banco de dados que foi a criptografia, se ligar também (TIC 1).

(...)

Como que é o nome do banco de dados? (Pesquisador). Ele chama firebase. Foi isso que eu digitei aqui no chat.

Agora? (Pesquisador).

É se eu posso se aprofundar mais também? (Pesquisador).

Assim, firebase é o nome do esse que é o nome do produto. Dentro desse produto, a gente usa 3 funções deles, entendeu? Tem mais funções, só que a gente só usa 3 (TIC 1).

E quais são essas funções que usam? (Pesquisador).

E aí é sempre firebase antes de cada. A gente pode mandar depois pra que cada um serve (TIC 1).

Ok (...). Então é isso, eu vou mandar pra vocês o termo das condições de uso do aplicativo, tá? Eu não enviei. Ele tá em PDF, OK! (Pesquisador).

A gente pode assinar... (TIC 1).

Não, não é o termo de pra vocês assinar não é do não tem lá aquele não tem um link. Antes da pessoa isso do cadastro que lá quando você mostrou antes que se aceita usar o app (Pesquisador).

Esse aqui, ó (TIC 2).

Isso quando você entrou?... Não tem lá, não tem em algum lugar de política de então proteção e viabilidade ali. Concordo com isso. Essa política aqui eu vou mandar o termo pra adicionar, tá bom? Proteção de dados, que seria a mesma coisa, é a mesma (Pesquisador).

(A mesma) Coisa (TIC 1).

É a mesma coisa, só pra ter ciência da pessoa ir aceitar (Termo de Política de Privacidade e Segurança de Dados). Tá, é a mesma. É a mesma coisa, tá aí eu vou dar só mais uma lida. Aí eu envio pra vocês, pode ser? (Pesquisador).

Pode ser. (TIC1).

(...)

O que a gente consegue fazer depois também?... quando tiver assim a maior da maior parte das funcionalidades, a gente consegue, porque como que funciona a gente é como se a gente desse play nesse software que a gente usa, aí aparece essa tela que o TIC 2 está mexendo, entendeu? Como se fosse o resultado do nosso código E ele é feito através de um link, só que esse link ele fica só na máquina do TIC 2... e aí depois tem como também a gente colocar esse link pra de um jeito que você consegue acessar. Então você também consegue ver através do seu celular como que ele tá ficando, que aí já tem o uso mais real dele. (TIC 1).

Legal isso (Pesquisador).

Também já dá para ver... (TIC 1).

Pra fazer. Vocês acham que até o fim do ano de 2024 conseguem terminar? Ah, então ótimo, por que o que que acontece? Lembra que eu falei que a gente. Eu vou encaminhar o link pro pra profissionais de tecnologia, que eles vão aplicar uma escala pra ver de forma técnica como que tá o aplicativo, né? Então, então é isso aí. Eu queria é que eu tava olhando? Porque aí senão a gente vai ficar muito apertado, porque se faz no final, aí eu consigo resolver isso em janeiro. Aí faz os ajustes e em fevereiro fecha, tá ok? Eu é, vou, estou. Tudo que a gente está conversando, eu estou ajudando o artigo, mas quando chegar no fim o artigo, eu vou enviar para vocês, tá bom? Então vamos só marcar pra próxima, daqui 15 dias, eu acho que... (Pesquisador).

Podia (TIC 1).

(...)

Show de bola, muito legal, muito bonito. A gente vê, não é? Qualquer coisa estou disponível, pode chamar. Máximo, se eu não responder de imediato é porque eu vou estar pensando, tá bom aí ó, já responda, eu respondo de novo, eu vou te mandar, vou mandar para o TIC 1 o termo, só vou ler e assim que a gente terminar o vídeo, eu mando que o vídeo é para colocar lá naquele link que tá tá, mas de então aguardo vocês e que adianta esse projeto? E está muito legal. Parabéns, tá bom (Pesquisador).

Grande abraço, tchau (TIC 1).

Tchau (TIC 2).

(...)

Tá conseguindo ver? (TIC 1).

Tô sim (Pesquisador).

(...) A gente vai te mostrar aí de pé que tá (TIC 2).

Beleza (Pesquisador).

Veio uma coisa, agora eu entender o que tava ali, eu não sei não. Tem outra tela aí (TIC 2).

Já (Pesquisador).

Coloca pequeno (TIC 1).

Essa aí é a simulação. É a tela de login que a gente mudou também (TIC 2).

E é a simulação de como que fica no celular. Que aí ele simula a tela de um celular, entendeu? (TIC 1).

Eu fiz o login, aqui tá o vídeo, né? Você clica nesse verdinho aqui e tá o vídeo (TIC 2). E os projetos que são as pessoas, né? (Pesquisador).

Aqui tudo só me ensinando a ver, pra testar. Aí que funciona já. E aqui tudo tá cadastrando agora. E eu fiz um negócio que tipo, pra compartilhar um projeto. Ficou numa web assim, normal, ó. Ele copia o código da pessoa, que no caso é... só que na hora de criar vai ser um código mais grande e pra celular ele abre embaixo, conhecendo os aplicativos são dados pacientes que eu tive que usar, normal uma, que eu já tinha... aqui diagnóstico, agora tá cadastrando cadastra, aqui cadastra (...). Testar também eu já deixei ele na nuvem. Então dá pra você entrar aqui e testar. Vai ser melhor pra gente (TIC 2).

Ok. Só me manda o login (Pesquisador).

Vai estar aqui. Também outra coisa é a evolução, que também eu já fiz. Coloca uma coisa, ele aparece aqui... também a pesquisa...Aqui no caso, eu fiz...aí ele aparece também. Aí caso esse aqui, seria na hora que a gente foi compartilhar o projeto. Eu compartilhava, copiava o link, que no caso já abriu, olha, é aquele jeito. (...) Aí você viria aqui e pesquisava (TIC 2).

(...) Essa forma de compartilhar é como se eles tivessem um código, aí você compartilha o código da outra pessoa, e aí naquele pesquisar, você se vincula a ela (TIC 1).

Acho melhor mostrar o mapa também. O mapa (Recurso de busca de serviços RAPS) a gente teve ideia assim. Acho que ficou mais fácil. Porque marcar no mapa eu acho que não tem como. É um negócio mais complicado. O que a gente tem como é marcar um por um. Tipo assim ó, se a gente tava... Aí aparece tudo que tem a ver com saúde mental (TIC 2).

Mais é isso! (Pesquisador).

Só que no mapa não tem como mostrar (TIC 2).

Não precisa, não precisa mostrar no mapa. Desse jeito é isso que a gente precisa mesmo. Mostra as informações, as informações buscadas. Isso mesmo (Pesquisador).

(...)

Aí a gente deixa o sua telinha mais bonitinha. O que dá pra fazer também, que eu acho que fica legal, é a gente conseguir também colocar uma setinha, se a pessoa clica nessa seta, aí abre esse endereço no Google Maps (TIC 1).

Perfeito, é isso. Ótimo! (Pesquisador).

Tem também a parte do cara, né? do ADM, né? (TIC 2).

É (Pesquisador).

Esse aí é o módulo que é a conta que cria as outras contas, tanto de administrador quanto profissional. A gente passa o link, a gente passa as contas e o e-mail senha de cada (TIC 1).

Combinado, aí vocês me mandam o link e a conta. Mas gente olha é isso mesmo a proposta agora é só finalizar é isso é coerente! Eu vou enviar... eu tô terminando lá das referências... Na produtividade tipo, se for uma opção que ela não tem aquelas bolinhas, tipo, não que vocês podem ser livre, o que vão escolher, né? Que a gente já marca a opção. Só que aí tem que ter uma funcionalidade que toda vez que ela entrar no aplicativo, ela pode estar sempre escolhendo. Faz sentido o que eu tô querendo dizer? Como, por exemplo, ela entrou no aplicativo e foi lá e lá vai ter, como por exemplo, atendimento individual. Aí ela foi lá, marcou, ela saiu do aplicativo, entrou de novo e ela percebeu que, ó, registrou, ela fez um atendimento em grupo, aí ela marcou, saiu e salvou. Entende? (Pesquisador).

(...)

Onde você acha que está? (Pesquisador).

Aos 90%? (TIC 1).

Ah tá, agora é só o você ver mesmo (TIC 2).

Faltam os erros pra gente... (Finalizar)... (TIC 2).

A gente arrumando. Mas olha, tá... A ideia ficou legal, ficou perfeito. Se tivesse como... Não tem aquela... Lá no meu protótipo. Aquele cerebrinho que tem? Tipo aquele loginho? (Pesquisador).

Sim, sim (TIC 1).

Sim (TIC 2).

(...)

Legal, legal, aí eu vou entrar mas não tem muita coisa é só, só, a gente alinhar o que tá é... (Pesquisador).

(...) Pode fuçar (TIC 2).

Eu vou fuçar... só me manda o link lá no whatsapp e o meu e o login para mim..., mas olha... tá incrível, não sei nem o que dizer! ficou muito legal essa versatilidade! se ele estiver no aplicativo fica no aplicativo, se for para o computador fica maior. Isso ficou top! Legal, legal, legal! (Pesquisador).

(...)

Plantar umas coisas, bater um papo. Então, a gente fez as alterações (TIC 1).

É... (Pesquisador).

Agora eu acho interessante a gente já fazer um processo, ... que é a gente pega o que está no nosso servidor, que é esse aplicativo, e a gente joga para aquele servidor que você alugou. E aí esse processo, ele é demoradinho. Antes, demorava umas 12 horas. Eu acredito que hoje seja antes. Acho que umas 6 assim. Se a gente fizer agora pela manhã, de tarde já vai estar funcionando. E aí eu preciso, eu vou até compartilhar a tela aqui, que fica mais fácil pra gente. Tá dando pra ver? (TIC 1).

(...)

E isso significa o quê? Que tá hospedado nesse site? (Pesquisador).

Isso, é assim ó. Acho que se eu explicar fica mais fácil. A gente tem aqui o local é a nossa casa, basicamente. O aplicativo está aqui agora. O que a gente vai fazer? A gente vai mandar esse aplicativo para o servidor público. Que é o www.com.br, por exemplo. Ou só... ponto... com, nesse nosso caso. E aí, essa passagem do aplicativo da nossa casa para o servidor chama o processo que a gente vai fazer agora. Que é... hospedagem. Que aí a gente tira ele daqui e a gente coloca ele num lugar que todas as pessoas conseguem visualizar. Que é onde você comprou o domínio. Aí esse processo. Em média, isso aqui demorava um dia. Eu acho que é um prazo que eles dão. Mas sempre costuma ser mais rápido. Não costuma ser tudo isso, não. Então hoje já... já cai (TIC 1).

Aí quando, por exemplo, agora, quando for acessar todo o recurso do aplicativo ele vai ficar aqui, não mais onde estava nesse computador, é isso? (Pesquisador).

Em vez de ele ficar aqui no computador, por exemplo, se acontecer alguma coisa, sei lá, queimar, não sei, ele sai daqui e vai para um outro local, entendeu? A gente pega todos os arquivos, joga para esse servidor público aqui, que no caso é a HostGator, né? E aí eles colocam... Aí quando alguém digita no Google, por exemplo, qualquer navegador, na verdade, Safari, para quem tem iPhone, reabilitasocial.com, aí ele sempre vai entrar. Eu criei também, aqui no aplicativo, uma... deixa eu ver se eu estou compartilhando essa tela, eu acho que eu estou. (...) Então, o que eu recomendo? Eu recomendo eu, você e o TIC 2... a gente ter uma conta nossa de administrador porque a gente consegue olhar o que está acontecendo no aplicativo. como se a gente fosse uma etapa acima de permissão, sabe? A gente pode fazer coisas, se a gente deve fazer coisas que os usuários comuns não fazem. Então aí eu criei uma conta pra você também, depois você até troca a senha, porque a senha ela tá... tá o seu e-mail, e aí isso você consegue entrar como administrador (TIC 1).

Combinado, isso a gente acordou, tá tudo certo. Agora eu te falei que tem essa etapa da minha pesquisa que eles vão avaliar, mas eles não vão mexer dentro, eles só vão entrar no aplicativo e ver se está funcionando ou não e vão responder um questionário. É isso que eu quero saber com vocês, o que você acha cômodo ou importante criar acesso para os avaliadores ou eles mesmo criam quando entrar? Eles vão conseguir ir com o e-mail e a senha e já entrar? (Pesquisador).

Ô, ... uma outra dúvida também. É, por exemplo, a gente pensou um código, né? Isso aqui é o resultado de um código. Esse código, você precisa dele em algum lugar também? (TIC 1).

O que que é um código? (Pesquisador).

Um código, ó, eu vou até compartilhar, porque fica tudo mais fácil mostrando isso. Vou tirar essa tela daqui. Eu vou te dar um panorama geralzão do que é um desenvolvimento. Aqui é o aplicativo de verdade, vamos dizer. Tudo que a gente escreve, por exemplo, essa aqui é a tela de login. Olá, bem-vindo ao Reabilita Social. Se eu colocar aqui, teste, o resultado do código é o aplicativo. Então apareceu o teste aqui. Isso aqui é assim, as funções, é como ela foi configurar, é tudo por baixo dos panos. Isso aqui gera o aplicativo que a gente está usando, que a gente está abrindo. Você precisa desse código em algum lugar? (TIC 1).

Não, não vou precisar nisso na minha tese, não. Não vou precisar. do código, mas eu acho que, assim, esse código tem que ser salvo, tem que colocar em algum hospedeiro, alguma coisa? (Pesquisador).

Então, existe uma plataforma que a gente usa, que a gente salva lá, é uma questão de segurança, sabe? Como se fosse um drive. A gente sempre salva códigos nesse local. Pra deixar salvo mesmo, se a gente precisar de algum dia, precisar mexer, a gente não tem na nossa máquina. A gente tem nesse local, que é gratuito, a gente deixa ele lá e 100% seguro. Porque se a gente quiser retomar um dia, independente da data, a gente consegue parar de onde deixou. O que eu pergunto é, por ter essa validação, para as pessoas usarem, profissionais da tecnologia, não vai ser tão fundo assim. Então, eles não vão olhar código, esse tipo de coisa. A gente precisa mostrar para eles (TIC 1).

Então, de olhar do código, eu acredito que não. Depois, quando a gente terminar isso, antes de ir para eu vou marcar uma reunião com você, para a gente mostrar o instrumento e a gente chama, eu vou enviar para os profissionais de tecnologia e a gente vê o que eles solicitam nesse caso, porque é baseado nos instrumentos... Agora, o código é importante a gente salvar, porque o que acontece? Lembra que eu falei que quando estiver pronto a gente vai registrar nos nossos nomes no INPI, que é o registro do código. Eu não sei, porque eles pedem um monte de coisa, eu acho que eles vão pedir. Então, é bom a gente guardar, porque quando eu terminar o doutorado, e se o aplicativo passar por todas as validações, a gente vai entrar e ter aquela conversa, pra gente tentar aplicar, e quem sabe que esse aplicativo não começa a gerar financeiro. Então, é importante a gente manter toda a nossa parceria, e tudo que tem do aplicativo, porque ele vai entrar na etapa de funcionamento, e no funcionamento de aprimoramento, e pode gerar valores, não é? Perfeito. Entendeu? Então é isso agora. Porque eu acho que ficou muito assim, tá? Parece que tá bem legal que tá aplicativo. Agora a gente pensa, se passou, validou, produziu o produto, tá? A primeira etapa agora é quando vocês fecharem, é... Aí eu vou sentar com vocês que a gente vai registrar o app. Aí vamos ver. Vamos registrar no nosso nome, como acordou. voam para essa validação e vai ter a última validação com mais de 100 profissionais que eles vão testar, mas essa é até o final do ano... Aí para o pós-doutorado é a implementação a gente tá no final do doutorado nas últimas etapas até maio do ano que vem (Pesquisador).

(...)

Ah, então isso é muito legal. Porque é um interesse que a gente pode chegar de querer jogar pra Play Store. Você entende? (TIC 1).

Sim, com certeza. Essa é uma das vantagens da tecnologia que a gente usou, que se chama multiplataforma, que a gente cria um código e nesse código a gente pode usar, igual a gente está usando para um navegador, para um Chrome, para um Safari, para qualquer navegador, o mesmo código, a gente pode usar, jogar para o iPhone e jogar para o Android, que é para a Play Store. Então é uma coisa mais reutilizável. Os recursos, a gente está no perfil, ajuda e suporte, coloquei o restante, as portarias, paciente evolução esse aqui eu também dei uma mudada no design que eu acho que tinha alguns errinhos aqui eu coloquei maior a foto do paciente e um titulozinho... Eu achei bem legal participar, eu acho um projeto muito interessante (TIC 1).

O que você achou interessante? (Pesquisador).

Eu acho que mais um tema sabe porque como na área de desenvolvimento da tecnologia normalmente o que a gente faz a gente faz site a gente faz sistema de estoque sabe tem muito e concentra num tempo diferente por exemplo esse de saúde mental é umas coisas diferentes de se trabalhar. para um tema mais legal, como se fosse mais útil, sabe? É mais interessante fazer isso aqui para um usuário que é de saúde mental e tudo mais do que para um de estoque, por exemplo. Parece que é uma coisa mais (ampla)... (TIC 1).

Ai, que legal (Pesquisador).

É isso. Depois eu te dou o seu acesso certinho. Aí você consegue testar, criar usuário. Aqui você pode fazer o que você quiser. Pode criar usuário, pode criar tudo. Porque depois a gente consegue apagar esses dados. Então pode testar, pode criar qualquer coisa pra ver se tá funcionando. Pode ser bem livre aqui. Eu vou... Aí você pode esperar quando já estiver lá naquela plataforma pra você me mandar o acesso, tá? (TIC 1).

Sim, sim. Tranquilo (Pesquisador).

Perfeito (TIC 1).

Já, está bem bonito, bem agradável. Vocês foram muito... com uma expertise... uma aprendizagem muito grande. Eu espero que a gente feche essas etapas e pode construir mais parcerias pra gente realmente fazer isso funcionar e quem sabe ser uma fonte de renda pra gente (Pesquisador).

(...)

Apresentação entendeu, aí... e o que então, combinado? (Pesquisador).

Combinado, combinado tchau, tchau (TIC 1).

Tchau, tchau, um abraço (Pesquisador).
